# EGFR inhibits TNF-α-mediated pathway by phosphorylating TNFR1 at tyrosine 360 and 401

**DOI:** 10.1038/s41418-024-01316-3

**Published:** 2024-05-24

**Authors:** Young Woo Nam, June-Ha Shin, Seongmi Kim, Chi Hyun Hwang, Choong-Sil Lee, Gyuho Hwang, Hwa-Ryeon Kim, Jae-Seok Roe, Jaewhan Song

**Affiliations:** Department of Biochemistry, College of Life Science and Technology, Institute for Bio-medical Convergence Science and Technology, Yonsei University, Seoul, Republic of Korea

**Keywords:** Molecular biology, Cell biology

## Abstract

Tumour necrosis factor receptor 1 (TNFR1) induces the nuclear factor kappa-B (NF-κB) signalling pathway and regulated cell death processes when TNF-α ligates with it. Although mechanisms regulating the downstream pathways of TNFR1 have been elucidated, the direct regulation of TNFR1 itself is not well known. In this study, we showed that the kinase domain of the epidermal growth factor receptor (EGFR) regulates NF-κB signalling and TNF-α-induced cell death by directly phosphorylating TNFR1 at Tyr 360 and 401 in its death domain. In contrast, EGFR inhibition by EGFR inhibitors, such as erlotinib and gefitinib, prevented their interaction. Once TNFR1 is phosphorylated, its death domain induces the suppression of the NF-κB pathways, complex II-mediated apoptosis, or necrosome-dependent necroptosis. Physiologically, in mouse models, EGF treatment mitigates TNF-α-dependent necroptotic skin inflammation induced by treatment with IAP and caspase inhibitors. Our study revealed a novel role for EGFR in directly regulating TNF-α-related pathways.

## Introduction

Tumour necrosis factor (TNF-α) is one of the most extensively studied cytokines in the body. TNF-α signalling induces nuclear factor kappa-B (NF-κB) signalling, apoptosis, or necroptosis in the cell, which lead to inflammation, autoimmune diseases, cell survival and cell death [1–4]. Mechanistically, when TNF-α binds TNF receptor 1 (TNFR1), TNFR1 forms trimer complexes, which recruit receptor interacting protein kinase 1 (RIPK1) and TNFR1 associated death domain (TRADD) protein via their death domain followed by TNF receptor-associate factor 2 (TRAF2), cellular inhibitor of apoptosis proteins 1 and 2 (cIAP1/2) and linear ubiquitin chain assembly complex (LUBAC). The complex formed by these proteins is collectively known as complex I, and it initiates the downstream signalling of various TNFR1 pathways [2–4]. The ubiquitin chains of RIPK1 formed by cIAP1/2 and LUBAC complexes further function as a platform for the recruitment of IκB kinase (IKK) and TGF-β-activated kinase 1 (TAK1) complexes which induce NF-κB signalling to produce inflammation or cell survival signals [2–4]. When NF-κB target gene translation is inhibited, for example, by cycloheximide treatment, RIPK1-independent complex IIa, composed of TRADD, Fas-associated death domain protein (FADD) and caspase-8, is formed. In contrast, complex I is suppressed when the ubiquitination of RIPK1 or TAK1 is inhibited by IAP inhibitors. Simultaneously, 5Z-7-ox prompts the formation of complex IIb, which is composed of RIPK1, FADD and caspase-8. When complexes IIa and IIb are activated, caspase-8 cleavage is induced. Other caspases are subsequently activated, leading to apoptosis [1–4]. When the protease activity of caspase-8 is inhibited, necrosomes comprising RIPK1, RIPK3 and mixed lineage kinase domain-like protein (MLKL) are formed, leading to the activation of each component by phosphorylation. RIPK3 phosphorylates MLKL, which forms pore complexes that permeabilise the plasma membrane and lead to necroptosis [1–4].

Researchers have studied the post-translational regulatory processes of the TNFR1-mediated NF-κB signalling pathway and cell death. Phosphorylation is one of the most extensively studied key processes in the TNFR1-mediated signalling pathway. When PKA phosphorylates TNFR1 at Thr 411 and 417, the interactions between TNFR1, Janus kinase 2 (JAK2), c-Src, growth factor receptor bound protein 2 (Grb2) and p85 are suppressed [5]. The phosphorylation of TNFR1 at Ser 381 and of TRADD at Ser 215 and Ser 296 is required for the interaction between TNFR1 and TRADD [6]. RIPK1 phosphorylation by IKKα/β, TBK1 or MK2 suppresses TNF-α-induced cell death by reducing RIPK1 kinase activity [7–14]. TRAF2 phosphorylation by TBK1, IKKε and PKCζ promotes IKK activation [15–17]. IKKβ and GSK-3β phosphorylate NEMO and suppress NF-κB activation [18, 19]. Moreover, the phosphorylation of caspase-8 by Cdk1, Plk1, Src and ERK inhibits apoptosis by suppressing caspase-8 activation [20–24]. While the phosphorylation of Thr 152 on caspase-3 promotes apoptosis, the phosphorylation of Ser 150 suppresses apoptosis [25]. In addition, the phosphorylation of caspase-3 by PKCδ also suppresses apoptosis [26]. While the depletion of CK1 decreases RIPK3 Ser 227 phosphorylation, phosphatase Ppmb1 dephosphorylates RIPK3 at Ser 227 to suppress RIPK3 kinase activity [27–29]. MLKL is phosphorylated at Thy 376 by the TAM kinase family, as well as by RIPK3, to promote MLKL oligomerization [30].

Epidermal growth factor receptor (EGFR) is a member of the EGFR superfamily and a receptor tyrosine kinase that regulates cell survival and proliferation [31, 32]. When EGF binds to EGFR, it forms a homodimer or heterodimer with human epidermal growth factor receptor 2 (HER2) via its transmembrane domains. This dimerisation leads to EGFR phosphorylation, which in turn leads to its interaction with downstream proteins containing the SH domain. EGFR activation induces the PI3K-AKT-mTOR and RAS-RAF-MEK-ERK axes to induce gene transcription for cell growth and proliferation [31, 32]. The crosstalk between EGFR and TNFR1 signalling has been previously studied [33–38]. TNFR1 inhibits EGFR activation via its death domain [33]. However, other studies have reported that while EGFR activates TNFR1, TNFR1 also activates EGFR [35]. In therapeutic models, EGFR inhibition promotes TNF-α activation in glioblastoma [38] and increases TNF-α-induced lung epithelial cell apoptosis and pulmonary injury [36]. In contrast, erlotinib, an EGFR inhibitor, reduced lung inflammation in an advanced lung cancer inflammation index (ALI) model by suppressing RIPK3-dependent necroptosis [37]. However, the crosstalk between TNFR1 and EGFR remains controversial and has not been addressed in detail.

Here, we observed that active EGFR or EGFR activated by EGF treatment prompted an interaction between the TNFR1 death domain and the EGFR tyrosine kinase domain, causing phosphorylation of TNFR1 at Tyr 360 and Tyr 401. These phosphorylation processes lead to the suppression of subsequent interaction between TNFR1 and RIPK1, which resulted in a decrease in RIPK1 polyubiquitination that downregulated the TNF-α signalling pathway. In contrast, EGFR inhibition restricts the interaction between EGFR and TNFR1 and increases complex I formation, which may lead to the activation of complex II-dependent apoptosis or necrosome-mediated necroptosis. In a mouse skin necroptotic inflammation model induced by ASTX660 and emricasan, EGF treatment significantly decreased skin lesions by reducing the activation of necroptotic factors, such as p-RIPK3 and p-MLKL. We believe the identification of EGFR’s potential role in regulating TNFR1 will help researchers develop new therapeutic drugs in the future.

## Materials and methods

### Cell culture, plasmids, and transfection

HT-29, HCT-116 and MDA-MB-231 cells were maintained in Roswell Park Memorial Institute (Cytiva, Marborough, USA) medium supplemented with 10% FBS (Cytiva, Marborough, USA) and 1% penicillin/streptomycin (Cytiva, Marborough, USA) in 5% CO2 at 37 °C. HeLa, 293T and Panc-1 cells were cultured in high-glucose Dulbecco’s modified Eagle’s medium (Cytiva, Marborough, USA) supplemented with 10% FBS (Cytiva, Marborough, USA) and 1% penicillin/streptomycin (Cytiva, Marborough, USA) in 5% CO2 at 37 °C.

EGFR and TNFR1 constructs were purchased from Addgene. The EGFR construct and mutants (K745A, 6YF, E-ECD, E-ΔKD, E-ΔECD and E-KD) were subcloned into the pCS5/3xHA vector using PCR. The TNFR1 construct and mutants (T-ECD, T-ΔDD, T-TM/ICD, T-ΔECD, T-DD, Y360D, Y401D, Y360/401D, Y360F, Y401F and Y360/401F) were generated using PCR into pCS5/3xFLAG and pMSCV-hygro vectors. pLKO.1puro-shTNFR1 was generated using oligo annealing and cloning into an empty vector using following primers: shTNFR1 F 5′-CCG GAG AAC CAG TAC CGG CAT TAT TCT CGA GAA TAA TGC CGG TAC TGG TTC TTT TTT G-3′ and shTNFR1 R 5′-AAT TCA AAA AAG AAC CAG TAC CGG CAT TAT TCT CGA GAA TAA TGC CGG TAC TGG TTC T-3′. The single-guide RNA (sgRNA) sequence targeting EGFR was designed using the CRISPR Design Web tool (http://www.e-crisp.org/E-CRISP/). The primers designed using the sequences are sgEGFR #1F 5′-CAC CGG AGT AAC AAG CTC ACG CAG T-3′, sgEGFR #1R 5′-AAA CAC TGC GTG AGC TTG TTA CTC C-3′, sgEGFR #4F 5′-CAC CGA AAT CCT GCA TGG CGC CGT G-3′ and sgEGFR #4R 5′-AAA CCA CGG CGC CAT GCA GGA TTT C-3′. The primers were cloned into lentiCRISPRv2 vector using GeCKO’s cloning protocol. Recombinant retroviruses and lentiviruses were produced by co-transfecting 293 T cells with retroviral and lentiviral vectors using PEI (Sigma-Aldrich). The number of stable cells was verified by immunoblotting. PEI (Sigma-Aldrich, St. Louis, MO, USA) was used to transfect plasmids into 293 T cells and Viafect (E4982, Promega, Madison, WI, USA) was used to transfect plasmids into HeLa cells. After 24 h, the cells were used to each experiment.

### Generation and validation of knockout and reconstitution cell lines

LentiCRISPRv2 vector was obtained from Addgene (Addgene plasmid #52961; Cambridge, MA, USA). The single-guide RNA (sgRNA) sequence targeting EGFR was designed using the CRISPR Design Web tool (http://www.e-crisp.org/E-CRISP/). The primers designed using the sequences are sgEGFR #1 F 5′-CAC CGG AGT AAC AAG CTC ACG CAG T-3′, sgEGFR #1R 5′-AAA CAC TGC GTG AGC TTG TTA CTC C-3′, sgEGFR #4 F 5′-CAC CGA AAT CCT GCA TGG CGC CGT G-3′ and sgEGFR #4 R 5′-AAA CCA CGG CGC CAT GCA GGA TTT C-3.′ The primers were cloned into lentiCRISPRv2 vector using GeCKO’s cloning protocol. LentiCRISPRv2-sgEGFR vectors and packaging plasmids were transfected in 293 T cells to produce lentiviruses. Then HeLa cells and HT-29 cells were infected with the lentivirus following 1 μg/mL puromycin treatment to select infected cells for 3 days. After puromycin selection, single-colony selection was performed to identify EGFR knockout cells, confirmed by immunoblotting.

To establish a TNFR1 reconstituted cell lines, pLKO.1 puro-shGFP and pLKO.1 puro-shTNFR1 vectors and packaging plasmids were transfected in 293T cells to produce lentiviruses. HeLa cells and HT-29 cells were infected with the lentiviruses following 1 μg/mL puromycin treatment to select infected cells for 3 days. After puromycin selection, immunoblotting was performed to identify TNFR1 knockdown cell lines. After that, 293T cells were transfected with pMSCVhygro-3xFLAG-TNFR1 WT or Y360/401D or Y360/401F, and packaging vectors to produce retroviruses. TNFR1 knockdown HeLa cells and HT-29 cells were transfected with the retroviruses and selected by 400 μg/mL hygromycin for 3 days. After selection, immunoblotting was performed to identify TNFR1 reconstituted cell lines.

### Reagents

The followed reagents were procured from the indicated companies: erlotinib (Sigma Aldrich, SML-2156-50MG), gefitinib (Cayman, 13166), EGF (Sigma Aldrich, E9644), dynasore (Sigma Aldrich, D7693), TNF-α (R&D systems, 210-TA-020), birinapant (Selleck, S7015), z-VAD-fmk (R&D system, FMK001), GSK’963 (Aobious, AOB9775), cycloheximide (Sigma Aldrich, C4859), rapamycin (Sigma Aldrich, R8781-200μl), wortmannin (Sigma Aldrich, W1628) and AZD6244 (Selleck, S1008).

### Immunofluorescence assay

For immunofluorescence staining, HT-29 and HeLa cells were cultured on coverslips in 12-well plates. Post treatment with the indicated reagents, the cells were fixed with 4% paraformaldehyde for 30 min at RT. Anti-EGFR (Cell Signalling, 4267S) (1:50) and anti-TNFR1 (R&D systems, AD225) (1:50) or Anti-HA (Santa Cruz, sc805) (1:50) and anti-FLAG (Sigma Aldrich, F3165) (1:50) were incorporated to each sample in immunofluorescence blocking buffer (PBS with 3% BSA, 1% saponin and 1% Triton X-100) and then incubated at 4 °C overnight. Each sample was washed thrice with PBS. Alexa Fluor 488-conjugated anti-goat (Invitrogen, A-10055) (1:100) and Alexa Fluor 594-conjugated anti-rabbit (Invitrogen, A-21207) (1:100) or Alexa Fluor 488-conjugated anti-mouse (Invitrogen, A-11029) (1:100) and Alexa Fluor 594-conjugated anti-rabbit antibodies (Invitrogen, A-21207) (1:100) were incorporated into each sample in immunofluorescence blocking buffer and incubated at RT for 1 h. The nuclei were stained with DAPI for 5 min and evaluated using LSM980 (Carl Zeiss, Oberkochen, Germany) and ZEN (blue edition) programme.

### RNA-seq and Data analyses of RNA-seq

The RNA-seq libraries were prepared with NEXTflex Rapid Directional mRNA-seq kit (catalogue no. NOVA-5138-11, PerkinElmer). Total 10 μg of RNA was extracted using QIAzol (catalogue no. 79306, QIAZEN) and isolated into mRNA through poly-(A) selection step. The mRNA was fragmentated and synthesised into cDNA. Adenylation and adapter ligation were performed, followed by PCR amplification to construct the RNA-seq libraries.

1. Identification of DEGs: Raw reads from RNA-seq were aligned using STAR mapping tool. The relative transcript abundances were measured in Reads Per Kilobase of transcript per Million mapped reads (RPKM) from Cufflinks. Among 200 genes which are up-regulated by TNF-α treatment, 128 genes were ranked by comparing their mean log2 fold change between the 4 experimental groups of interest.
2. GSEA: GSEA was performed according to the instructions. The gene-sets of MSigDB v7.0 and ‘NF-kB-dependent target’(Ngo et al., Cell Reports) were used for transcriptional difference between each treatment.
3. Gene ontology (GO) analysis of genes: A list of the defined 200 genes which were up-regulated by TNF-a treatment was used as an input for GO analysis with AmiGO tool.

### Quantitative reverse transcription-polymerase chain reaction (qRT-PCR)

Total RNA of HeLa cells was prepared using TRIzol reagent (Invitrogen, BRL-15596-018), and the complementary DNA (cDNA) was synthesised using 1 μg of total RNA. Subsequently, the cDNA samples were then subjected to analysis through real-time PCR using the QuantiTect SYBR Green PCR Kit and the Rotor-GeneQ 2plex system (Qiagen), employing the following primers: NFKBIA F 5′-CTC CGA GAC TTT CGA GGA AAT AC-3′, NFKBIA R 5′-GCC ATT GAA GTT GGT AGC CTT CA-3′, CXCL2 F 5′-GGC AGA AAG CTT GTC TCA ACC C-3′, CXCL2 F 5′-GGC AGA AAG CTT GTC TCA ACC C-3′, CXCL2 R 5′-CTC CTT CAG GAA CAG CCA CCA A-3′, CXCL3 F 5′-CCA CAC TCA AGA ATG GGA AG-3′, CXCL3 R 5′-CTG TCC CTA GAA AGC TGC TG-3′, GAPDH F 5′-TGT AGT TGA GGT CAA TGA AGG G-3′ and GAPDH R 5′- ACA TCG CTC AGA CAC CAT G-3′ Data are presented as mean ± standard deviation (S.D.), *n* = 3, with ns non-significance, *P < 0.05, **P < 0.01 and ***P < 0.001 at each point compared to the indicated graph with the two-sided Student’s *t* test.

### Immunoprecipitation and immunoblotting

The cells were treated with the indicated reagents for the indicated durations and lysed with a mixture of lysis buffer (50 mM Tris-HCl, 150 mM NaCl, 1.0% Triton X-100, 1 mM EDTA and 10% glycerol, pH 7.5) and a protease inhibitor cocktail. Cell lysates were incubated with the indicated antibodies overnight and then with agarose G (Invitrogen, 15920-010) for 2 h. The samples were eluted with sample buffer and boiled for 5 min. Normal mouse (Santa Cruz, sc-2025), goat (Santa Cruz, sc-2028) and rabbit IgG (R&D Systems, AB-105-C) were used as controls.

To demonstrate the phosphorylation of TNFR1 in HT-29 and HeLa cells, the treated cells were denatured using SDS to remove protein-protein interaction. Subsequently, lysed with a mixture of lysis buffer (50 mM Tris-HCl, 150 mM NaCl, 1.0% Triton X-100, 1 mM EDTA and 10% glycerol, pH 7.5) and a protease inhibitor cocktail. Cell lysates were incubated with the indicated antibodies overnight and then with agarose G (Invitrogen, 15920-010) for 2 h. The samples were eluted with sample buffer and boiled for 5 min. Normal mouse (Santa Cruz, sc-2025), goat IgG (Santa Cruz, sc-2028) were used as controls.

The following antibodies were used in immunoblotting: anti-EGFR (Cell signalling, #4267S), anti-phospho-EGFR (Tyr1068) (Cell signalling, #3777S), anti-TNFR1 (Santa Cruz, sc-8436), HRP-conjugated anti-FLAG (Sigma Aldrich, A8592), HRP-conjugated anti-HA (Roche, 12013819001), anti-phospho-IKKα/β (Ser176/180) (Cell signalling, #2682), anti-IKKβ (Cell signalling, #2370), anti-IκBα (Cell signalling, #4814), anti-phospho-IκBα (ser539) (Cell signalling, #2859), anti-phospho-p65 (Ser539) (Cell signalling, #3033), anti-p65 (Cell signalling, #8242), anti-TRADD (BD transduction, 610572), anti-TRAF2 (Cell signalling, #4724S), anti-cIAP2 (Cell signalling, #3130), anti-RIPK1 (BD transduction, 610459), anti-phospho-RIPK1 (Ser166) (Cell signalling, #65746S), anti-RIPK3 (Cell signalling, #13526), anti-phospho-RIPK3 (Ser27) (Abcam, ab209384), anti-MLKL (Genetex, GTX107538), anti-phospho-MLKL (Ser358) (Cell signalling, 91689S), anti-caspase-8 (Cell signalling, #9746), anti-PARP (Cell signalling, #9542), anti-β-actin (Sigma Aldrich, A5316-2ML), anti-FADD (Millipore, 05-486), anti-AKT1 (Santa Cruz, sc160340), anti-phospho-AKT1 (Ser473) (Cell signalling, #9271), anti-ERK1/2 (Cell signalling, #4695S), anti-phospho-ERK1/2 (Thr202/Tyr204) (Cell signalling, #4370S), anti-S6K (Cell signalling, #2708), anti-phospho-S6K (Thr389) (Cell signalling, #9205), anti-GST (Santa Cruz, sc-459), anti-His (Santa Cruz, sc8036), anti-phospho tyrosine (Cell signalling, 9411S), anti-mouse RIPK3 (Novus bio, NBP1-77299), anti-phospho-mouse RIPK3 (Ser232) (Abcam, ab195117), anti-mouse MLKL (ABGENT, AP14272b) and anti-phospho-mouse MLKL (Ser345) (Abam, ab196436). The following antibodies were utilised for the immunoprecipitation assay: anti-TNFR1 (R&D Systems, AD225), anti-phospho-EGFR (Tyr1068) (Cell signalling, #3777S), anti-HA (Santa Cruz, sc-805), anti-FLAG (Sigma Aldrich, F7425), anti-FLAG (Sigma Aldrich, F3165), anti-RIPK3 (Cell Signalling Technology, #13526) and anti-caspase-8 (Proteintech, 13423-1-ap).

### Flow cytometry analysis

Cells were treated with the indicated reagents for the indicated times and harvested and stained with Annexin V-FITC (BD Biosciences, 556547) and 7-AAD (eBioscience, 00-69936-50) in Annexin V binding buffer (BD Biosciences, 51-66121E) for 15 min. Post staining, the number of dead cells was measured using a BD Accuri C6 (BD Biosciences). Data are presented as mean ± standard deviation (S.D.), *n* = 3, with ns non-significance, *P < 0.05, **P < 0.01 and ***P < 0.001 at each point compared to the indicated graph with the two-sided Student’s *t* test.

### In vitro kinase assay

6xHis/TNFR1 DD constructs were subcloned into pcDNA3.1 and derived from 293 T cells. 6xHis/TNFR1 DD proteins were pulled-down using Ni-NTA agarose (Qiagen, #30210). Recombinant human EGFR protein (active) (Abcam, Cambridge, UK; ab268518) was procured from Abcam. Each protein was incorporated into TBS-based kinase buffer (Cell signalling, 9802 s) and incorporated 5 μM ATP (Affymetrix, 77241) and 2.5 μCi of [γ -P32]ATP (PerkinElmer, NEG002A100UC). The samples were incubated at 37 °C for 30 min. The reaction was terminated by incorporating sample buffer and incubating at 100 °C for 5 min. The reaction mixture was subjected to SDS-PAGE and radiography.

### Mouse skin inflammation model

The animal experiment was approved by the Institutional Animal Care and Use Committee of Yonsei University (IACUC-202304-1672-02). C57BL/6 female mouse (8-week-old) were purchased from Nara Bioscience. We incorporated 3 μM ASTX660 (Selleck, S8681-5 mg) and 3 μM Emricasan (Sigma Aldrich, SML2227) into 100 μL of 10% Captisol (Selleck, S4592). The mixture was injected into the mice skin. Thereafter, 5 μg/mL EGF (Sigma Aldrich, E9644) was incorporated into PBS (Wellgene, LB201-02) and polyethylene glycol solution (Sigma Aldrich, 76293-100ML-F) (3:7, 200 μL) and topically applied to lesion. Post sacrifice, H&E staining, TUNEL assay and immunohistochemistry (IHC) of skin lesions were performed. The following antibodies were used for IHC: anti-CD3 (Agilent, IR50361-2) and anti-CD11b (Novus Bio, NB110-89474). For histopathological sample analysis, skin sections were scanned at 40× (0.23 μm/pixel) using a NanoZoomer-XR Hamamatsu Photonics (Japan). The evaluation of digitised skin samples involved the assessment of vicissitudes in the epidermal and dermal regions of the skin identifiable on H&E-stained samples. Each ROI was evaluated for the presence of regular epidermis or for any pathological changes in the regular epidermis and dermis. The final computation considered the proportion [%] of the given skin lesions within each sample multiplied by a power score ranging from 0 to 4. A score of 0 was associated with a regular epidermis with no changes in any of the strata. Score 1, thickening of the epidermis represented by any stratum. Score 2, epidermal erosion (partial loss of the epidermis), with intact stratum basale. Score 3, ulcerative loss of the epidermis, including the stratum basale. Score 4, ulceration and dermal/hypodermal fibrosis. The final score (HLS) indicates the severity following the formula: [(0 × % score 0) + (1 × % score 1) + (2 × % score 2) + (3 × % score 3) + (4 × % score 4)]. The lowest possible HLS is ‘0’, which is equal to 100% of normal/regular epidermis in the whole sample and a maximum HLS of ‘400’ is equal to 100% of deep ulceration. NIS-Elements AR 5.30.02 64-bit software was utilised for image visualisation. Murine skin was lysed using lysis buffer (50 mM Tris-HCL, 150 mM NaCl, 1.0% NP40, 0.1% SDS and 0.5% sodium deoxycholate, pH 7.5), protease inhibitor cocktail and homogeniser (Speed Mill, Analytik Jena) and analysed by SDS-PAGE.

### Statistical analysis

Statistical significance was assessed using Student’s *t* test in GraphPad Prism software (Ver. 5.01; La Jolla, CA, USA).

## Results

### The p-EGFR tyrosine kinase domain interacts with the TNFR1 death domain

**Fig. 1:**
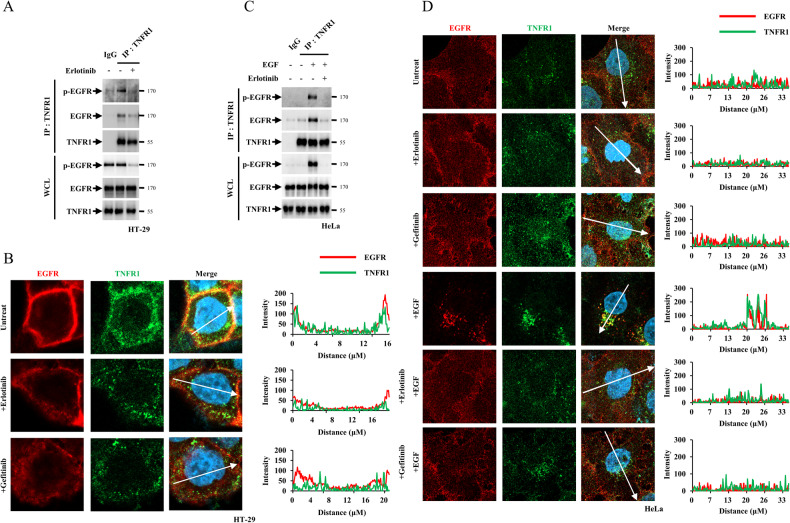
EGFR interacts with TNFR1 through its kinase and death domains. **A** HT-29 cells were treated with 20 μM erlotinib (Erlo) for 30 min. After treatment, the cells were lysed with lysis buffer and incubated with an anti-TNFR1 antibodies. Samples were precipitated by incubation with protein G agarose, followed by immunoblotting using the indicated antibodies. **B** HT-29 cells were treated with 20 μM Erlo or gefitinib (Gefi) for 30 min. After treatment, the cells were fixed and stained with anti-EGFR, TNFR1 antibodies and DAPI. The histogram represents the subcellular intensity of EGFR and TNFR1 in the areas which indicated by white arrows. **C** HeLa cells were treated with 250 ng/ml EGF for 15 min in the presence or absence of 20 μM Erlo for 30 min. After treatment, the cells were lysed with lysis buffer and incubated with an anti-TNFR1 antibodies. Samples were precipitated by incubation with protein G agarose, followed by immunoblotting using the indicated antibodies. **D** HeLa cells were treated with 250 ng/ul EGF for 15 min in the presence or absence of 20 μM Erlo or 20 μM Gefi for 30 min. After treatment, the cells were fixed and stained with anti-EGFR, TNFR1 antibodies DAPI. The histogram represents the subcellular intensity of EGFR and TNFR1 in the areas which indicated by white arrows.

While there is some evidence of crosstalk between EGFR and TNFR1, the detailed mechanistic pathways and implications of this interaction remain unclear and require further study [33–39]. Using HT-29 cells, with constitutively active phosphorylated EGFR (p-EGFR), the interaction between p-EGFR and TNFR1 was determined by immunoprecipitation analyses with anti-EGFR and anti-p-EGFR antibodies (Fig. 1A, Fig. S1A and S1B). When EGFR inhibitors, such as erlotinib or gefitinib, were used to suppress EGFR phosphorylation, the interaction between p-EGFR and TNFR1 reduced (Fig. 1A, Fig. S1A and S1B). Accordingly, immunofluorescence analyses showed that EGFR and TNFR1 were co-localised in HT-29 cells and their interaction ceased upon treatment with erlotinib or gefitinib (Fig. 1B), indicating that EGFR co-localises with TNFR1.

Next, the interaction between EGFR and TNFR1 was tested in HeLa cells, which contain an inert EGFR that can be activated through EGF treatment. The results showed that the two proteins interacted only after EGF treatment, which induced EGFR phosphorylation (Fig. 1C, Fig. S1C and S1D). When EGFR inhibitors were used, the interaction between EGFR and TNFR1 was suppressed, indicating that EGFR phosphorylation might be required for this interaction (Fig. 1C, Fig. S1C and S1D). Corresponding to these results, immunofluorescence analyses showed that EGFR and TNFR1 co-localisation only occurred with EGF treatment and was inhibited by erlotinib and gefitinib treatment (Fig. 1D). The co-localisation of EGFR and TNFR1 was in the cytoplasm and the plasma membrane areas in HeLa cells (Fig. 1D). By treating dynasore, a dynamin inhibitor that prevents endocytosis, in HeLa cells, EGFR and TNFR1 were mainly co-localised on the plasma membrane, indicating that the interaction between the two proteins occurred on the plasma membrane (Fig. S2B). Of note, the co-localisation of EGFR and TNFR in the cytoplasmic area indicates that both receptors might have some effects on each other’s endocytosis, which may affect signalling kinetics. In the future, further studies would be required.

The interaction of the EGFR mutant, with its tyrosine residues substituted to phenylalanine (Y1016/1069/1092/1110/1172/1197F, EGFR 6YF), with TNFR1, was determined to confirm that the p-EGFR engaged actively in its interaction with TNFR1 [40]. Compared to the wild-type EGFR, EGFR 6YF exhibited drastically reduced interaction with TNFR1 (Fig. S3A and S3B). EGFR K745A with impaired kinase activity showed a similar effect as EGFR 6YF in its interaction with TNFR1 (Fig. S3C and S3D). Immunoprecipitation analyses were confirmed by immunofluorescence assays, showing that both EGFR mutants could not co-localise with TNFR1 (Fig. S3E). Wild-type EGFR interacted with TNFR1 but its kinase-defective form significantly lost its ability to bind TNFR1.

To further identify the region responsible for EGFR and TNFR1 interactions, domain mapping analyses were performed using the 293T overexpression system. The generated TNFR1 and EGFR constructs are shown in Fig. S4A and S4C. All TNFR1 constructs interacted with EGFR (Fig. S4B). Similarly, all EGFR constructs generated were able to bind to TNFR1 (Fig. S4D). These results indicate that both the extracellular and intracellular domains of TNFR1 can bind to EGFR and vice versa. However, domain mapping analysis does not accurately reflect the true biological relevance and could only offer the possibility of protein-protein interaction with clues about target sites of the protein. Since the TNFR1 death domain (T-DD) and EGFR tyrosine kinase domain (E-KD) are functionally important for each receptor, we tested whether these two constructs could be associated with one another. Immunoprecipitation data indicated that T-DD was able to interact with E-KD but could not bind to kinase domain-deleted EGFR (E-ΔKD; Fig. S5A). In contrast, E-KD was able to bind to T-DD, but not to death domain-deleted TNFR1 (T-ΔDD; Fig. S5B). This interaction was further confirmed using recombinant GST/EGFR kinase domain (KD) and 6xHis/TNFR1 death domain (DD) recombinant proteins. Ni + -NTA pull-down analysis showed that recombinant EGFR KD and TNFR1 DD could bind to each other (Fig. S5C).

Overall, EGFR phosphorylation appears to be required for its interaction with TNFR1 in cells. When EGF-mediated tyrosine phosphorylation of EGFR is suppressed by inhibitors or point mutations, the interaction between EGFR and TNFR1 is drastically diminished, indicating that the active form of p-EGFR is required for the TNFR1 interaction. Finally, the TNFR1 death domain and the EGFR tyrosine kinase domain, both of which are essential for receptor function, bind to each other.

### p-EGFR negatively regulates TNF-α-induced NF-κB signalling pathways

**Fig. 2:**
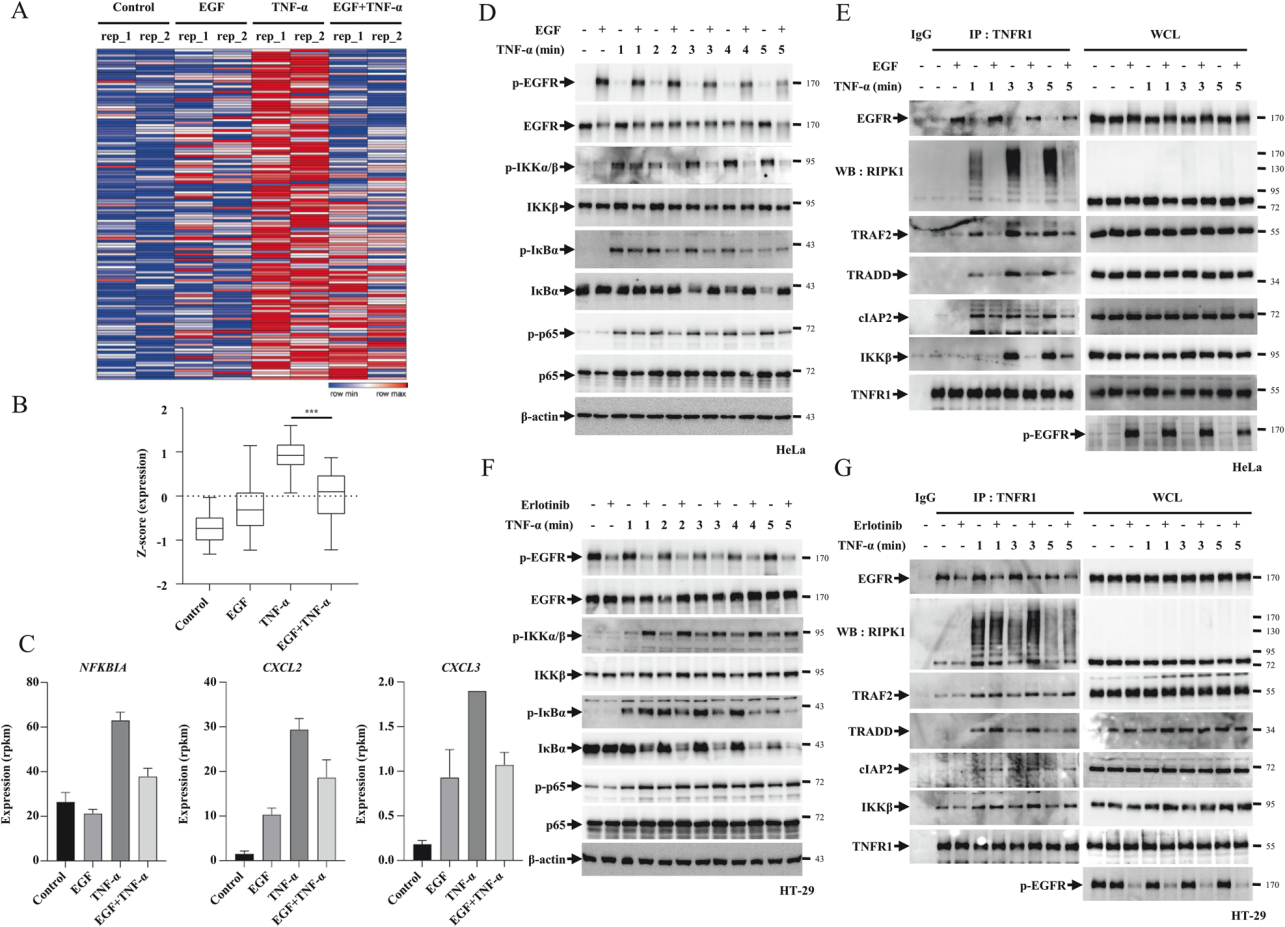
EGFR regulates TNF-α signalling pathways by interacting with TNFR1. **A**–**C** HeLa cells were treated with EGF for 15 min, and then treated with TNF-α. **A** Heatmap showing endogenous genes upregulated by TNF-α. **B** Z-score of (**A**), (**C**) mRNA expression of NFKBIA, CXCL2, and CXCL3. **D** HeLa cells were treated with EGF for 15 min, and then treated with TNF-α for the indicated times, after which the cells were analysed by immunoblotting. **E** HeLa cells were treated with EGF for 15 min and then with TNF-α for the indicated times. After treatment, the cells were lysed with lysis buffer and incubated with an anti-TNFR1 antibodies. Samples were precipitated by incubation with protein G agarose, followed by immunoblotting using the indicated antibodies. **F** HT-29 cells were treated with Erlo for 30 min and then with TNF-α for the indicated times. After treatment, the cells were analysed using immunoblotting. **G** HT-29 cells were treated with Erlo for 30 min and then with TNF-α for the indicated times. After treatment, the cells were lysed with lysis buffer and incubated with an anti-TNFR1 antibodies. Samples were precipitated by incubation with protein G agarose, followed by immunoblotting using the indicated antibodies.

Considering that p-EGFR was able to bind TNFR1, we aimed to examine the effect of EGF on the TNF-α signalling pathway. We exclude the effect of TNF-α on EGFR activation by showing that TNF-α treatment had no effect on the phosphorylation of EGFR upon EGF treatment in HeLa cells and HT-29 cells with constitutively active p-EGFR (Fig. S6A and S6B). We tested the appropriate concentrations of erlotinib and gefitinib required for inducing full suppression of EGFR phosphorylation, at 20 μM (Fig. S7A–S7D). We determined the appropriate concentration of EGF, that is 250 ng/mL, by examining EGF’s ability to fully enhance the phosphorylation of EGFR (Fig. S7E and S7F). To determine whether EGF antagonises transcriptional activation stimulated by TNF-α, we compared the gene expression profiles of HeLa cells treated with EGF or TNF-α via RNA-sequencing (RNA-seq) experiments. The overexpression of the EGFR target gene, EGR1, indicates that EGF treatment stimulated the EGFR1 signalling pathway (Fig. S8A). Gene set enrichment analysis (GSEA) confirmed that treatment with either EGF or TNF-α alone successfully enriched known targets of each pathway (Fig. S8B and S8C). Furthermore, heat map analysis revealed that EGF treatment significantly suppressed the expression of TNF-α inducible targets (Fig. 2A). The Z-score also indicated that EGF co-treatment reduced the expression of genes upregulated by TNF-α compared to the TNF-α only treatment (Fig. 2B). For example, the mRNA levels of NF-κB inhibitor α (NFKBIA), chemokine C-X-C ligand 2(CXCL2) and CXCL3, which are genes upregulated by TNF-α, were decreased in the EGF and TNF-α co-treatment compared to the TNF-α only treatment (Fig. 2C). Real-time polymerase chain reaction (RT-PCR) confirmed that the mRNA levels of these genes also decreased in the EGF co-treatment (Fig. S8D–S8F). In contrast, the expression of EGF-inducible EGR1 was unaffected by TNF-α treatment (Fig. S8A). Taken together, these results indicate that EGF-dependent EGFR activation interferes with the function of the TNF-α signalling pathway.

To further confirm RNA-seq analyses, the TNF-α downstream pathways were detected employing HeLa and Panc-1 cells with inactive EGFR. The data indicated that EGF treatment delayed the TNF-α-stimulated TNFR1 signalling pathways in both cell lines, as indicated by the suppressed phosphorylation of IKK, p65 and IκBα, which lead to the slow degradation of IκBα (Fig. 2D, Fig. S9A). Since the activation of TNFR1 via the ligation of TNF-α mediates the recruitment of complex I, which contains RIPK1 and TRADD, TRAF2, cIAP1/2 and LUBAC that subsequently induce RIPK1 ubiquitination, we tested the effect of EGF on complex I formation and RIPK1 ubiquitination. The treatment of EGF decreased the poly-ubiquitination of RIPK1 and the recruitment of TRADD, TRAF2, cIAP2 and IKKβ, indicating that EGF treatment suppressed complex I formation. It is important to note that complex I formation is essential for the activation of the NF-κB pathway (Fig. 2E). In HT-29, HCT-116 and MDA-MB-231, which have active p-EGFR, the downstream of TNF-α signalling pathways were further analysed in the presence or absence of erlotinib. Erlotinib treatment accelerated the TNF-α signalling pathways stimulated by TNF-α treatment in HT-29 cells. This was evident from the promotion of IKK, p65 and IκBα phosphorylation by erlotinib and the fast degradation of IκBα (Fig. 2F). Likewise, EGFR inhibition by erlotinib in HT-29 cells promoted complex I formation, as indicated by the augmented TRAF2 and TRADD binding with increased RIPK1 poly-ubiquitination, indicating that erlotinib promotes the TNFR1 pathway (Fig. 2G). Similar results were also obtained with HCT-116 and MDA-MB-231 cells, further confirming that EGFR inhibition could accelerate TNF-α signalling pathways (Fig. S10A and S10B).

We next confirmed the negative effect of EGFR on the TNFR1-mediated signalling pathway by generating HT-29 EGFR knockout cells, sgEGFR#1 and #4. As expected, EGFR depletion also promoted the phosphorylation of IKK, p65, IκBα with concomitant fast degradation of IκBα (Fig. S10C and S10D). Overall, the data indicate that the activation of EGFR stifles the NF-κB pathway by suppressing complex I formation. However, the depletion or inhibition of EGFR annihilates the ability of EGFR to negatively regulate the TNFR1-mediated signalling pathway, inducing the promotion of the NF-κB pathway.

### EGFR represses TNF-α-mediated cell death pathways

One of the major effects of TNF-α ligation on TNFR1 is the induction of regulated cell death pathways, such as extrinsic apoptosis or necroptosis [1–4, 41]. When HT-29 cells were treated with TNF-α and birinapant (T/Bi), apoptotic cell death was observed, as detected by annexin V staining and the cleavage of caspase-8 (C8) and PARP (Fig. S11A–S11C). We tested the appropriate concentrations of erlotinib and genfetinib required for inducing optimal T/Bi mediated apoptotic cell death, which were 20 μM (Fig. S16A). Treatment with erlotinib or gefitinib in combination with T/Bi resulted in a more than 20% increase in cell death, along with greater caspase-8 and PARP cleavage, when compared to treatment with T/Bi alone. (Fig. S11A–S11C). The same phenomenon was observed in HCT-116 and MDA-MB-231 cells, confirming the negative effects of active EGFR on apoptosis (Fig. S12). Complex IIb formation, comprising RIPK1, caspase-8 and FADD, is an essential process that induces caspase-8 cleavage and subsequent apoptosis [1–4, 41]. Treatment with EGFR inhibitors increased the rate of Complex IIb formation; this could explain the elevated cell death observed upon treatment with EGFR inhibitors (Fig. S11D). In TNF-α-induced cell death, RIPK1 and TRADD interact with TNFR1, followed by the dissociation of TNFR1, leading to the activation of complex II [1–4, 41]. The data showed that erlotinib promoted interactions between TNFR1, RIPK1 and TRADD under apoptotic conditions (Fig. S11E). We checked whether erlotinib or gefitinib treatment could increase T/Bi-induced apoptosis or enhance the rate of cell death. Kinetics of apoptosis were measured using annexin V in the presence or absence of these inhibitors in HT-29 cells. While the rate of cell death was enhanced under the treatment of inhibitors, the net cell death was unaffected, indicating that EGFR inhibition could affect the kinetics of T/Bi mediated apoptotic cell death rate (Fig. S16B). Complex IIa-mediated apoptosis was tested by treating HT-29 cells with TNF-α and cycloheximide (T/C) in the presence or absence of EGFR inhibitors [1–4, 41]. T/C treatment enhanced apoptosis with more cleavage of C8 and PARP under EGFR inhibitor treatment than the control without the inhibitors (Fig. S13A–S13C). Treatment with EGFR inhibitors promoted complex IIa formation of TRADD, FADD and C8, and interactions between RIPK1, TRADD and TNFR1 (Fig. S13D and S13E).

**Fig. 3:**
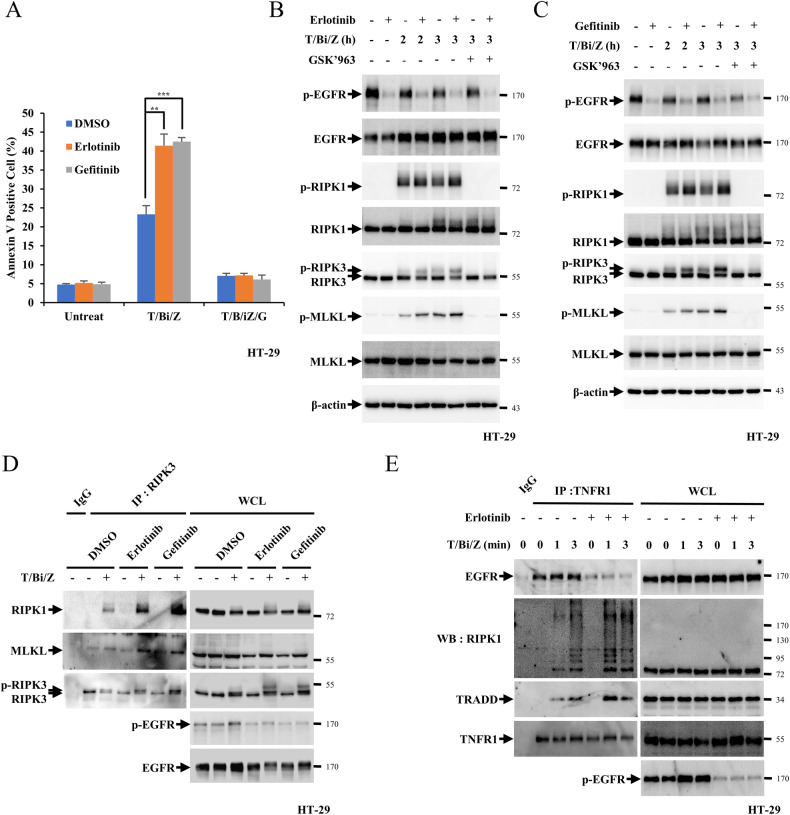
EGFR inhibition promotes TNF-α-induced cell death. **A** HT-29 cells were treated with 30 ng/mL TNFα, 1 μM Birinapant, 30 μM z-VAD-fmk (T/Bi/Z) for 3 h in the presence or absence of 20 μM erlotinib (Erlo) or 20 μM gefitinib (Gefi) for 30 min. After necroptosis was induced, the cells were stained with Annexin V-FITC and 7-AAD for 15 min before analysis using flow cytometry. **B**, **C** p-RIPK1, p-RIPK3, and p-MLKL levels were determined by western blotting. **D** HT-29 cells were treated 20 μM Erlo or 20 μM Gefi for 30 min, and then treated with T/Bi/Z for 3 h. After treatment, cells were lysed with lysis buffer and incubated with an anti-RIPK3 antibodies. Samples were precipitated by incubation with protein G agarose, followed by immunoblotting using the indicated antibodies. **E** HT-29 cells were treated with 20 μM Erlo for 30 min, and then with T/Bi/Z for the indicated times. After treatment, the cells were lysed with lysis buffer and incubated with an anti-TNFR1 antibodies. Samples were precipitated by incubation with protein G agarose, followed by immunoblotting using the indicated antibodies. Data are the mean ± standard deviation (S.D.), *n* = 3, with ns non-significance, *P < 0.05, **P < 0.01, and ***P < 0.001 at each point compared to the indicated graph with the two-sided Student’s *t* test (**A**).

Necroptosis is another pathway that is regulated when extrinsic apoptosis, which is induced by TNF-α, is suppressed. By employing HT-29 cells, the effects of EGFR inhibitors on necroptosis were studied. When HT-29 cells were treated with T/Bi and z-VAD-fmk, a pan-caspase inhibitor (T/Bi/Z), necroptotic cell death was detected by annexin V incorporation and the phosphorylation of RIPK1, RIPK3 and MLKL (Fig. 3A–C). Necroptotic cell death was inhibited upon treatment with GSK’963, a RIPK1 inhibitor, which suppressed RIPK1 phosphorylation, thus inhibiting RIPK3 and MLKL phosphorylation (Fig. 3B, C). We tested the concentrations of erlotinib and gefitinib required for inducing optimal necroptotic cell death, determined at 20 μM (Fig. S16C). Upon treatment with erlotinib or gefitinib, necroptotic cell death was increased by more than 20%, with an increase in the phosphorylation of RIPK1, RIPK3 and MLKL (Fig. 3A–C). Accordingly, necrosome complexes composed of RIPK1, RIPK3 and MLKL, which are essential for inducing necroptotic cell death, were drastically increased upon treatment with EGFR inhibitors (Fig. 3D). Similarly, the interaction between TNFR1, RIPK1 and TRADD was enhanced by erlotinib treatment (Fig. 3E). Kinetics of necroptosis was measured using annexin V in presence or absence of these inhibitors in HT-29 cells to check whether erlotinib or gefitinib treatment could increase TNF-α-induced necroptosis or enhance the rate of TNF-α-induced cell death. While the rate of cell death was enhanced by treatment with the inhibitors, net cell death was unaffected, indicating that EGFR inhibition could affect the kinetics of cell death (Fig. S16D).

**Fig. 4:**
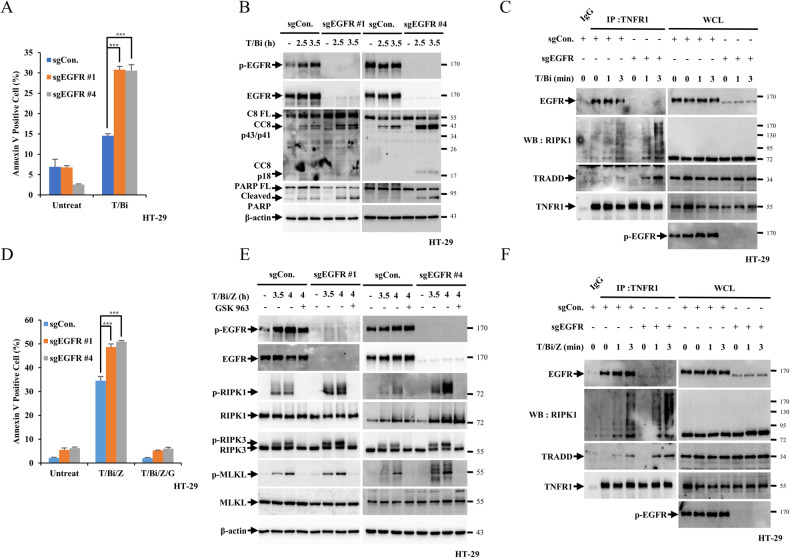
EGFR knockout increases TNF-α-induced cell death. **A** EGFR WT or KO HT-29 cells were treated with T/Bi for 3 h. After treatment, the cells were stained with annexin V-FITC and 7-AAD for 15 min and analysed by flow cytometry. **B** Cleavage of caspase-8 and PARP was determined by western blotting. **C** EGFR WT or KO HT-29 cells were treated with T/Bi for the indicated time periods. After treatment, the cells were lysed with lysis buffer and incubated with an anti-TNFR1 antibodies. Samples were precipitated by incubation with protein G agarose, followed by immunoblotting using the indicated antibodies. **D** EGFR WT or KO HT-29 cells were treated with T/Bi/Z for 4 h. After treatment, the cells were stained with annexin V-FITC and 7-AAD for 15 min and analysed by flow cytometry. **E** p-RIPK1, p-RIPK3, and p-MLKL levels were determined by western blotting. **F** EGFR WT or KO HT-29 cells were treated with T/Bi/Z for the indicated times. After treatment, the cells were lysed with lysis buffer and incubated with an anti-TNFR1 antibodies. Samples were precipitated by incubation with protein G agarose, followed by immunoblotting using the indicated antibodies. Data are the mean ± standard deviation (S.D.), *n* = 3, where non-significance, *P < 0.05, **P < 0.01 and ***P < 0.001 at each point compared to the indicated graph with the two-sided Student’s *t* test (**A**, **D**).

Since EGFR inhibition increased the rate of apoptosis and necroptosis, the effects of EGFR depletion on cell death were investigated. In EGFR-knockout (KO) HT-29 cells, sgEGFR#1 and #4, apoptosis and necroptosis induction were tested. EGFR knockout promoted apoptosis, as shown by increased cell death and cleavage of caspase-8 and PARP upon T/Bi treatment (Fig. 4A, B). Similarly, necroptosis induced by T/Bi/Z was more pronounced in the absence of EGFR, as evident from increased phosphorylation of RIPK1, RIPK3, and MLKL (Fig. 4D, E). Similarly, EGFR knockout increased the interaction between TNFR1, RIPK1 and TRADD in TNF-α-induced apoptotic and necroptotic conditions (Fig. 4C,F). Overall, suppression of EGFR by its inhibitors or knockout accelerated TNF-α-mediated apoptosis and necroptosis, suggesting that EGFR is an anti-apoptotic and anti-necroptotic factor.

### EGFR activation suppresses TNF-α-induced cell death pathways

**Fig. 5:**
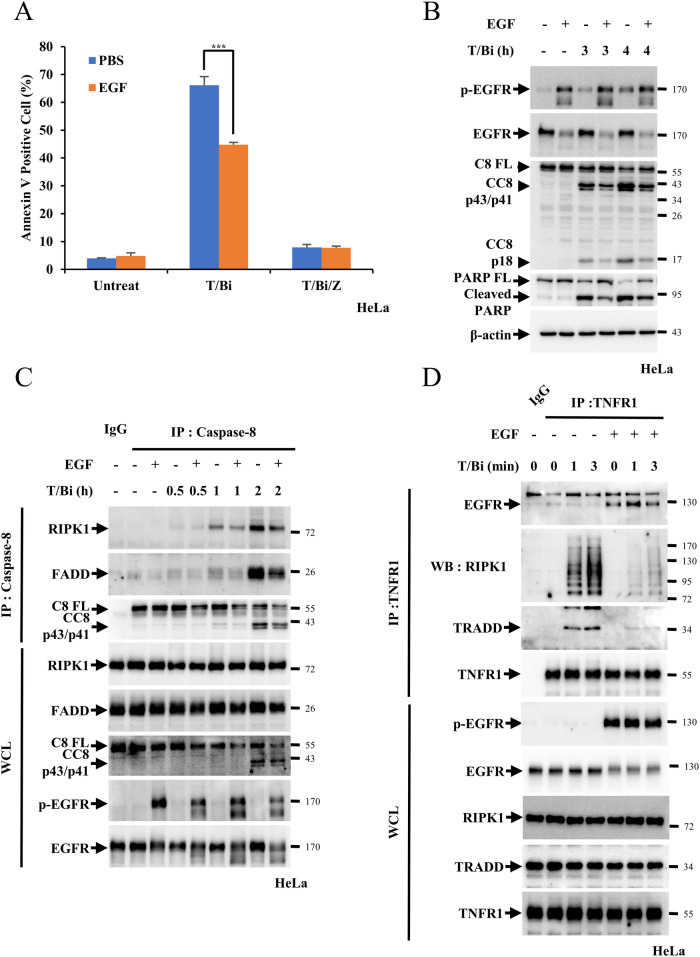
EGFR activation inhibits TNF-α-induced cell death. **A** HeLa cells were treated with 20 ng/mL TNFα and 1 μM Birinapant (T/Bi) for 4 h in the pretreatment or absence of 250 ng/mL EGF for 15 min. After apoptosis was induced, the cells were stained with Annexin V-FITC and 7-AAD for 15 min before analysis using flow cytometry. **B** Cleavage of caspase-8 and PARP was determined by western blotting. **C** HeLa cells were treated with T/Bi for the indicated times in the pretreatment or absence of 250 ng/mL EGF for 15 min. After treatment, cells were lysed with lysis buffer and incubated with an anti-caspase-8 antibodies. Samples were precipitated by incubation with protein G agarose, followed by immunoblotting using the indicated antibodies. **D** HeLa cells were treated with T/Bi for the indicated times in the pretreatment or absence of 250 ng/mL EGF for 15 min. After treatment, the cells were lysed with lysis buffer and incubated with an anti-TNFR1 antibodies. Samples were precipitated by incubation with protein G agarose, followed by immunoblotting using the indicated antibodies. Data are the mean ± standard deviation (S.D.), *n* = 3, with ns non-significance, *P < 0.05, **P < 0.01 and ***P < 0.001 at each point compared to the indicated graph with the two-sided Student’s *t* test (**A**).

EGFR was activated in HeLa and Panc-1 cells following EGF treatment and apoptosis induced by T/Bi was examined to determine the effects of EGFR activation on apoptosis. First, we determined the optimal concentration of EGF by measuring its suppressive effects on cell death, determined at 250 ng/ml (Fig. S16E). T/Bi-induced cell death was suppressed by 20% in both cell lines upon co-treatment with EGF. The complete suppression of cell death by z-VAD-fmk indicated that T/Bi treatment mediated apoptosis (Fig. 5A, Fig. S14A). Supplementing these observations, EGF suppressed the cleavage of caspase-8 and PARP induced by T/Bi in both cell lines (Fig. 5B, Fig. S14B). Further confirming these data, EGF treatment reduced complex IIb formation comprising RIPK1, FADD and caspase-8 induced by T/Bi treatment (Fig. 5C, Fig. S14C) and decreased the interaction between TNFR1, RIPK1 and TRADD in both cell lines (Fig. 5D, Fig. S14D). Kinetics of apoptosis were measured by annexin V in the presence or absence of EGF to investigate whether EGF treatment could decrease the net amount of TNF-α-induced cell death or delay the rate of TNF-α-induced cell death. As in apoptotic pathways, the rate of cell death was reduced under EGF treatment, while the net cell death was unchanged with or without EGF, indicating that EGFR activation could affect the kinetics of cell death (Fig. S16F). Next, we examined complex IIa-mediated apoptosis in HeLa cells by treating T/C in the presence or absence of EGF. Similar to T/Bi-induced apoptosis, EGF treatment decreased T/C-induced apoptosis and the cleavage of caspase-8 and PARP (Fig. S15A and S15B). Complex IIa formation and interaction between TNFR1, RIPK1 and TRADD were reduced by EGF treatment, suggesting that EGFR could regulate complex IIa-mediated cell death and complex IIb-mediated cell death (Fig. S15C and S15D). Lastly, to further confirm the role of EGF in the negative regulation of cell death via EGFR, we generated the EGFR KO HeLa cells, sgEGFR#1 and #4. When the EGFR KO and control cells were treated with T/Bi in the absence of EGF, similar levels of cell death were observed. As expected, treatment of EGF with T/Bi had a negative effect on control cells, but not on HeLa sgEGFR#1 and #4 cells, indicating that EGFR could negatively affect TNF-α-mediated apoptosis (Fig. S17A). These observations were further supported by the fact that treatment with EGF did not suppress caspase-8 or PARP cleavage in EGFR KO cells, whereas it drastically stifled the cleavage of both proteins in control cells (Fig. S17B). In conclusion, the data indicate that the EGF-mediated activation of EGFR could negatively regulate TNF-α-mediated apoptosis.

### EGFR directly suppresses TNF-α signalling by phosphorylating the TNFR1 death domain

The interaction between the EGFR kinase domain and the TNFR1 death domain and the suppression of the TNFR1-mediated pathway by EGFR activation indicates that EGFR might directly suppress TNFR1 activation. The suppression of complex I formation associated with TNFR1 during EGFR activation indicates that TNFR1 is a target of EGFR activation. EGFR WT, EGFR ΔKD and EGFR KD were overexpressed in HeLa cells to investigate whether the EGFR kinase domain is important for regulating TNFR1 signalling by EGFR. EGFR WT and KD with kinase activities suppressed TNF-α-induced apoptosis in HeLa cells, while EGFR ΔKD without kinase activity could not suppress apoptosis (Fig. S18A and S18B). EGFR kinase activity, but not interaction between EGFR and TNFR1, was required for suppressing TNF-α-induced cell death by EGFR. However, we could not exclude the possibility of crosstalk between EGFR downstream effectors and TNFR1; therefore, we treated cells with various inhibitors targeting the PI3K-AKT-mTOR and RAS-RAF-MEK-ERK axes. When HT-29 cell apoptosis was induced by T/Bi in the presence or absence of erlotinib, rapamycin, wortmannin, or AZD6244, which are inhibitors of EGFR, mTOR, PI3K and MEK1/2, respectively, only erlotinib and wortmannin promoted apoptosis, as detected by cell death, caspase-8 and PARP cleavage analyses (Fig. S19A and S19B). It is known that wortmannin can increase TNF-α-mediated apoptosis by suppressing the PI3K-AKT pathway, which inhibits caspase-8 [42, 43]. The other two inhibitors did not stimulate apoptosis mediated by T/Bi treatment, indicating that there may be no regulatory pathways associated with TNFR1, mTOR, or MEK1/2. The effects of these inhibitors on HT-29 necroptotic cell death were also negligible compared to those of erlotinib, indicating that there might be no crosstalk between the PI3K-AKT-mTOR or MEK-ERK axes and the TNFR1 pathway (Fig. S19C and S19D). Notably, there was no change in p-AKT1, p-S6K and p-ERK under treatment with erlotinib, since HT-29 carried BRAF V600E mutation and PI3KCA P449T mutation [44, 45]. Lastly, the decrease in T/Bi-induced apoptosis by EGF treatment in HeLa cells was reversed only by erlotinib treatment and not by other inhibitors, indicating that only EGFR might be a direct regulator of the TNFR1-mediated cell death pathway (Fig. S19E and S19F).

**Fig. 6:**
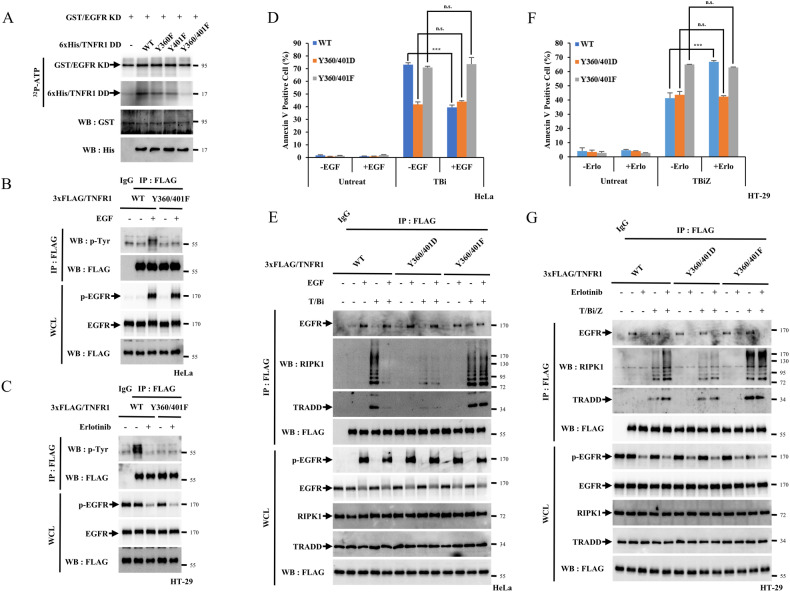
EGFR regulates the signalling pathway by phosphorylating Y360, Y401 in the TNFR1 death domain. **A** An in vitro kinase assay and autoradiography were performed by incubating each recombinant protein and [γ32P]-ATP. The mixtures were determined by western blotting. **B** 3xFLAG/TNFR1 WT and Y360/401F reconstituted HeLa cells were treated with 250 ng/mL EGF for 15 min. After treatment, the HeLa cells were denatured using SDS and lysed with lysis buffer and incubated with an anti-FLAG antibodies. Samples were precpitatbed by incubation with protein G agarose, followed by immunoblotting using the indicated antibodies. **C** 3xFLAG/TNFR1 WT and Y360/401F reconstituted HT-29 cells were treated with 20 μM Erlo for 30 min. After treatment, the HT-29 cells were denatured using SDS and lysed with lysis buffer and incubated with an anti-FLAG antibodies. Samples were precpitated by incubation with protein G agarose, followed by immunoblotting using the indicated antibodies. **D** 3xFLAG/TNFR1 WT, Y360/401D, and Y360/401F reconstituted HeLa cells were treated with 20 ng/mL TNF-α, and 1 μM Birinapant (T/Bi) for 3 h in the pretreatment or absence of 250 ng/mL EGF for 15 min. After apoptosis was induced, the cells were stained with Annexin V-FITC and 7-AAD for 15 min before analysis using flow cytometry. **E** 3xFLAG/TNFR1 WT, Y360/401D and Y360/401F reconstituted HeLa cells were treated with T/Bi in the pretreatment or absence of 250 ng/mL EGF for 15 min. After treatment, the cells were lysed with lysis buffer and incubated with an anti-FLAG antibodies. Samples were precpitatbed by incubation with protein G agarose, followed by immunoblotting using the indicated antibodies. **F** 3xFLAG/TNFR1 WT, Y360/401D and Y360/401F reconstituted HT-29 cells were treated with T/Bi/Z for 3 h. After treatment, the cells were stained with annexin V-FITC and 7-AAD for 15 min and analysed using flow cytometry. **G** 3xFLAG/TNFR1 WT, Y360/401D and Y360/401F reconstituted HT-29 cells were treated with T/Bi/Z in the pretreatment or absence of 20 μM Erlo for 30 min. After treatment, the cells were lysed with lysis buffer and incubated with an anti-FLAG antibodies. Samples were precipitated by incubation with protein G agarose, followed by immunoblotting using the indicated antibodies. Data are the mean ± standard deviation (S.D.), *n* = 3, with ns non-significance, *P < 0.05, **P < 0.01 and ***P < 0.001 at each point compared to the indicated graph with the two-sided Student’s *t* test (**D**, **F**).

Once the possibility of a regulatory effect of active EGFR on TNFR1 was established, we investigated whether the EGFR tyrosine kinase could directly mediate TNFR1 phosphorylation. Because the previous immunoprecipitation data showed that the EGFR kinase domain was able to bind to the TNFR1 death domain, we employed active GST/EGFR KD and 6xHis/TNFR1 DD recombinant proteins to conduct in vitro phosphorylation analyses. The results showed that EGFR KD induced phosphorylation of the TNFR1 DD (Fig. S20A). We confirmed the ability of p-EGFR in inducing TNFR1 phosphorylation in cells. We treated HeLa cells with EGF in the presence or absence of erlotinib or gefitinib followed by immunoprecipitation analysis using anti-TNFR1 antibodies and western blotting using anti-phospho-tyrosine antibodies. EGF treatment increased TNFR1 phosphorylation, while co-treatment with an EGFR inhibitor drastically suppressed it (Fig. S20B and S20C). In HT-29 cells, phosphorylation of TNFR1 was detected under normal conditions but decreased after treatment with EGFR inhibitors (Fig. S20D). As the TNFR1 DD contains two tyrosine residues at positions 360 and 401, we hypothesised that these tyrosine residues might be targeted by EGFR KD and subsequently generated mutants, Y360F, Y401F and the double mutant, Y360/401F. The in vitro phosphorylation analyses indicated that only the Y360/401F mutation was not phosphorylated by EGFR KD (Fig. 6A). HeLa or HT-29 cells with TNFR1 WT depleted were infected with viruses with TNFR1 WT or Y360/401F to investigate the phosphorylation of TNFR1 at Y360 and Y401 in cells. Along with in vitro data, TNFR1 WT was phosphorylated by EGF treatment, while TNFR1 Y360/401F was not in HeLa cells (Fig. 6B). In HT-29 cells, TNFR1 WT phosphorylation was detected under normal condition while TNFR1 Y360/401F phosphorylation was not. Treatment with erlotinib drastically suppressed TNFR1 WT phosphorylation but had no effect on TNFR1 Y360/401F (Fig. 6C). Data from recombinants and cells indicated that EGFR could phosphorylate TNFR at Y360 and Y401. We further constructed the mimetic mutants Y360D, Y401D and Y360/401D to mimic the effect of TNFR1 phosphorylation by active EGFR. These mutants did not lose their ability to interact with wild-type (WT) TNFR1, indicating that the mutant does not interfere with TNFR1 trimerization (Fig. S21A). However, the interaction with RIPK1 or TRADD was decreased in the mutants compared to TNFR1 WT, indicating that these sites may be associated with downstream effectors (Fig. S21B and S21C).

To investigate the effect of the mutants on cellular signalling, HeLa cells depleted of TNFR1 were reconstituted with TNFR1 WT, Y360/401D, or Y360/401F (Fig. S22A). Y360/401D is a mimetic mutant of TNFR1 phosphorylated by EGFR, while the Y360/401F mutant TNFR1 is supposedly unaffected by EGFR stimulation. When T/Bi-mediated apoptosis was measured in reconstituted HeLa cells, Y360/401F induced cell death comparable to that in TNFR1 WT in the absence of EGF. Under the same conditions, the expression of Y360/401D lead to the suppression of apoptotic cell death by 30%, indicating that this mutant is less effective in inducing apoptosis. In the presence of EGF, there was a 30% reduction in TNFR1 WT’s ability to induce apoptosis, whereas Y360/401F was not affected by the presence of EGF (Fig. 6D). Confirming these findings, the presence of EGF was only able to suppress the cleavage of caspase-8 and PARP, as well as the interaction between TNFR1, RIPK1 and TRADD when TNFR1 WT was expressed, whereas it had no effect on the expression of Y360/401F. However, when Y360/401D was expressed, the cleavage of caspase-8 and PARP and their interaction were inhibited in the presence or absence of EGF, as expected (Fig S23A and Fig. 6E).

To confirm the effects of these tyrosine residues, TNFR1 knockdown HT-29 cells were reconstituted with TNFR1 WT, Y360/401D, Y360/401 F (Fig. S22C). In the absence of erlotinib, T/Bi/Z-induced necroptosis or phosphorylation of RIPK1, RIPK3 and MLKL were comparable to that in the reconstituted TNFR1 WT when Y360/401D was expressed. The addition of erlotinib promoted cell death and was affected by T/Bi/Z treatment, only under the expression of WT but not Y360/401D, as expected (Fig. 6F and Fig S23B). Unlike Y360/401D, the expression of Y360/401 F resulted in increased T/Bi/Z-induced necroptosis and phosphorylation of RIPK1, RIPK3 and MLKL, with or without erlotinib, when compared to the reconstituted TNFR1 WT (Fig. 6F, Fig S23B). Furthermore, cell lines with the reconstitution of Y360/401D demonstrated decreased complex formation of TNFR1, RIPK1 and TRADD, along with decreased RIPK1 polyubiquitination, in the presence or absence of erlotinib when compared to the WT (Fig. 6G). In contrast, cell lines with reconstitution of Y360/401F induced more TNFR1, RIPK1 and TRADD complex formation and promoted RIPK1 polyubiquitination, regardless of erlotinib, compared to the WT (Fig. 6G).

Finally, the apoptotic effects of reconstituted WT TNFR1, Y360/401D, or Y360/401F in HT-29 cells were tested. The results showed that Y360/401D expression lead to similar levels of apoptotic cell death and cleavage of caspase-8 and PARP induced by T/Bi compared with the WT, without erlotinib. However, unlike the WT, T/Bi-induced cell death in the presence of Y360/401D was not increased in the presence of erlotinib. The expression of Y360/401F resulted in an increase in apoptosis and cleavage of caspase-8 and PARP induced by T/Bi, regardless of erlotinib, compared to the WT (Fig. S24). Overall, the results suggest that active EGFR phosphorylates two tyrosines, 360 and 401, on the death domain of TNFR1, leading to the suppression of TNFR1-RIPK1-TRADD complexes. The inhibition of these complexes leads to a decrease in apoptosis and necroptosis, suggesting that EGFR negatively regulates TNFR1 downstream pathways.

### EGF treatment mitigates TNF-α-induced necroptotic skin inflammation

**Fig. 7:**
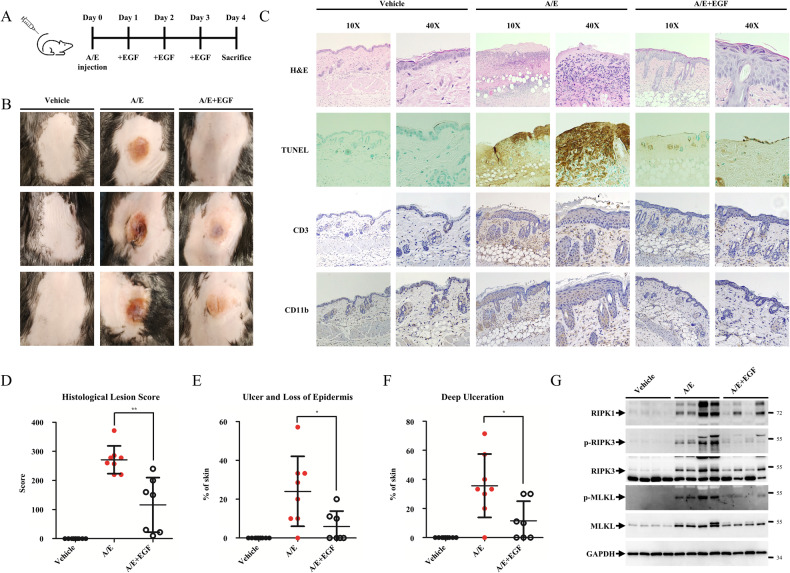
EGF treatment mitigates necroptosis-driven skin inflammation. **A** Scheme of the skin inflammation model. **B** Representative images of skin lesions from mice 4 days after injection with vehicle control (*n* = 9), ASTX660 and Emricasan (A/E) (*n* = 7) or EGF application with ASTX660 and Emricasan (EGF + A/E) (*n* = 7) (**C**) Representative images of lesions treated as in (**A**) and stained with H&E, performing a TUNEL assay and immunostained with anti-CD3 and anti-CD11b antibodies. **D** Histological multivariate lesion score (HLS) of mice treated as described in (**A**). **E** Percentage contribution of ulcers and loss of epidermis in each mouse. **F** Percentage contribution of deep ulceration in each mouse. **G** The skins were lysed using lysis buffer and a homogeniser, followed by immunoblotting analysis using the indicated antibodies. Data are the mean ± standard deviation (S.D.), vehicle (*n* = 9), A/E (*n* = 7), A/E + EGF (*n* = 7) with ns non-significance, *P < 0.05, **P < 0.01 and ***P < 0.001 at each point compared to the indicated graph with the two-sided Student’s *t* test (**D**, **E**, **F**).

Skin is a tissue in which failure of the regulation of the TNFR1 signalling pathway by TNF-α could induce inflammatory diseases such as psoriasis and atopic dermatitis [46–49]. In previous studies, researchers have shown that subcutaneous injections of the cIAP and caspase inhibitors, ASTX660 and emricasan, respectively, induces TNF-α and RIPK1-dependent necroptotic skin inflammation [50, 51]. We employed this model system to determine whether EGF has therapeutic effects on TNF-α-induced skin inflammation. To induce necroptosis in mouse skin, ASTX660 and emricasan (A/E) were injected into the dorsal skin. After 24 h, EGF was applied to the same area for the next 3 days to investigate the effect of EGF on skin inflammation (Fig. 7A). While A/E injection induced severe skin inflammation after 4 days, the application of EGF significantly suppressed skin inflammation (Fig. 7B).

The skin lesions of mice injected with A/E displayed abundant macrophage recruitment, whereas these phenomena were stifled by EGF treatment (Fig. 7C, H&E panels). When the degree of necroptosis was observed using terminal deoxynucleotidyl transferase dUTP nick-end labelling (TUNEL), a thick and strong TUNEL-positive area was observed on the skin of mice injected with A/E. In contrast, EGF treatment drastically reduced the lesion areas of skin stained with TUNEL (Fig. 7C, TUNEL panels). Likewise, while the recruitment of T cells and macrophages was increased in the areas treated with A/E, EGF treatment decreased the immune response (Fig. 7C, CD3 & CD11b panels). The A/E group achieved high histological lesion scores with a mean of 241, but EGF treatment mitigated 42% of the histological lesion score (Fig. 7D). Thickening of the epidermis and epidermal erosion did not differ, but ulceration, loss of the epidermis and deep ulceration, all indicators of severe skin inflammation, decreased in the A/E plus EGF group compared to the A/E group (Fig. 7E, F and Fig. S25). We further confirmed necroptotic inflammation and cell death by detecting necroptotic markers, such as phosphorylated RIPK3 and MLKL. Upon A/E injection, there was an increase in phosphorylated RIPK3 and MLKL, which was suppressed by EGF treatment (Fig. 7G). Interestingly, there was also an increase in the levels of RIPK1, RIPK3 and MLKL after A/E injection compared to those in the control or EGF-treated groups (Fig. 7G). Overall, these data suggest that EGF treatment mitigates TNF-α-induced necroptotic skin inflammation and is, thus, a plausible therapeutic strategy.

## Discussion

While crosstalk between the EGFR and TNFR1 signalling pathways has been reported, the detailed mechanisms and associated physiological implications remain unclear and controversial. Several studies have suggested that active EGFR promotes the TNFR1-related pathway. For example, EGF-mediated EGFR signalling could activate the NF-κB signalling pathway through SOS-GRB2-facilitated IKK complex activation [52, 53]. In the cell death pathway, EGF treatment induces apoptosis via AKT1 inhibition in EGFR overexpressed dominant-negative Ras mutant cell lines, or via phosphorylation of STAT3 in glioblastoma; however, the mechanisms for these pathways are not clear [54, 55]. In addition, EGFRvIII is known to activate the NF-κB signalling pathway via RIPK1-ubiquitination, while WT EGFR seems to suppress this process leading to RIPK1-dependent apoptosis in glioblastoma [56]. These reports indicate that TNFR1 is not a direct target of EGFR. Another study indicated that EGFR interacts with and promotes the TNFR1 pathway, which increases lung inflammation by promoting RIPK3-dependent necroptosis [37]. In this study, the effects of EGF on the TNFR1 and EGFR interactions were not tested. However, the negative regulatory roles of EGFR in TNFR1-related pathways have been suggested. EGFR inhibition promotes the secretion of TNF-α leading to JNK phosphorylation [38]. Furthermore, EGFR inhibition increases TNF-α-induced apoptosis via p38 MAPK, the mechanism for which is not clearly described [36]. TNFR1 was also reported to function as an EGFR regulator. It induces the phosphorylation of the tyrosines of EGFR, leading to the activation of the EGFR signalling pathway in a TAK1- and p38 α-dependent manner [57–59]. In contrast, it has been suggested that TNFR1 inhibits EGFR activation via the death domain of TNFR1 dependent manner, the mechanism of which is unclear [33]. Overall, although the association between EGFR and TNFR1 or their downstream effectors has been described, the detailed regulatory and physiological mechanisms connecting these two essential receptors are still not understood.

In this study, we identified TNFR1 as a target protein of EGFR tyrosine kinase. EGF ligation, followed by EGFR phosphorylation, induces an interaction between EGFR and TNFR1. In the absence of EGFR activation, no interaction was observed between the two proteins. This association leads to the suppression of TNFR1-mediated pathways, including the NF-κB pathway, complex II-dependent apoptosis and necroptosis. These phenomena were further elucidated by RNA sequencing and the generation of the EGFR KO HeLa cell line. RNA sequencing analyses using HeLa cells treated with EGF or EGF and TNF-α co-treatment indicated that EGF treatment significantly suppressed the transcription of mRNA upregulated by TNFα treatment such as NFKBIA, CXCL2, CXCL3. When EGFR was knocked out in HeLa cells, the suppression of TNFR1-mediated apoptosis by EGF was completely eliminated, indicating that EGFR may be a suppressor of TNFR1. The interaction between EGFR and TNFR1 was further confirmed using the EGFR inhibitors, erlotinib and gefitinib. The presence of either of these inhibitors inhibited the EGF-induced interaction between EGFR and TNFR1, as confirmed by immunoprecipitation and immunofluorescence analyses. These data indicate that cell lines with inactive EGFR could inhibit TNFR1 upon EGF treatment, which induces EGFR activation. To further validate the association between the two receptors, the cell lines, HT-29, HCT-116 and MDA-MB-231, which express active EGFR, were used. In contrast to cell lines with inert EGFR, TNFR1 and EGFR in these cell lines displayed strong interactions with each other and were significantly dissociated by inhibitor treatment. Corresponding to these results, the treatment of inhibitors also accelerated T/Bi-mediated extrinsic apoptosis in these cell lines. Using HT-29 the effects of the EGFR inhibitors on necroptosis were confirmed. The results indicated that erlotinib or gefitinib treatment promoted TNF-α, birinapant and z-VAD-fmk-mediated necroptosis, which were analysed using RIPK1, RIPK3 and MLKL phosphorylation and complex formation. HT-29 EGFR knockout cell lines also exhibited higher sensitivity to necroptotic shock, indicating a suppressive role of EGFR in necroptosis.

We also attempted to study the mechanistic rational for EGFR-mediated suppression of TNFR1-dependent signalling pathways including NF-κB, apoptosis and necroptosis. The treatment of EGFR downstream inhibitors targeting PI3K, mTORC1 and MEK1/2 had minor effects on TNF-α dependent cell death, which indicate the possibility of direct association between EGFR and TNFR1. Direct interaction between the tyrosine kinase domain of EGFR and the death domain of TNFR1 using recombinant proteins further supported this hypothesis. Indeed, active recombinant EGFR tyrosine kinase was able to induce the phosphorylation of the TNFR1 death domain, which contains two tyrosines, 360 and 401. When point mutations were introduced in these two tyrosines, EGFR tyrosine kinase was no longer able to induce the phosphorylation of the TNFR1 death domain. The functional importance of these tyrosines was further confirmed by reconstitution analyses, where the phosphomimetic mutant, Y360/401D, resulted in less TNF-α-dependent cell death compared to TNFR1 WT in HeLa cells. As expected, the addition of EGF did not affect the function of this mutant. When Y360/401F, the phosphorylation-defective mutant, was reconstituted, it exhibited similar levels of cell death as the WT, but significantly enhanced cell death compared to Y360/401D in the absence of EGF. These phenomena were rescued by EGF treatment during the expression of WT but not of Y360/401F, suggesting that phosphorylation processes at tyrosine 360 and 401 are necessary for the suppression of TNFR1. Correspondingly, in HT-29 cells with active EGFR, the reconstitution of Y360/401D and TNFR1 WT had similar apoptotic and necroptotic cell death effects, whereas Y360/401F lead to increased cell death. Interestingly, the addition of erlotinib did not promote cell death in HT-29 cells expressing Y360/401D, indicating that a mimetic phosphorylated form of TNFR1 is not affected by an EGFR inhibitor. In addition, mimics of TNFR1 phosphorylation or EGFR activation led to decrease of interactions between TNFR1, RIPK1 and TRADD. These data indicate that the phosphorylation of Tyr 360 and 401 in the TNFR death domain does indeed suppress cell death.

The regulation of the TNFR1 signalling pathway by TNF-α is considered clinically significant because failure to modulate this mechanism could lead to serious diseases, such as inflammatory bowel diseases, rheumatoid arthritis, psoriatic arthritis, psoriasis, juvenile idiopathic arthritis, ankylosing spondylitis, Crohn’s disease, degenerative diseases and ulcerative colitis [3, 48]. Thus, investigations on TNFR1 checkpoints have been broadly carried out to block TNF-α related diseases. For example, monoclonal antibodies, domain antibodies, nanobodies and TNF mutein are currently developed to target TNFR1 or 2. [46–49]. However, the short- and long-term side effects of targeting TNFR1 hinder efficacious drug development [46–49]. Among various diseases caused by TNFR1 and TNF-α, psoriasis and atopic dermatitis are representative inflammatory diseases developed on the skin [46–49]. Since the treatment of mouse skin with IAP and caspase inhibitors can induce TNF-α-dependent skin inflammatory diseases, we further analysed the effect of EGF on this model system [50, 51]. Dorsal treatment with EGF decreases necroptosis in the skin by reducing the phosphorylation of RIPK3 and MLKL and suppressing skin lesions and immune cell recruitment. The findings of our study suggest that treatments could be developed for TNFR1-related immune diseases by employing EGF or EGFR agonists in a future clinically valid trial. TNF-α is a mediator exerting therapeutic effects against cancer. Recently, antibody-TNF-α fusion proteins have been developed to allow local TNF-α effect on cancer cells. These tests show substantial efficacy in animal cancer models and a small group of patients with glioblastoma [60–62]. Since EGFR mutations are present in glioblastomas, anti-EGFR therapy with chemicals and antibodies is a potential treatment option. Conjugating TNF-α with anti-EGFR antibodies, such as cetuximab, may be an effective immunocytokine therapy for tumours.

## Supplementary information

Supplementary Figure and Table legends

Table S1

Table S2

original blot

## Data Availability

The corresponding author can provide the datasets used in this study upon reasonable request.
