# Correction: EGFR inhibits TNF-α-mediated pathway by phosphorylating TNFR1 at tyrosine 360 and 401

**DOI:** 10.1038/s41418-024-01425-z

**Published:** 2024-12-03

**Authors:** Young Woo Nam, June-Ha Shin, Seongmi Kim, Chi Hyun Hwang, Choong-Sil Lee, Gyuho Hwang, Hwa-Ryeon Kim, Jae-Seok Roe, Jaewhan Song

**Affiliations:** Department of Biochemistry, College of Life Science and Technology, Institute for Bio-medical Convergence Science and Technology, Yonsei University, Seoul, Republic of Korea

**Keywords:** Molecular biology, Cell biology

Correction to: *Cell Death & Differentiation* 10.1038/s41418-024-01316-3, published online 24 May 2024

In this article, the affiliation of Jaewhan Song “Department of Biochemistry, College of Life Science and Biotechnology, Yonsei University, Seoul, Korea” has been updated to “Department of Biochemistry, College of Life Science and Technology, Institute for Bio-medical Convergence Science and Technology, Yonsei University, Seoul, Republic of Korea”.

The original article has been updated.

