# Supplementary material for: EGFR inhibits TNF-α-mediated pathway by phosphorylating TNFR1 at tyrosine 360 and 401
Source: Cell Death Differ. 2024 May 24;31(10):1318–32. doi: 10.1038/s41418-024-01316-3 (PMC11445491; doi:10.1038/s41418-024-01316-3)
Supplement: Supplementary file 1 — Supplementary Figure and Table legends [file 41418_2024_1316_MOESM1_ESM.pdf]

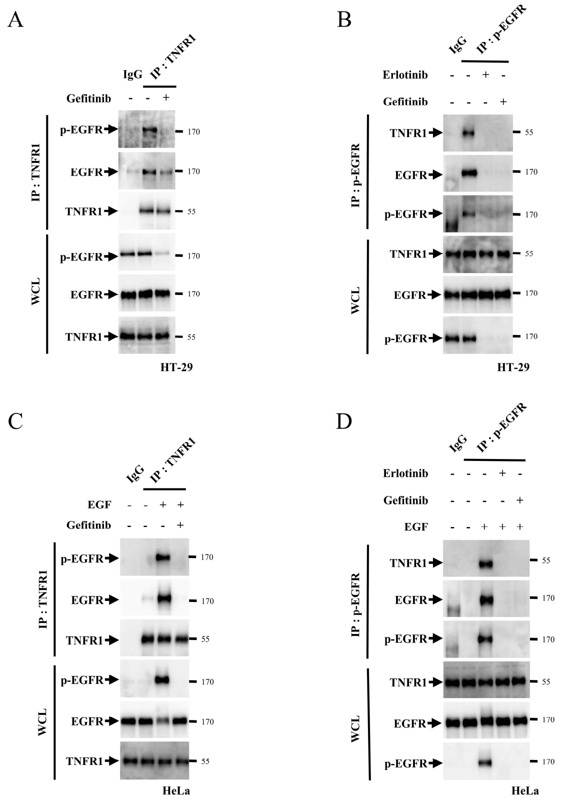

Supplementary Figure 1. Erlotinib and gefitinib decreases the interaction between EGFR and TNFR1.

(a) HT-29 cells were treated with 20 μM gefitinib (Gefi) for 30 min. After treatment, the cells were lysed with lysis buffer and incubated with anti-TNFR1 antibodies. Samples were precipitated by incubating with protein G agarose, followed by immunoblotting analysis using the indicated antibodies. (b) HT-29 cells were treated with 20 μM erlotinib (Erlo) or 20 μM gefitinib (Gefi) for 30 min. After treatment, the cells were lysed with lysis buffer and incubated with anti-p-EGFR antibodies. Samples were precipitated by incubating with protein G agarose, followed by immunoblotting analysis using the indicated antibodies. (c) HeLa cells were treated with 250 ng/mL EGF for 15 min in the pretreatment or absence of 20 μM Gefi for 30 min. After treatment, the cells were lysed with lysis buffer and incubated with an anti-TNFR1 antibodies. Samples were precipitated by incubating with protein G agarose, followed by immunoblotting analysis using the indicated antibodies. (d) HeLa cells were treated with 250 ng/mL EGF for 15 min in the pretreatment or absence of 20 μM Erlo or 20 μM Gefi for 30 min. After treatment, the cells were lysed with lysis buffer and incubated with an anti-p-EGFR antibodies. Samples were precipitated by incubating with protein G agarose, followed by immunoblotting analysis using the indicated antibodies.

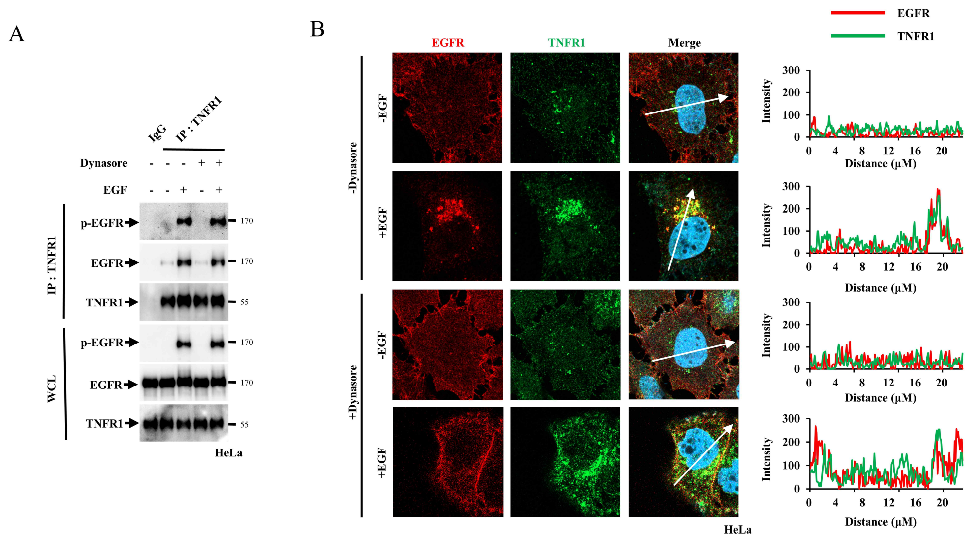

Supplementary Figure 2. p-EGFR interacts with TNFR1 under dynasore treatment condition.

(a) HeLa cells were treated with 250 ng/mL EGF for 15 min in the pretreatment or absence of 80 μM Dynasore for 4 h. After treatment, the cells were lysed with lysis buffer and incubated with an anti-TNFR1 antibodies. Samples were precipitated by incubating with protein G agarose, followed by immunoblotting analysis using the indicated antibodies. (b) HeLa cells were treated with 250 ng/mL EGF for 15 min in the pretreatment or absence of 80 μM Dynasore for 4 h. After treatment, the cells were fixed and stained with anti-EGFR, TNFR1 antibodies and DAPI. The histogram represents the subcellular intensity of EGFR and TNFR1 in the areas which indicated by white arrows.

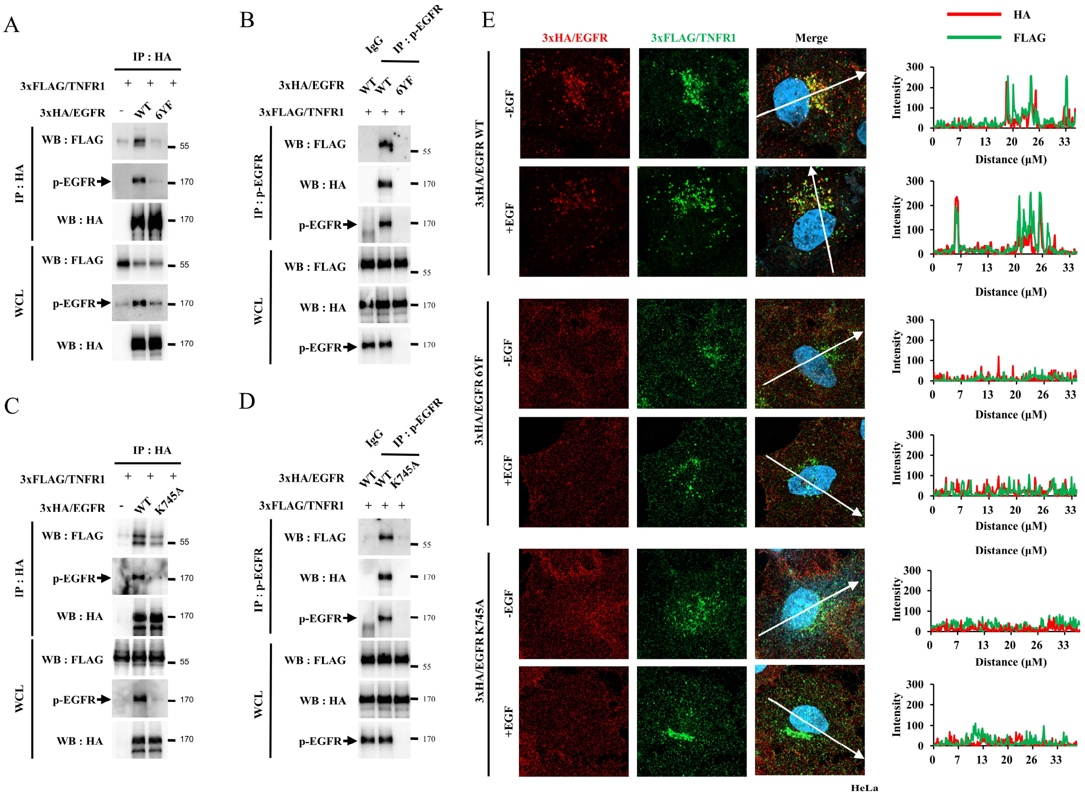

Supplementary Figure 3. EGFR kinase defective-mutation decreases the interaction between EGFR and TNFR1.

(a-d) 293T cells were transfected with the indicated plasmids. After transfection, cells were lysed with lysis buffer and incubated with the indicated antibodies. Samples were precipitated by incubation with protein G agarose, followed by immunoblotting using the indicated antibodies. (e) HeLa cells were transfected using pCS5/3xFLAG TNFR1, pCS5/3xHA EGFR WT, 6YF, and K745A plasmids. After transfection, the cells were fixed and stained using anti-HA and FLAG antibodies and DAPI. The histogram represents the subcellular intensity of 3xHA/EGFR and 3xFLAG/TNFR1 in the areas which indicated by white arrows.

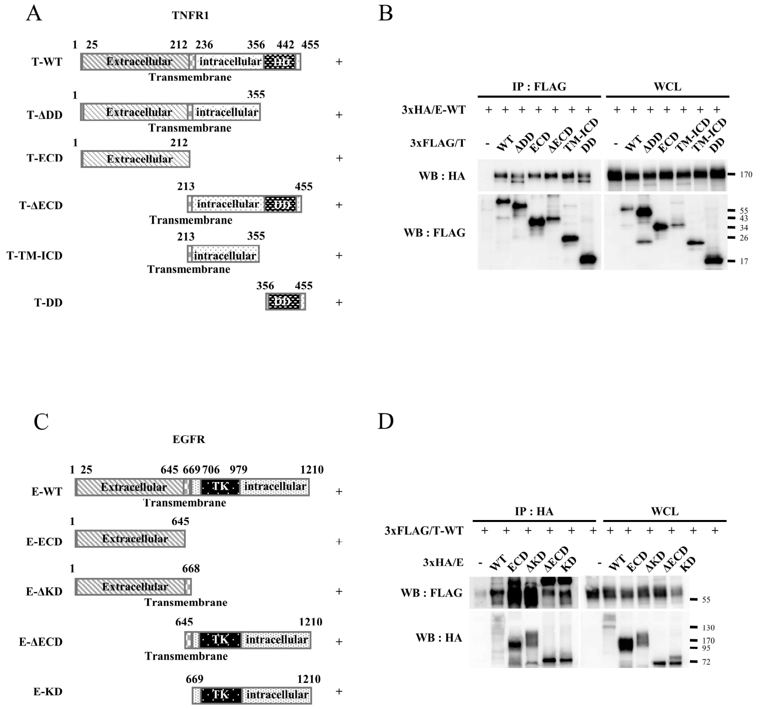

Supplementary Figure 4. EGFR interacts with TNFR1.

(a) Mapping of the binding affinity between each TNFR1 domain and EGFR. (b) 293T cells were transfected using the indicated plasmids. After transfection, the cells were lysed with lysis buffer and incubated with anti-FLAG antibodies. Samples were precipitated by incubating with protein G agarose, followed by immunoblotting analysis using the indicated antibodies. (c) Mapping of the binding affinity between each EGFR domain and TNFR1. (d) 293T cells were transfected using the indicated plasmid. After transfection, the cells were lysed with lysis buffer and incubated with anti-HA antibodies. Samples were precipitated by incubating with protein G agarose, followed by immunoblotting analysis using the indicated antibodies.

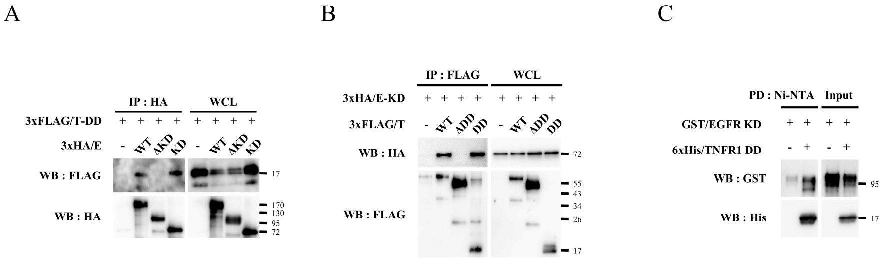

Supplementary Figure 5. EGFR kinase domain interacts with TNFR1 death domain.

(a) 293T cells were transfected using the indicated plasmids. After transfection, the cells were lysed with lysis buffer and incubated with anti-HA antibodies. Samples were precipitated by incubating with protein G agarose, followed by immunoblotting analysis using the indicated antibodies. (b) 293T cells were transfected using the indicated plasmids. After transfection, the cells were lysed with lysis buffer and incubated with the anti-FLAG antibodies. Samples were precipitated by incubating with protein G agarose, followed by immunoblotting analysis using the indicated antibodies. (c) Recombinant protein GST/EGFR KD and 6xHis/TNFR1 DD were mixed in lysis buffer and incubated. Samples were precipitated by incubating with Ni-NTA agarose, followed by immunoblotting analysis using the indicated antibodies.

A B

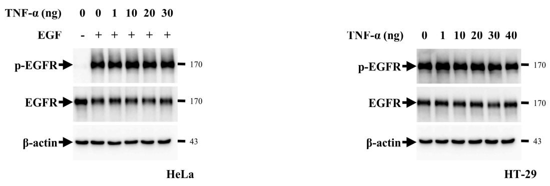

Supplementary Figure 6. TNF-α treatment does not affects EGFR activation.

(a) HeLa cells were treated with the TNF-α at the indicated concentrations for 5 min and treated with 250 ng/mL EGF for 15 min. After treatment, the cells were analysed using immunoblotting. (b) HT-29 cells were treated with the TNF-α at the indicated concentrations for 5 min. After treatment, the cells were analysed using immunoblotting.

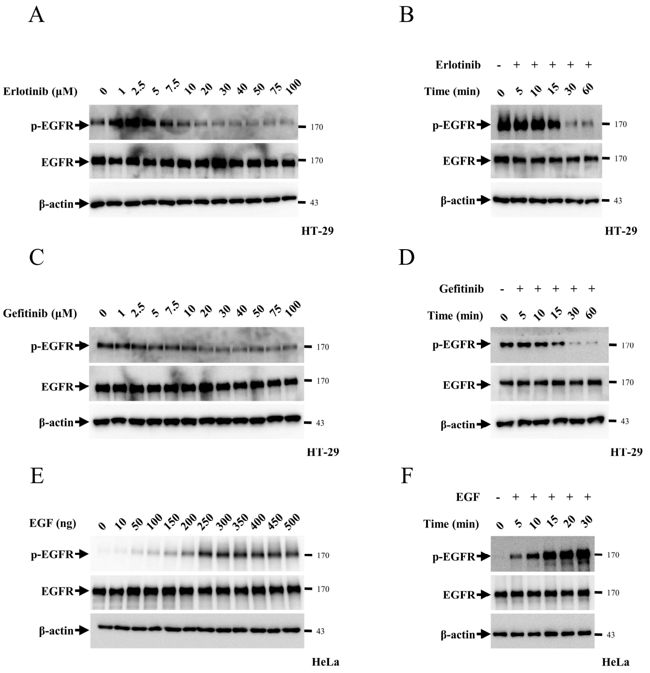

Supplementary Figure 7. EGF activates EGFR in HeLa cell lines, and EGFR inhibitor suppresses EGFR in HT-29 cell lines.

(a) HT-29 cells were treated with Erlo at the indicated concentrations for 30 min. After treatment, the cells were analysed using immunoblotting. (b) HT-29 cells were treated with 20 μM Erlo for the indicated time. After treatment, the cells were analysed using immunoblotting. (c) HT-29 cells were treated with Gefi at the indicated concentrations for 30 min. After treatment, the cells were analysed using immunoblotting. (d) HT-29 cells were treated with 20 μM Gefi for the indicated time. After treatment, the cells were analysed using immunoblotting. (e) HeLa cells were treated with EGF at the indicated concentrations for 15min. After treatment, the cells were analysed using immunoblotting. (f) HeLa cells were treated with 250ng/mL EGF for the indicated time. After treatment, the cells were analysed using immunoblotting.

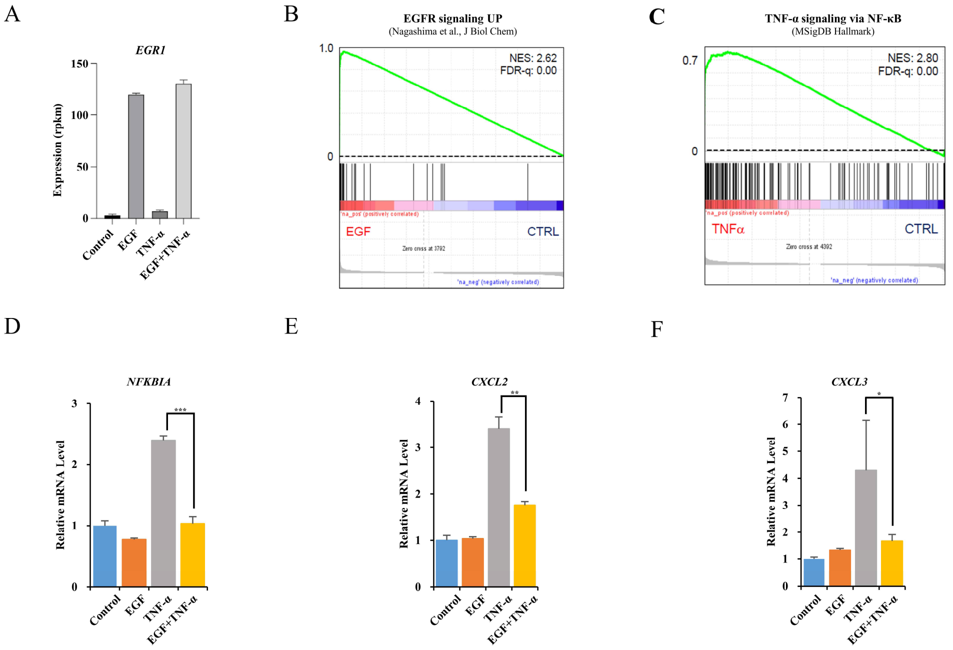

Supplementary Figure 8. EGF treatment suppresses transcription of *NFKBIA, CXCL2*, and *CXCL3*.

(a) mRNA level of EGR1. (b) GSEA of HeLa cells in the presence or absence of EGF. (c) GSEA of HeLa cells in the presence or absence of TNF-α. (d–f) Relative mRNA levels of NFKBIA, CXCL2, and CXCL3 in HeLa cells. Data are the mean ± standard deviation (S.D.), n = 3, with ns non-significance, *P < 0.05, **P < 0.01, and ***P < 0.001 at each point compared to the indicated graph with the two-sided Student's t test (d-f).

A

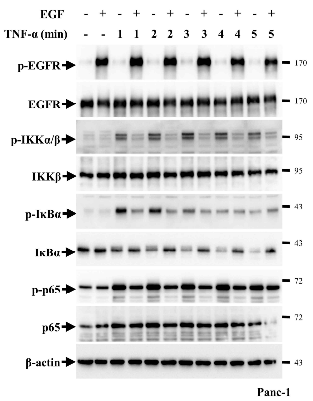

Supplementary Figure 9. EGF treatment suppresses TNF-α-mediated NF-κB signaling pathway in Panc-1. (a) Panc-1 cells were treated with EGF for 15 min and treated with TNF-α for the indicated time. After treatment, the cells were analysed using immunoblotting.

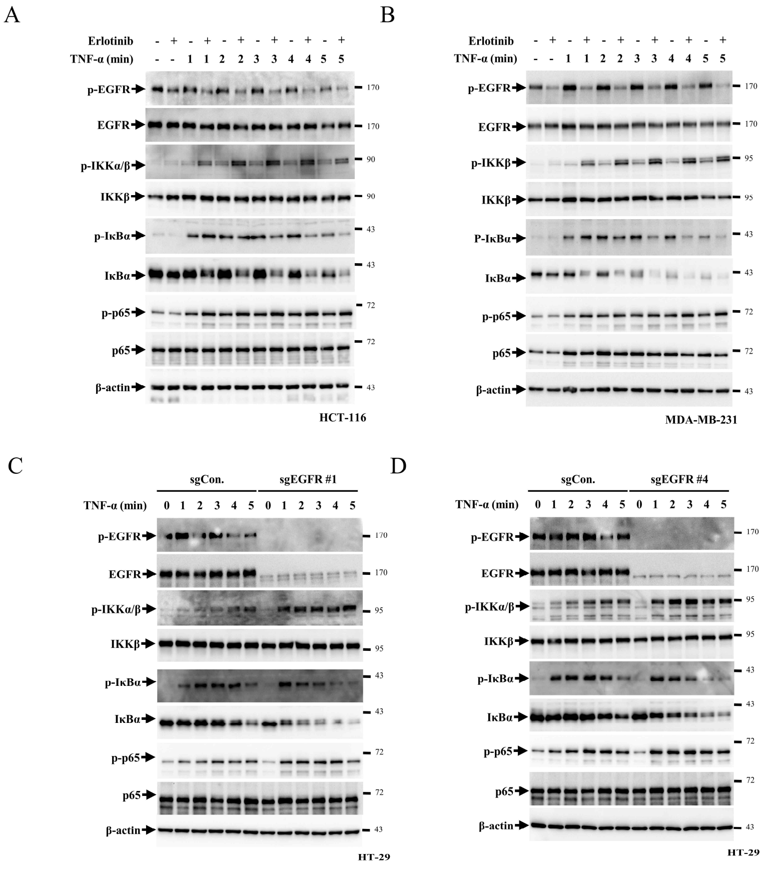

Supplementary Figure 10. EGFR inhibition or depletion promotes TNF-α-mediated NF-κB signaling pathway.

(a) HCT-116 cells were treated with 20 µM Erlo for 30 min and with TNF-α for the indicated time. After treatment, the cells were analysed by immunoblotting. (b) MDA-MB-231 cells were treated with 20 µM Erlo for 30 min, and with TNF-α for the indicated times. After treatment, the cells were analysed using immunoblotting. (c-d) EGFR WT and KO HT-29 cells were treated with TNF-α for the indicated time. After treatment, the cells were analysed using immunoblotting.

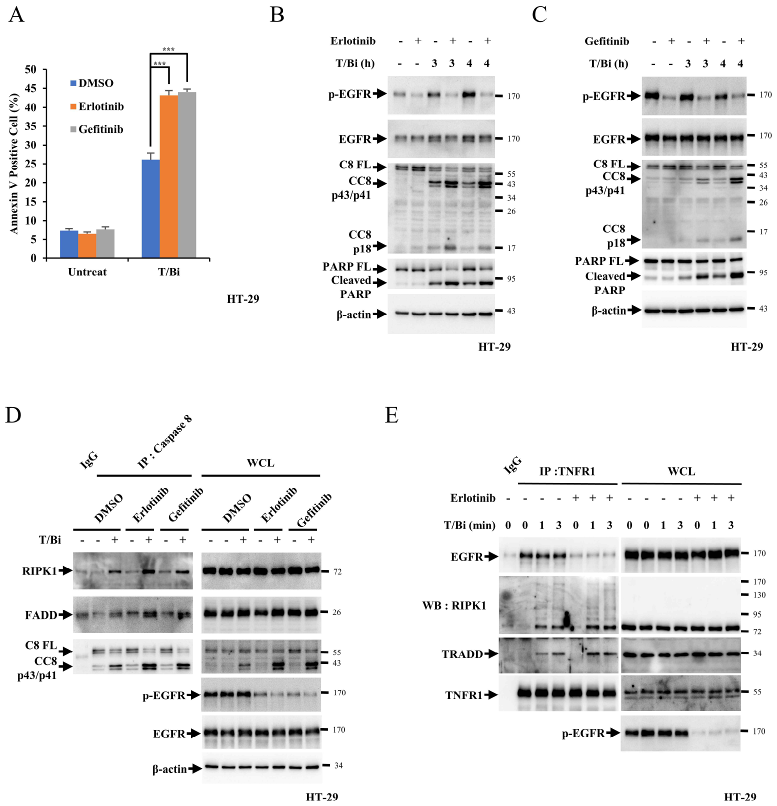

Supplementary Figure 11. EGFR inhibition increases TNF-α-induced apoptosis.

(a) HT-29 cells were treated with 30 ng/mL TNFα and 1 μM Birinapant (T/Bi) for 4 h in pretreatment or absence of 20 μM erlotinib or gefitinib for 30 min. After inducing apoptosis, cells were stained with annexin V-FITC and 7-AAD for 15min before analysing using flow cytometry. (b, c) Cleavage of caspase-8 and PARP were determined by western blotting. (d) HT-29 cells were treated with 20 μM Erlo or 20 μM Gefi for 30 min and then with T/Bi for 4 h. After treatment, the cells were lysed with lysis buffer and incubated with the anti-Caspase-8 antibodies. Samples were precipitated by incubating with protein G agarose, followed by immunoblotting analysis using the indicated antibodies. (e) HT-29 cells were treated with 20 μM Erlo for 30 min and with T/Bi for the indicated time. After treatment, the cells were lysed with lysis buffer and incubated with the anti-TNFR1 antibodies. Samples were precipitated by incubating with protein G agarose, followed by immunoblotting analysis using the indicated antibodies. Data are the mean ± standard deviation (S.D.), n = 3, with ns non-significance, $*P < 0.05$, $**P < 0.01$, and $***P < 0.001$ at each point compared to the indicated graph with the two-sided Student's t test (a).

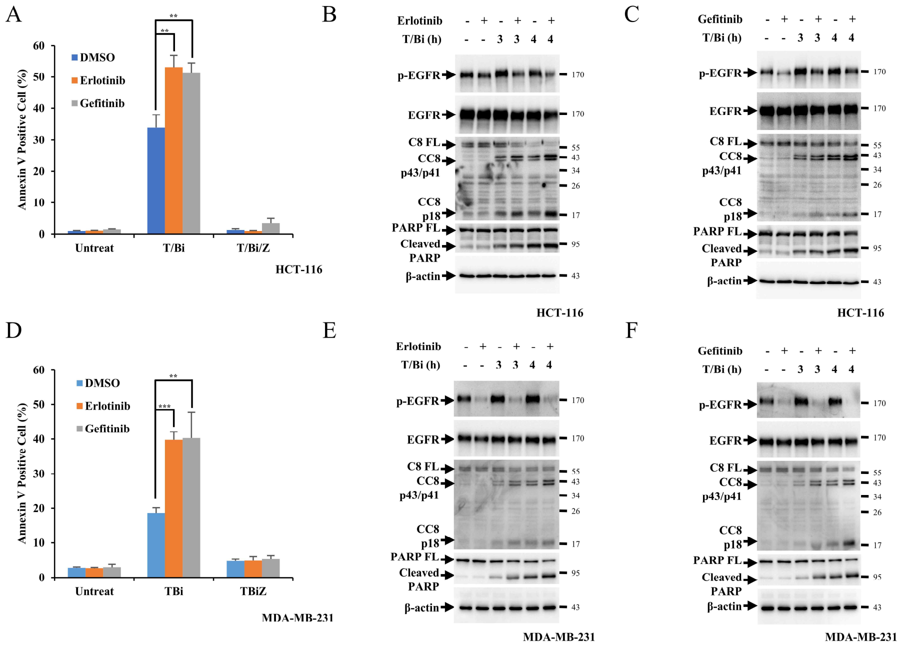

Supplementary Figure 12. EGFR inhibition increases TNF-α-induced apoptosis in HCT-116 and MDA-MB-231 cells.

(a) HCT-116 cells were treated with 20 ng/mL TNFα and 1 μM Birinapant (T/Bi) for 4 h in pretreatment or absence of 20 μM erlotinib or gefitinib for 30min. After inducing apoptosis, cells were stained with annexin V-FITC and 7-AAD for 15 min before analysing by flow cytometry. (b, c) Cleavage of caspase-8 and PARP were determined by western blotting. (d) MDA-MB-231 cells were treated with 20 ng/mL TNFα and 1 μM Birinapant (T/Bi) for 4 h in pretreatment or absence of 20 μM erlotinib or gefitinib for 30 min. After inducing apoptosis, cells were stained with annexin V-FITC and 7-AAD for 15min before analysis with flow cytometry. (e, f) Cleavage of caspase-8 and PARP were determined by western blotting. Data are the mean ± standard deviation (S.D.), n = 3, with ns non-significance. *$P < 0.05$, **$P < 0.01$, and ***$P < 0.001$ at each point compared to indicated graph with the two-sided Student's t test (a, d).

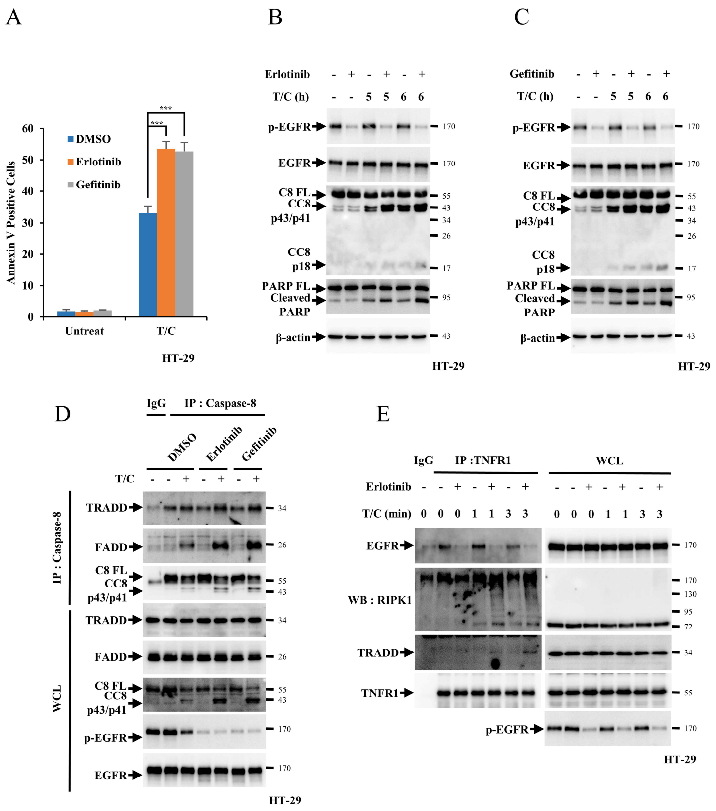

Supplementary Figure 13. EGFR inhibition increases complex IIa-dependent cell death.

(a) HT-29 cells were treated with 30 ng/mL TNFα and 5 μg/mL cycloheximide (T/C) for 5 h in pretreatment or absence of 20 μM erlotinib or gefitinib for 30 min. After inducing apoptosis, cells were stained with annexin V-FITC and 7-AAD for 15 min before analysing using flow cytometry. (b, c) Cleavage of caspase-8 and PARP were determined by western blotting. (d) HT-29 cells were treated with 20 μM Erlo or 20 μM Gefi for 30 min and then with T/C for the indicated times. After treatment, the cells were lysed with lysis buffer and incubated with the anti-Caspase-8 antibodies. Samples were precipitated by incubating with protein G agarose, followed by immunoblotting analysis using the indicated antibodies. (e) HT-29 cells were treated with 20 μM Erlo for 30 min and with T/C for the indicated time. After treatment, the cells were lysed with lysis buffer and incubated with the anti-TNFR1 antibodies. Samples were precipitated by incubating with protein G agarose, followed by immunoblotting analysis using the indicated antibodies. Data are the mean ± standard deviation (S.D.), n = 3, with ns non-significance, *P < 0.05, **P < 0.01, and ***P < 0.001 at each point compared to the indicated graph with the two-sided Student's t test (a).

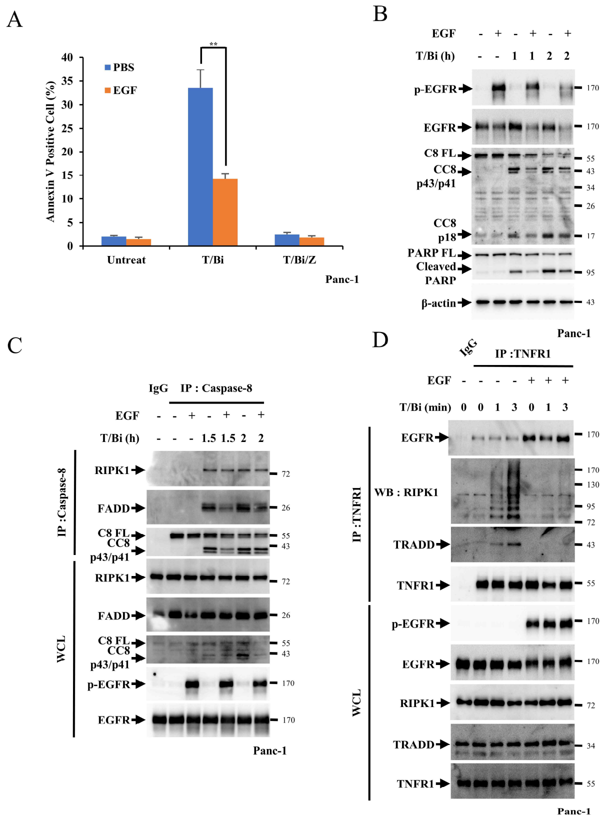

Supplementary Figure 14. EGFR activation decreases TNF-α-induced apoptosis in Panc-1 cells.

(a) Panc-1 cells were treated with 20 ng/mL TNFα and 1 μM Birinapant (T/Bi) for 2 h in the pretreatment or absence of 250 ng/mL EGF for 15 min. After inducing apoptosis, cells were stained with annexin V-FITC and 7-AAD for 15min before analysis using flow cytometry. (b) Cleavage of caspase-8 and PARP were determined by western blotting. (c) Panc-1 cells were treated with T/Bi for the indicated times in the pretreatment or absence of 250 ng/mL EGF for 15 min. After treatment, the cells were lysed with lysis buffer and incubated with the anti-caspase-8 antibodies. Samples were precipitated by incubating with protein G agarose, followed by immunoblotting analysis using the indicated antibodies. (d) Panc-1 cells were treated with T/Bi for the indicated time in the pretreatment or absence of 250 ng/mL EGF for 15 min. After treatment, the cells were lysed with lysis buffer and incubated with the anti-TNFR1 antibodies. Samples were precipitated by incubating with protein G agarose, followed by immunoblotting analysis using the indicated antibodies. Data are the mean ± standard deviation (S.D.), n = 3, with ns non-significance, $*P < 0.05$, $**P < 0.01$, and $***P < 0.001$ at each point compared to indicated graph with the two-sided Student's t test (a).

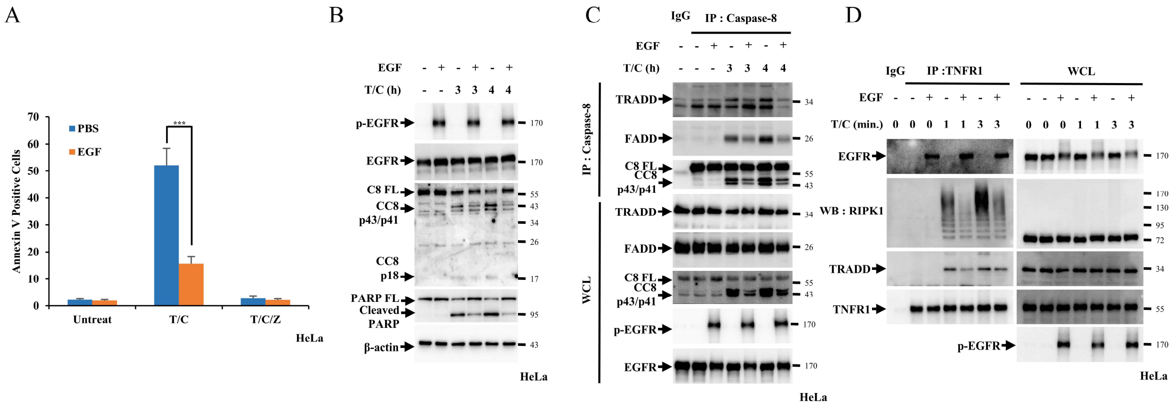

Supplementary Figure 15. EGFR activation inhibits complex IIa-dependent cell death.

(a) HeLa cells were treated with 20 ng/ mL TNFα and 5 µg/mL cycloheximide (T/C) for 4 h in the pretreatment or absence of 250 ng/mL EGF for 15 min. After apoptosis was induced, the cells were stained with Annexin V-FITC and 7-AAD for 15 min before analysis using flow cytometry. (b) Cleavage of caspase-8 and PARP was determined by western blotting. (c) HeLa cells were treated with T/C for the indicated times in the pretreatment or absence of 250 ng/mL EGF for 15 min. After treatment, cells were lysed with lysis buffer and incubated with an anti-caspase-8 antibodies. Samples were precipitated by incubation with protein G agarose, followed by immunoblotting using the indicated antibodies. (d) HeLa cells were treated with T/C for the indicated times in the pretreatment or absence of 250 ng/mL EGF for 15 min. After treatment, the cells were lysed with lysis buffer and incubated with an anti-TNFR1 antibodies. Samples were precipitated by incubation with protein G agarose, followed by immunoblotting using the indicated antibodies. Data are the mean ± standard deviation (S.D.), n = 3, with ns non-significance, *P < 0.05, **P < 0.01, and ***P < 0.001 at each point compared to the indicated graph with the two-sided Student's t test (a).

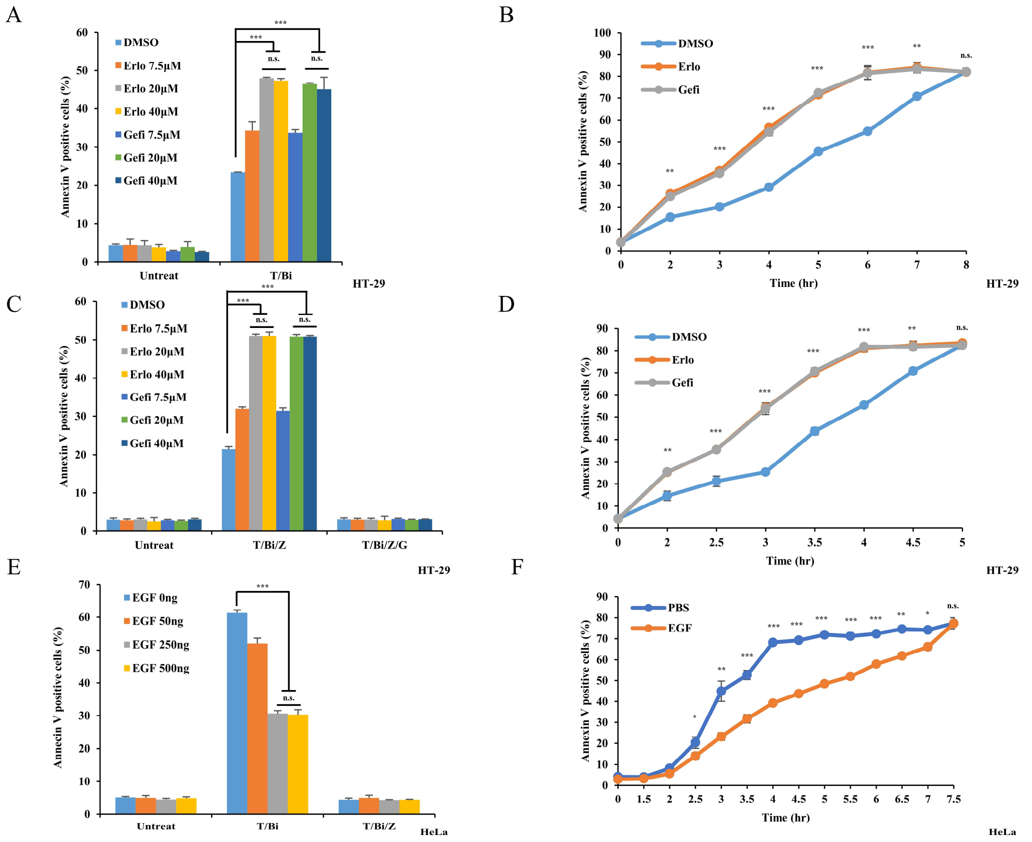

Supplementary Figure 16. EGFR activation delays TNF-α-induced cell death.

(a) HT-29 cells were treated with 30 ng/mL TNFα and 1 μM Birinapant (T/Bi) for 4 h in pretreatment or absence of erlotinib or gefitinib at the indicated concentrations for 30 min. After inducing apoptosis, cells were stained with annexin V-FITC and 7-AAD for 15 min before analysing using flow cytometry. (b) HT-29 cells were treated with T/Bi for the indicated time in pretreatment or absence of 20 μM erlotinib or gefitinib for 30 min. After inducing apoptosis, cells were stained with annexin V-FITC and 7-AAD for 15 min before analysing using flow cytometry. (c) HT-29 cells were treated with 30 ng/mL TNFα, 1 μM Birinapant, and 30 μM z-VAD-fmk (T/Bi/Z) for 3 h in pretreatment or absence of erlotinib or gefitinib at the indicated concentrations for 30 min. After inducing necroptosis, cells were stained with annexin V-FITC and 7-AAD for 15 min before analysing using flow cytometry. (d) HT-29 cells were treated with T/Bi/Z for the indicated time in pretreatment or absence of 20 μM erlotinib or gefitinib for 30 min. After inducing necroptosis, cells were stained with annexin V-FITC and 7-AAD for 15 min before analysing using flow cytometry. (e) HeLa cells were treated with 20 ng/mL TNFα and 1 μM Birinapant (T/Bi) for 4 h in the pretreatment of absence of EGF at the indicated concentrations for 15 min. After inducing apoptosis, cells were stained with annexin V-FITC and 7-AAD for 15 min before analysis using flow cytometry.

(f) HeLa cells were treated with T/Bi for the indicated time in the pretreatment or absence of 250 ng/mL EGF for 15 min. After inducing apoptosis, cells were stained with annexin V-FITC and 7-AAD for 15 min before analysis using flow cytometry. Data are the mean ± standard deviation (S.D.), n = 3, with ns non-significance, *P < 0.05, **P < 0.01, and ***P < 0.001 at each point compared to indicated graph with the two-sided Student's t test (a-f).

A

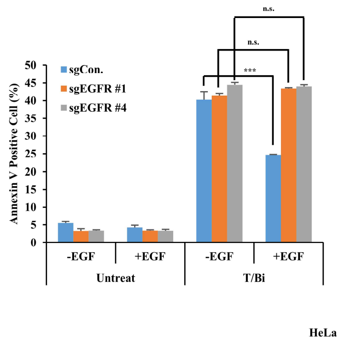

B

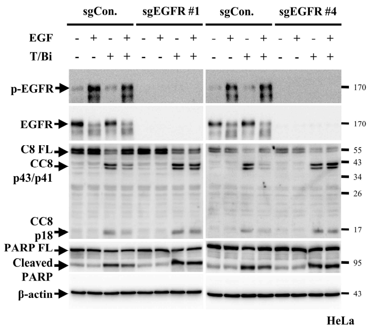

Supplementary Figure 17. EGFR depletion invalidates EGF-dependent cell death suppression.

(a) EGFR WT or KO HeLa cells were treated with 20 ng/mL TNF-α and 1 µM Birinapant (T/Bi) for 3 h in the pretreatment or absence of 250 ng/mL EGF for 15 min. After inducing apoptosis, cells were stained with annexin V-FITC and 7-AAD for 15 min before analysing by flow cytometry. (b) Cleavage of caspase-8 and PARP were determined by western blotting. Data are the mean ± standard deviation (S.D.), n = 3, with ns non-significance, *P < 0.05, **P < 0.01, and ***P < 0.001 at each point compared to indicated graph with the two-sided Student's t test (a).

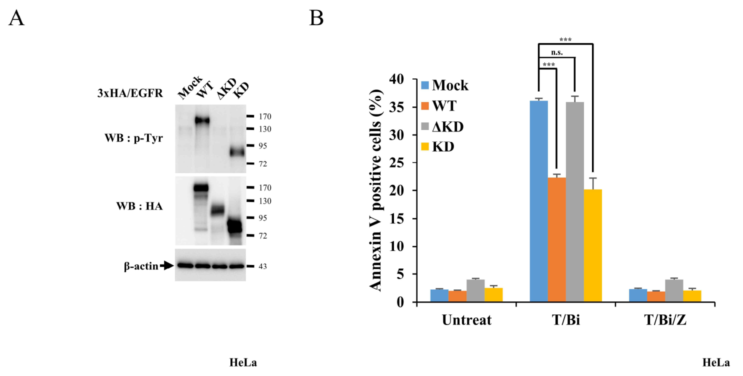

Supplementary Figure 18. EGFR kinase domain suppresses TNF-α-induced cell death.

(a) HeLa cell were transfected using the indicated plasmids, followed by immunoblotting analysis using the indicated antibodies. (b) 3xHA/EGFR WT, ΔKD, and KD expressed HeLa cells were treated with 20 ng/mL TNF-α and 1 μM Birinapant (T/Bi) for 3 h in the pretreatment or absence of 250 ng/mL EGF for 15 min. After inducing apoptosis, cells were stained with annexin V-FITC and 7-AAD for 15 min before analysing by flow cytometry. Data are the mean ± standard deviation (S.D.), n = 3, with ns non-significance, *P < 0.05, **P < 0.01, and ***P < 0.001 at each point compared to indicated graph with the two-sided Student's t test (b).

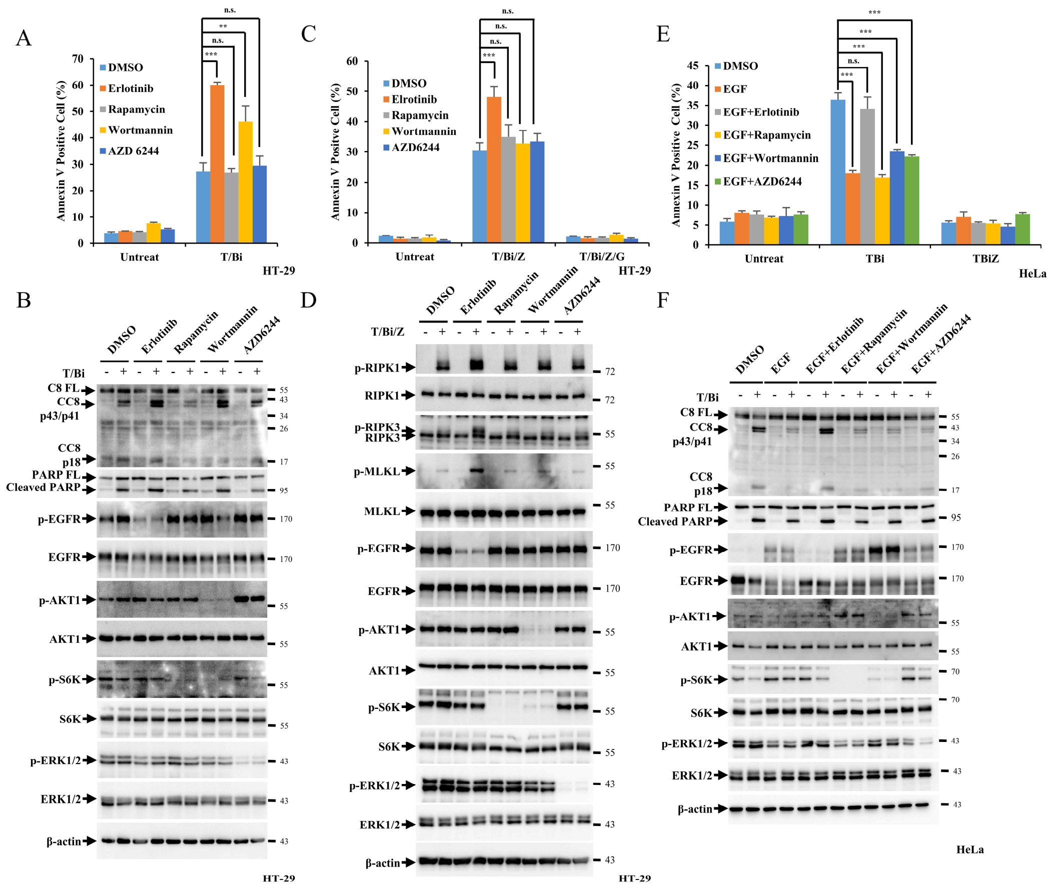

Supplementary Figure 19. EGFR directly suppresses TNF-α-induced cell death.

(a) HT-29 cells were treated with 30 ng/mL TNFα, and 1 μM Birinapant (T/Bi) for 4 h in pretreatment or absence of 20 μM erlotinib, 200 nM rapamycin, 100 nM wortmannin or 10μM AZD6244 for 30 min. After inducing apoptosis, cells were stained with annexin V-FITC and 7-AAD for 15 min before analysis using flow cytometry. (b) Cleavage of caspase-8 and PARP was determined using western blotting. (c) HT-29 cells were treated with 30 ng/mL TNFα, 1 μM Birinapant, 30 μM z-VAD-fmk (T/Bi/Z) for 3 h in the pretreatment or absence of 20 μM erlotinib, 200 nM rapamycin, 100 nM wortmannin or 10 μM AZD6244 for 30 min. After inducing necroptosis, cells were stained with annexin V-FITC and 7-AAD for 15 min before analysis using flow cytometry. (d) p-RIPK1, p-RIPK3 and p-MLKL were determined by western blotting. (e) HeLa cells were treated with 20 μM erlotinib, 200 nM rapamycin, 100 nM wortmannin, or 10μM AZD6244 for 30 min with pretreatment of 250 ng/mL EGF for 15 min. After that, HeLa cells were treated with T/Bi for 4 h. After inducing apoptosis, cells were stained with annexin V-FITC and 7-AAD for 15 min before analysis using flow cytometry. (f) Cleavage of caspase-8 and PARP were determined by western blotting. Data are the mean ± standard deviation (S.D.), n = 3, with ns non-significance, *P < 0.05, **P < 0.01, and ***P < 0.001 at each point compared to indicated graph with the two-sided

Student’s t test (a, c, and e).

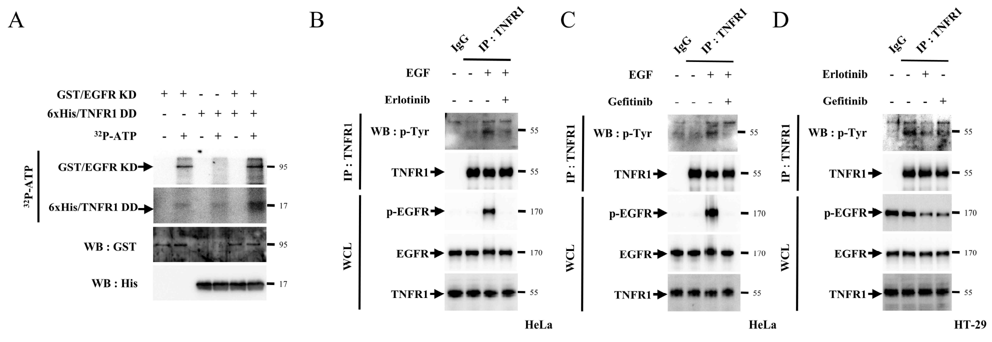

Supplementary Figure 20. EGFR kinase domain directly phosphorylates TNFR1 death domain.

(a) *In vitro* kinase assay and autoradiography were performed by incubating with recombinant proteins, GST/EGFR KD, 6xHis/TNFR1 DD, and [γ32P]-ATP. The mixtures were determined by western blotting. (b-c) HeLa cells were treated with 250 ng/mL EGF for 15 min in the pretreatment or absence of 20 μM erlotinib (Erlo) or 20 μM gefitinib (Gefi) for 30 min. After treatment, the HeLa cells were denatured using SDS, and lysed with lysis buffer and incubated with an anti-TNFR1 antibodies. Samples were precipitated by incubating with protein G agarose, followed by immunoblotting using the indicated antibodies. (d) HT-29 cells were treated with 20 μM Erlo or 20 μM Gefi for 30 min. After treatment, the HT-29 cells were denatured using SDS, and lysed with lysis buffer and incubated with an anti-TNFR1 antibodies. Samples were precipitated by incubating with protein G agarose, followed by immunoblotting using the indicated antibodies.

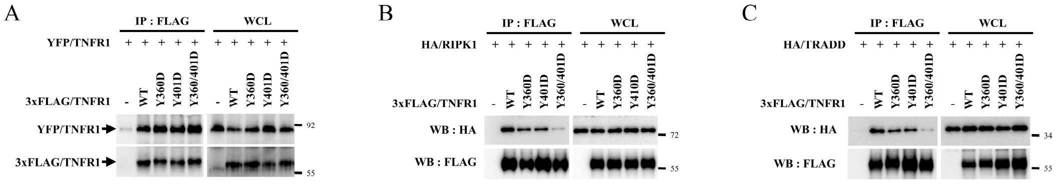

Supplementary Figure 21. TNFR1 Y360/401D mutation decreases interaction between TNFR1, RIPK1 and TRADD, not TNFR1-TNFR1 interaction.

(a–c) 293T cells were transfected with the indicated plasmid. After transfection, cells were lysed with lysis buffer and incubated with an anti-FLAG antibodies. Samples were precipitated by incubating with protein G agarose, followed by immunoblotting using the indicated antibodies.

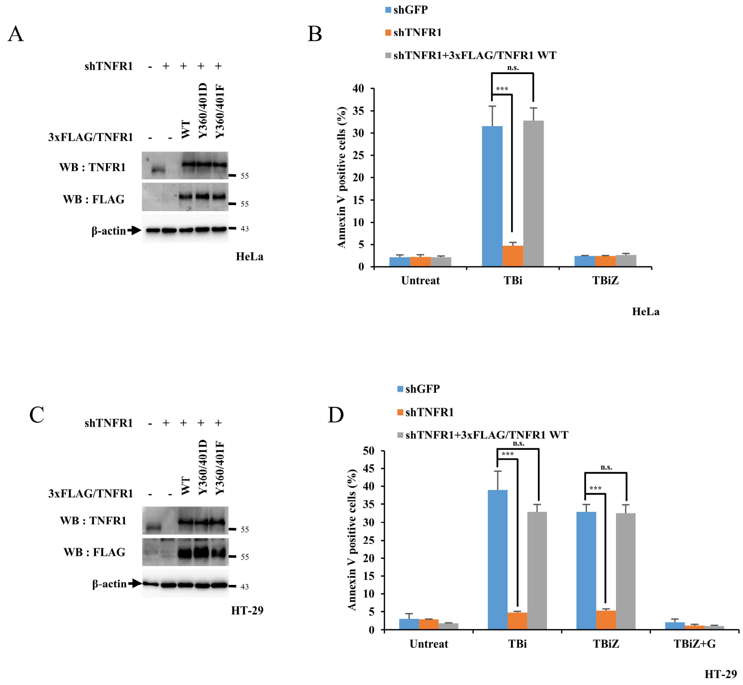

Supplementary Figure 22. TNFR1 deficiency inhibits TNF-α-induced cell death.

(a) shGFP, shTNFR1, shTNFR1+3xFLAG/TNFR1 expressed WT HeLa cells was determined by western blotting. (b) shGFP, shTNFR1, shTNFR1+3xFLAG/TNFR1 WT expressed HeLa cells were treated with 20 ng/mL TNF-α and 1 μM Birinapant (T/Bi) for 3 h. After inducing apoptosis, cells were stained with annexin V-FITC and 7-AAD for 15 min before analysis using flow cytometry. (c) shGFP, shTNFR1, shTNFR1+3xFLAG/TNFR1 WT expressed HT-29 cells was determined by western blotting. (d) shGFP, shTNFR1, shTNFR1+3xFLAG/TNFR1 WT expressed HT-29 cells were treated with 30 ng/mL TNF-α and 1 μM Birinapant (T/Bi) for 4 h or 30 ng/mL TNF-α, 1 μM Birinapant and 30 μM z-VAD-fmk (T/Bi/Z) for 3 h. After inducing apoptosis or necroptosis, cells were stained with annexin V-FITC and 7-AAD for 15 min before analysis using flow cytometry. Data are the mean ± standard deviation (S.D.), n = 3, with ns non-significance, $*P < 0.05$, $**P < 0.01$, and $***P < 0.001$ at each point compared to indicated graph with the two-sided Student's t test (b and d).

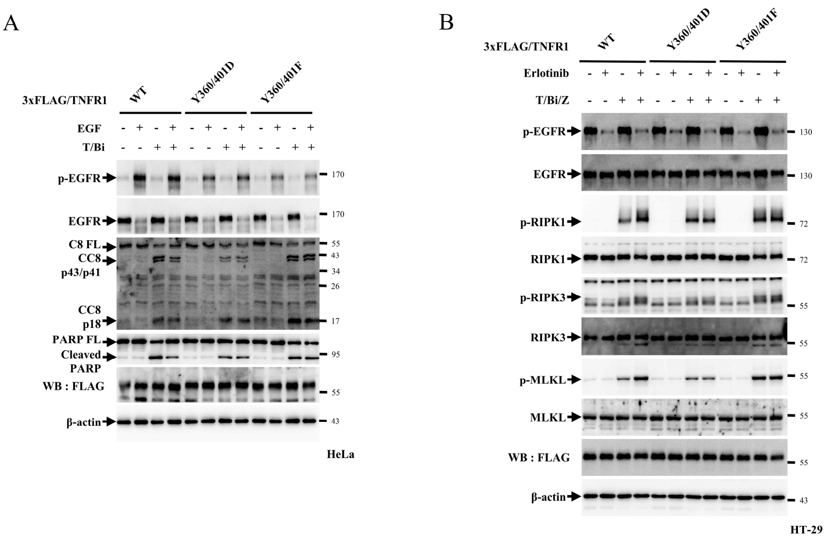

Supplementary Figure 23. Cell death markers of TNFR1 reconstituted cell lines.

(a) 3xFLAG/TNFR1 WT, Y360/401D, and Y360/401F reconstituted HeLa cells were treated with 20 ng/mL TNF-α and 1 μM Birinapant (T/Bi) for 3 h in the pretreatment or absence of 250 ng/mL EGF for 15 min. After inducing apoptosis, cleavage of caspase-8 and PARP were determined by western blotting. (b) 3xFLAG/TNFR1 WT, Y360/401D, and Y360/401F reconstituted HT-29 cells were treated with 30 ng/mL TNFα, 1 μM Birinapant, 30 μM z-VAD-fmk (T/Bi/Z) for 3 h. After treatment, p-RIPK1, p-RIPK3 and p-MLKL were determined by western blotting.

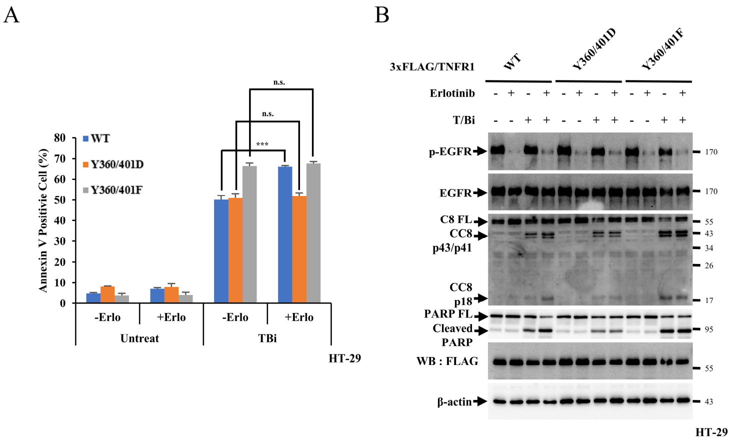

Supplementary Figure 24. Tyr 360 and 401 in TNFR1 is important for EGFR inhibition-mediated apoptosis increase.

(a) 3xFLAG/TNFR1 WT, Y360/401D, and Y360/401F reconstituted HT-29 cells were treated with 20 ng/mL TNFα and 1 μM Birinapant (T/Bi) for 4 h. After treatment, the cells were stained with annexin V-FITC and 7-AAD for 15 min, and then analysis via flow cytometry. (b) 3xFLAG/TNFR1 WT, Y360/401D, and Y360/401F reconstituted HT-29 cells were treated with T/Bi for 4 h. After treatment, cleavage of caspase-8 and PARP were determined by western blotting. Data are the mean ± standard deviation (S.D.), n = 3, with ns non-significance, $^{*}P < 0.05$, $^{**}P < 0.01$, and $^{***}P < 0.001$ at each point compared to indicated graph with the two-sided Student's t test (a).

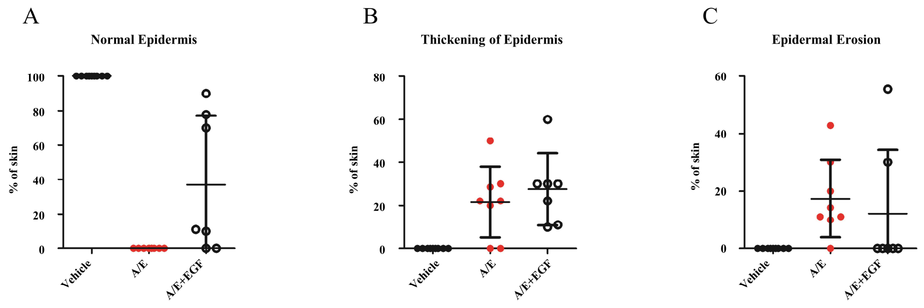

Supplementary Figure 25. The skin scores for each treatment.

(a) Percentage contribution of normal epidermis in each mouse. (b) Percentage contribution of thickening of epidermis in each mouse. (c) Percentage contribution of epidermis erosion in each mouse.

Supplementary Table Legends

Table S1. Raw data of HeLa cell RNA sequencing.

Raw data of Fig. 2A-2C and Fig. S8A-S8C

Table S2. Skin Scores of the mouse model.

Skin scores of Fig. 7D-7F and Fig. S25
