# Supplementary material for: EGFR inhibits TNF-α-mediated pathway by phosphorylating TNFR1 at tyrosine 360 and 401
Source: Cell Death Differ. 2024 May 24;31(10):1318–32. doi: 10.1038/s41418-024-01316-3 (PMC11445491; doi:10.1038/s41418-024-01316-3)

Figure 1

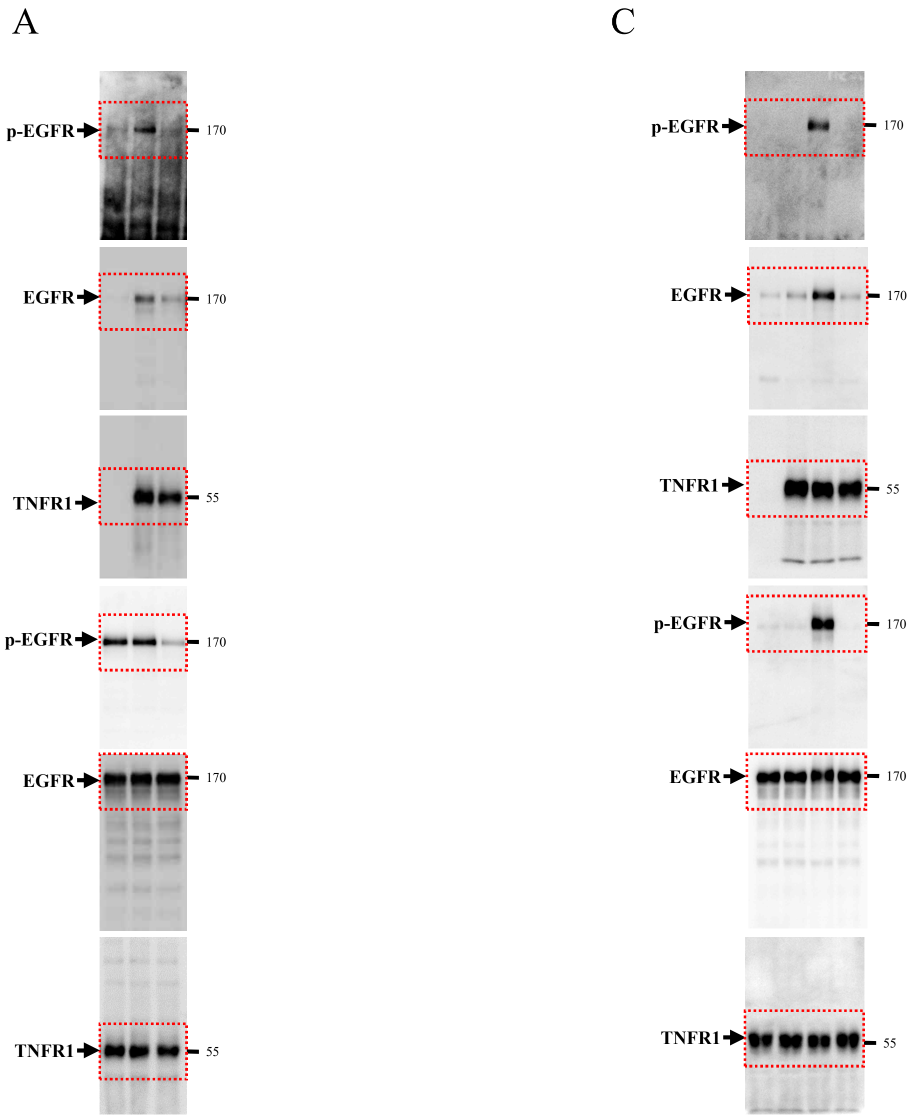
A
p-EGFR
170
EGFR
170
TNFR1
55
p-EGFR
170
EGFR
170
TNFR1
55
C
p-EGFR
170
EGFR
170
TNFR1
55
p-EGFR
170
EGFR
170
TNFR1
55

Figure 2

D

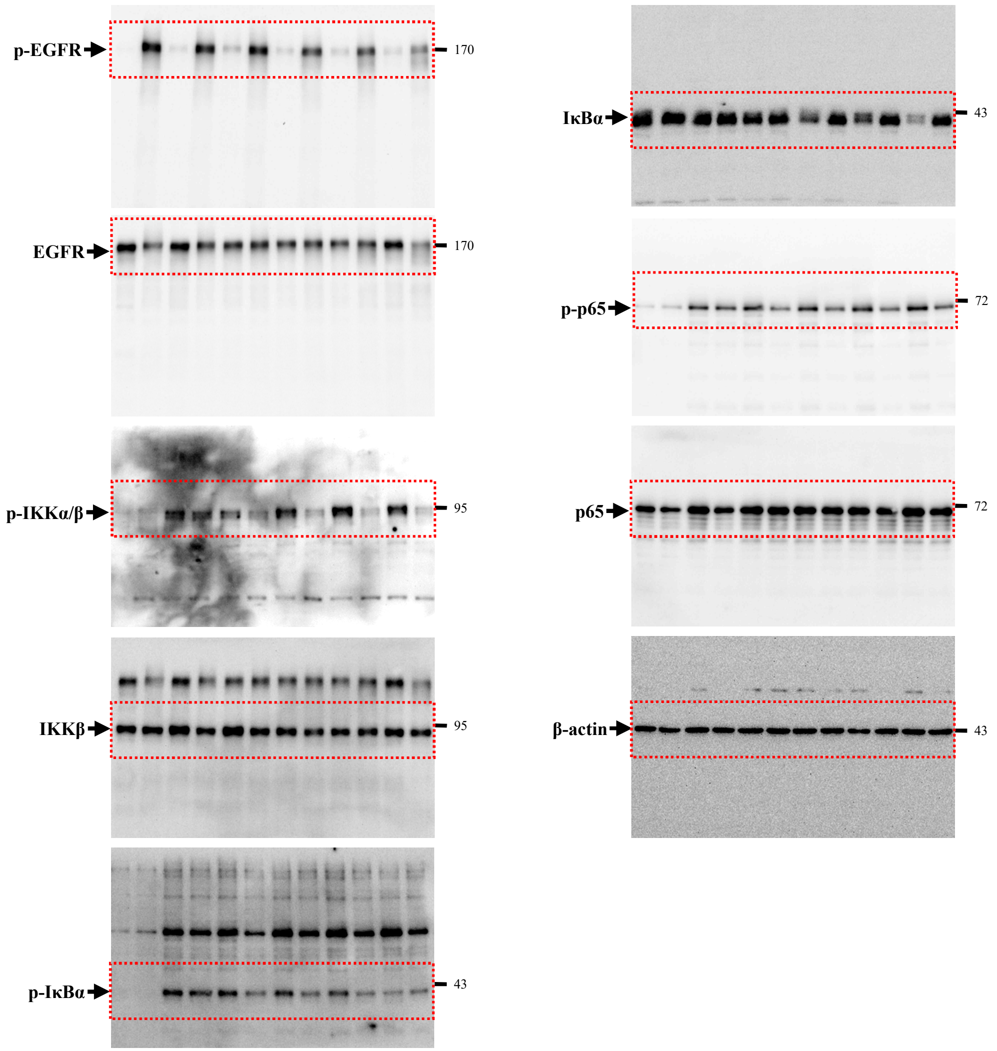

p-EGFR
170
EGFR
170
p-IKKα/β
95
IKKβ
95
p-IκBα
43
IκBα
43
p-p65
72
p65
72
β-actin
43

Figure 2

E

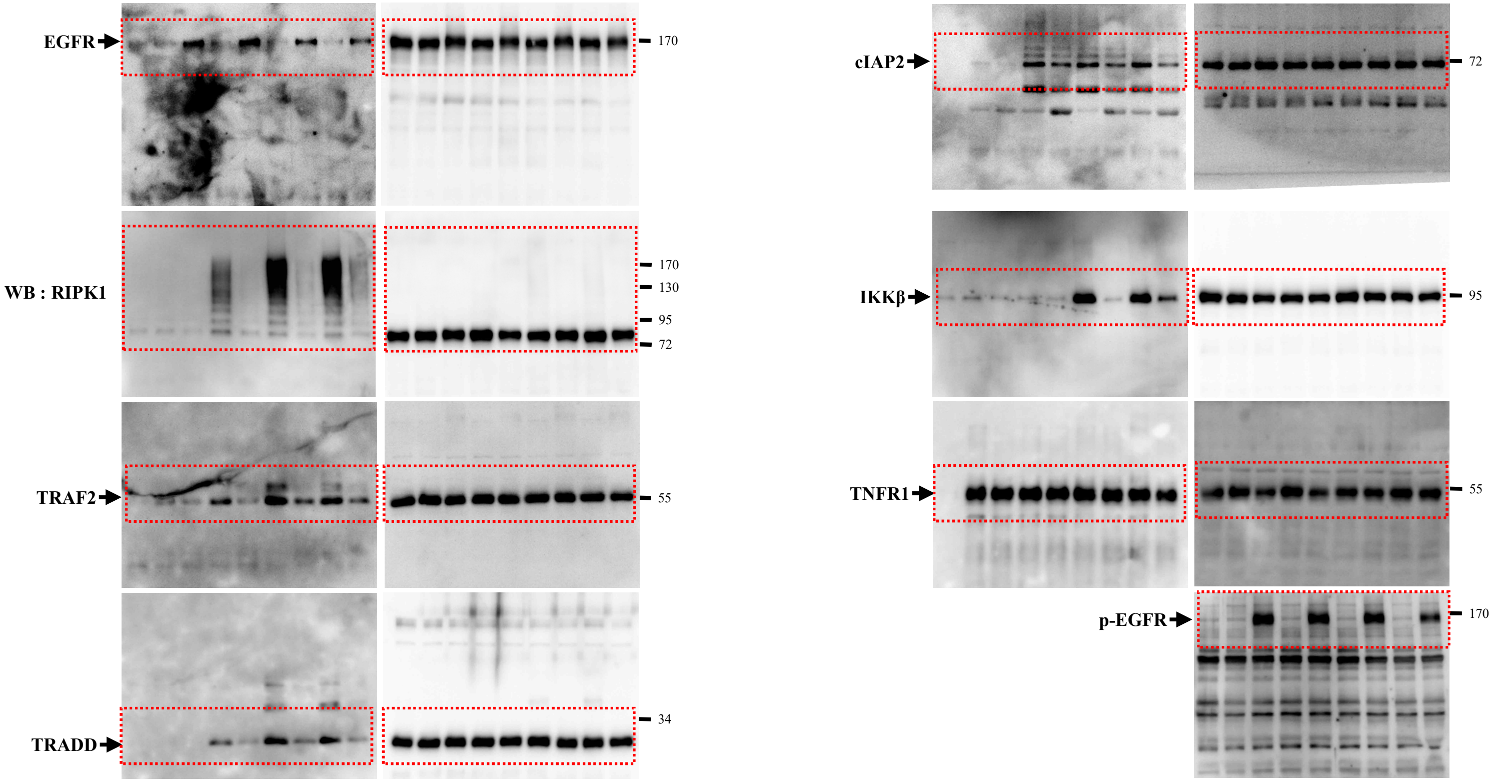

EGFR
170
WB : RIPK1
170
130
95
72
TRAF2
55
TRADD
34
cIAP2
72
IKKβ
95
TNFR1
55
p-EGFR
170

Figure 2

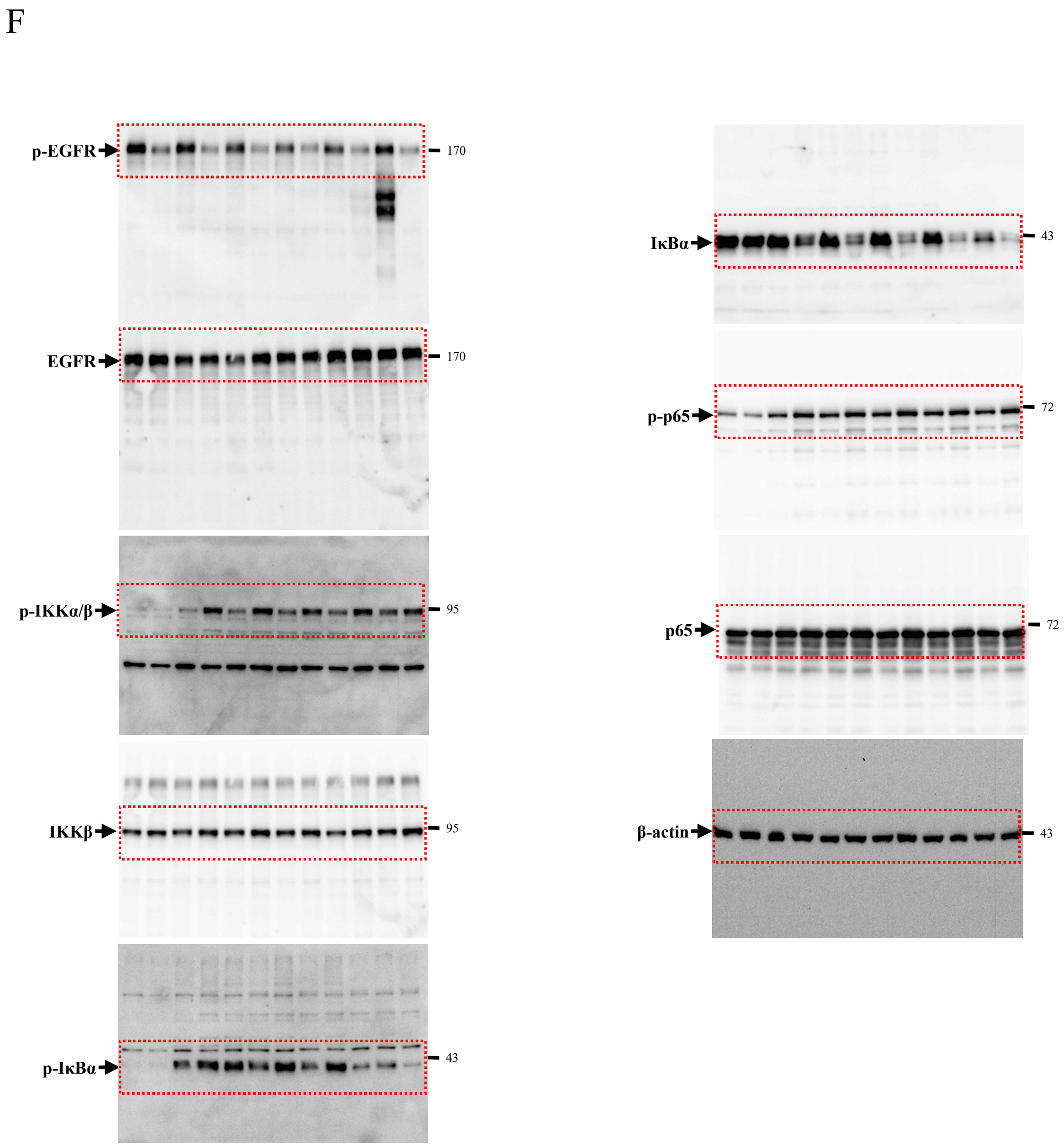
F
p-EGFR
170
EGFR
170
p-IKKα/β
95
IKKβ
95
p-IκBα
43
IκBα
43
p-p65
72
p65
72
β-actin
43

Figure 2

G

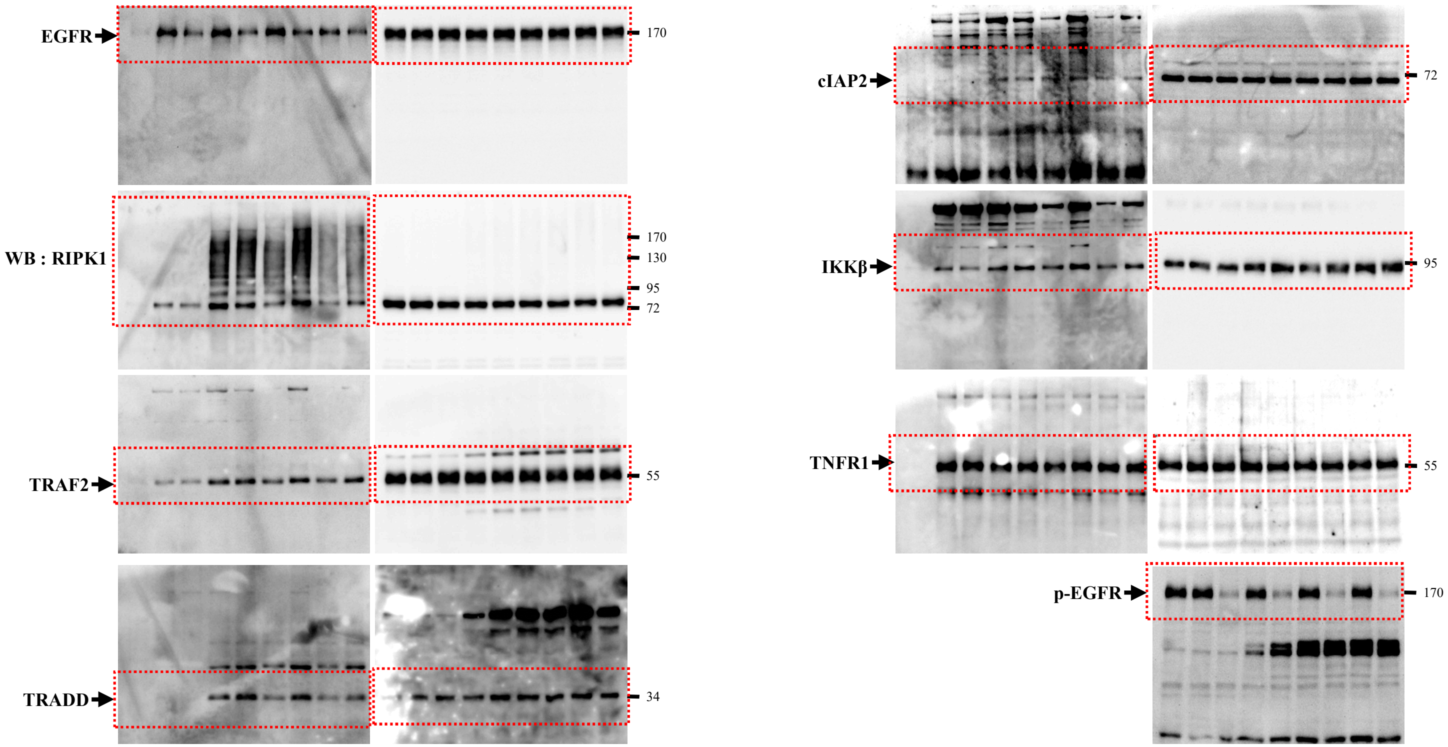
EGFR
170
WB : RIPK1
170
130
95
72
TRAF2
55
TRADD
34
cIAP2
72
IKKβ
95
TNFR1
55
p-EGFR
170

Figure 3

B

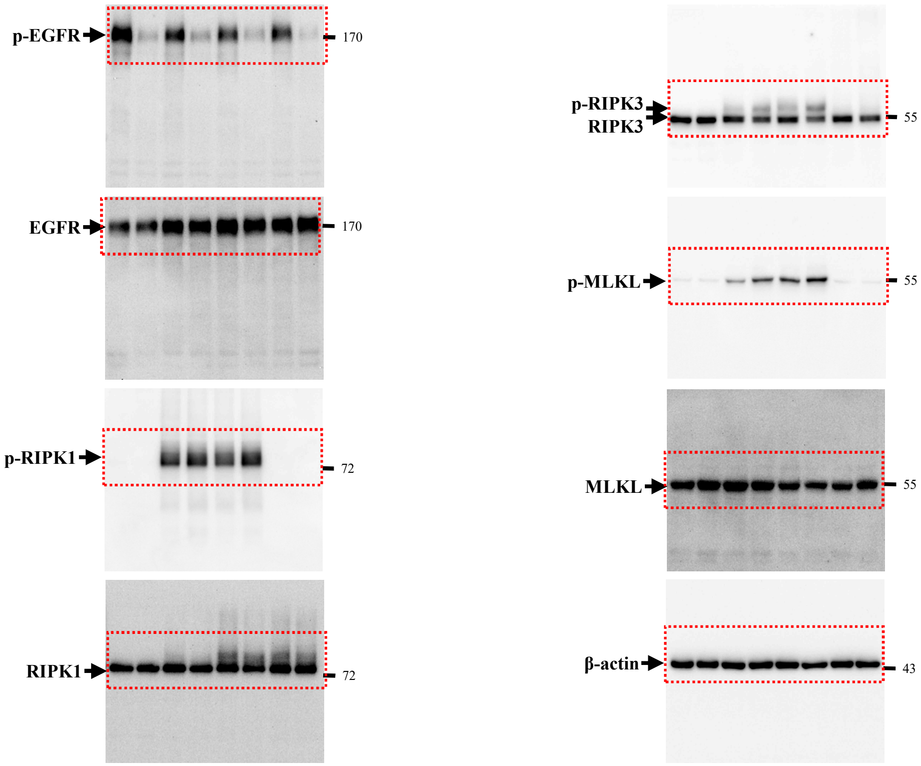

p-EGFR
170
EGFR
170
p-RIPK1
72
RIPK1
72
p-RIPK3
RIPK3
55
p-MLKL
55
MLKL
55
β-actin
43

Figure 3

C

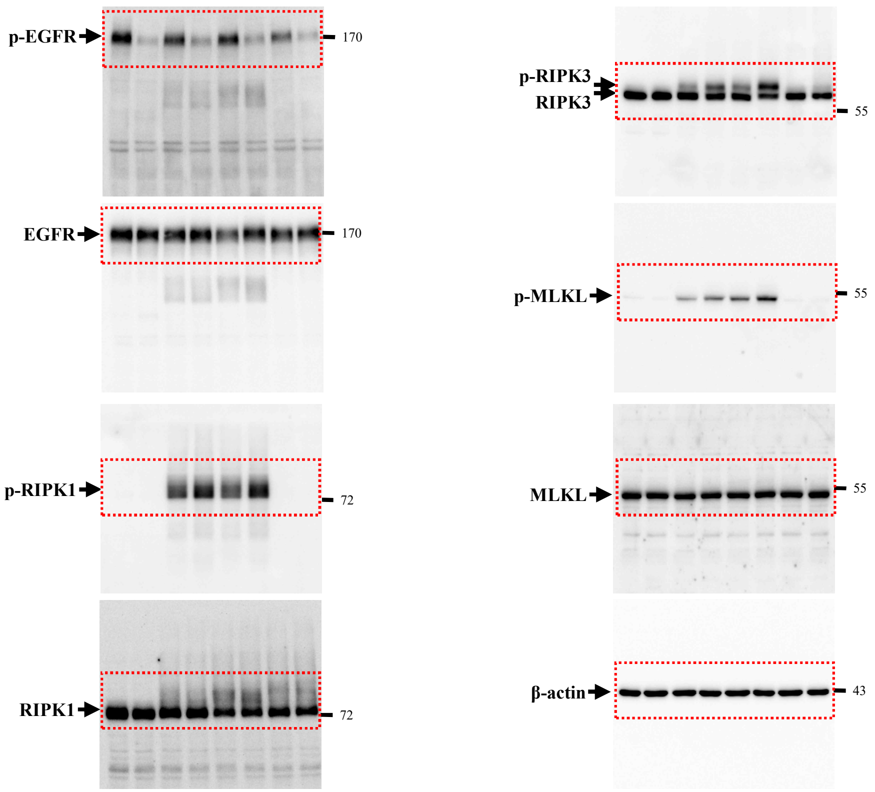

p-EGFR
170
EGFR
170
p-RIPK1
72
RIPK1
72
p-RIPK3
RIPK3
55
p-MLKL
55
MLKL
55
β-actin
43

Figure 3

D

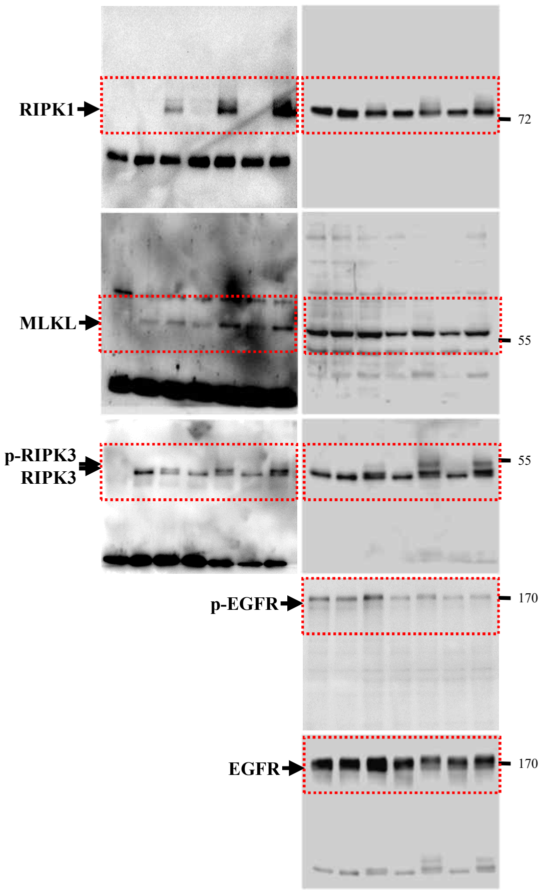

RIPK1
72
MLKL
55
p-RIPK3
RIPK3
55
p-EGFR
170
EGFR
170

E

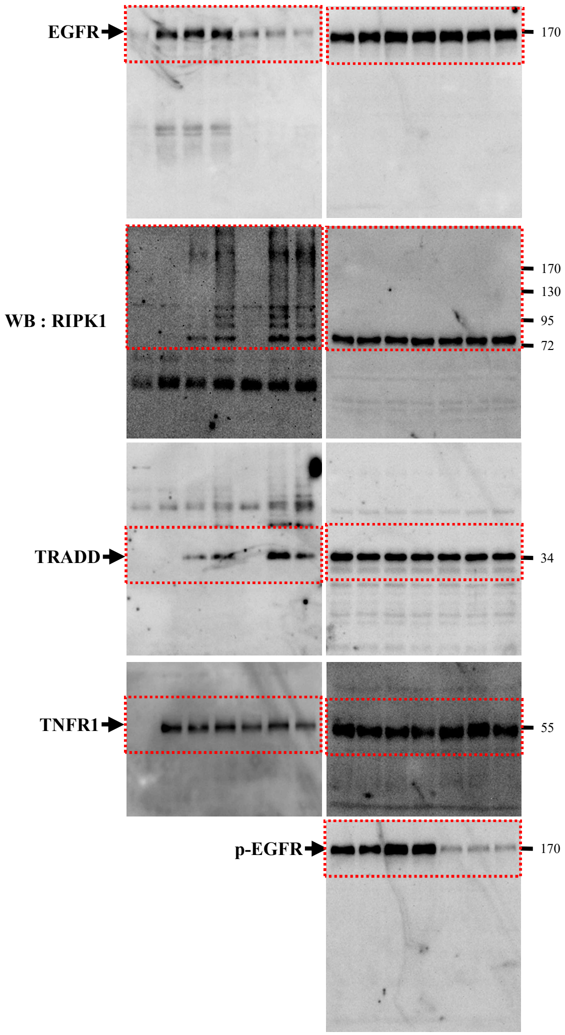

EGFR
170
WB : RIPK1
170
130
95
72
TRADD
34
TNFR1
55
p-EGFR
170

Figure 4

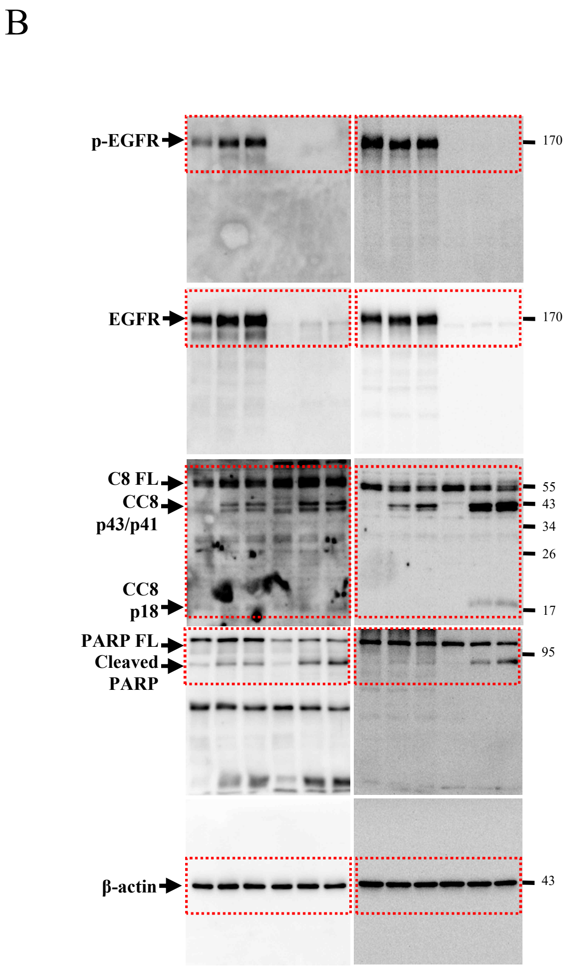
B
p-EGFR
170
EGFR
170
C8 FL
CC8
p43/p41
CC8
p18
55
43
34
26
17
PARP FL
Cleaved
PARP
95
β-actin
43

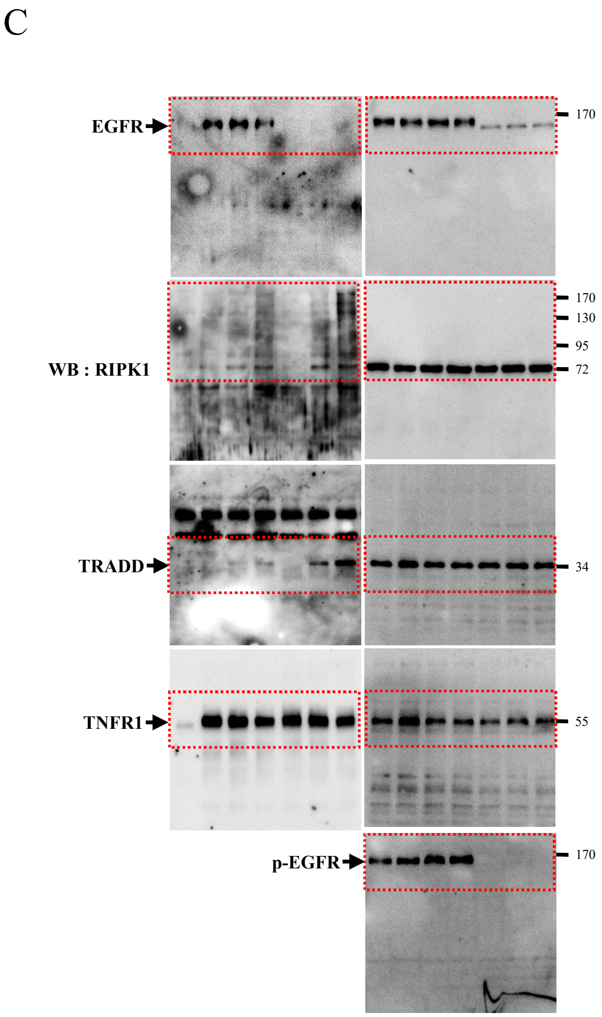
C
EGFR
170
WB : RIPK1
170
130
95
72
TRADD
34
TNFR1
55
p-EGFR
170

Figure 4

E

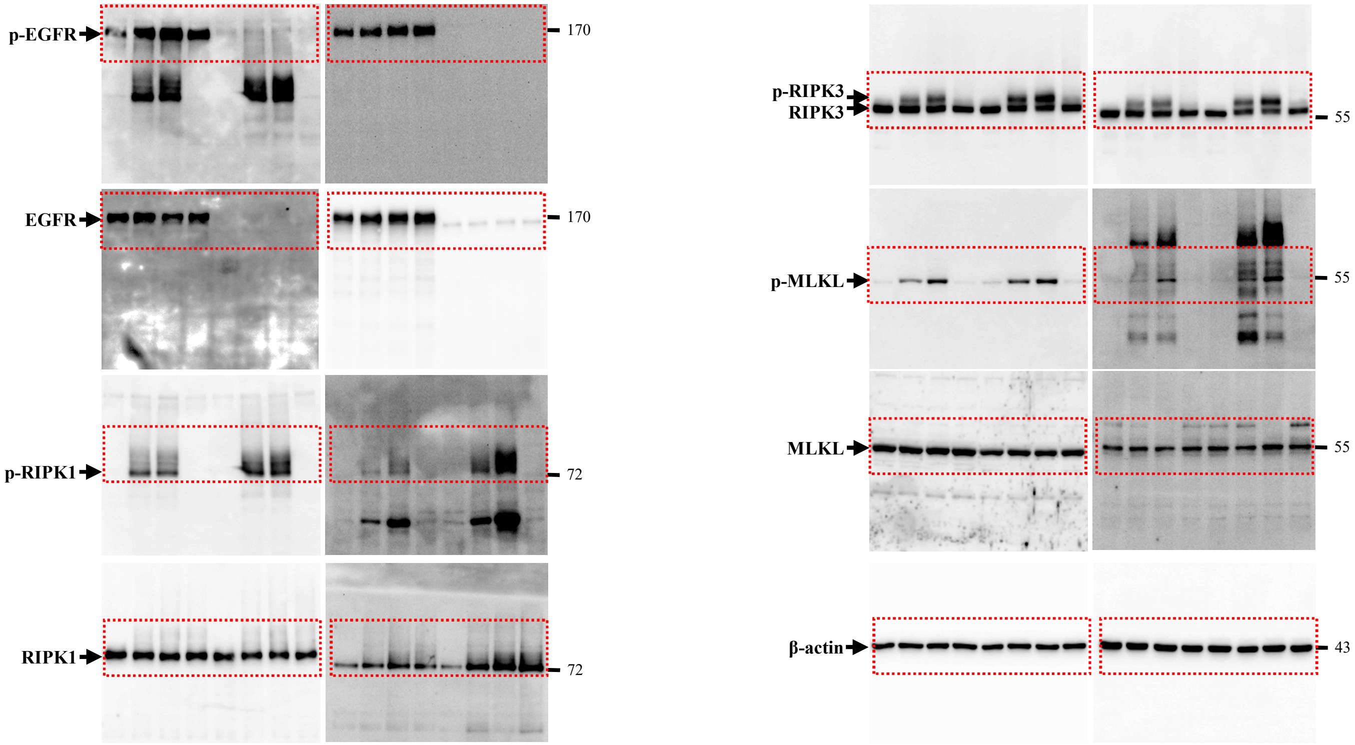

p-EGFR
170
EGFR
170
p-RIPK1
72
RIPK1
72
p-RIPK3
RIPK3
55
p-MLKL
55
MLKL
55
β-actin
43

Figure 4

F

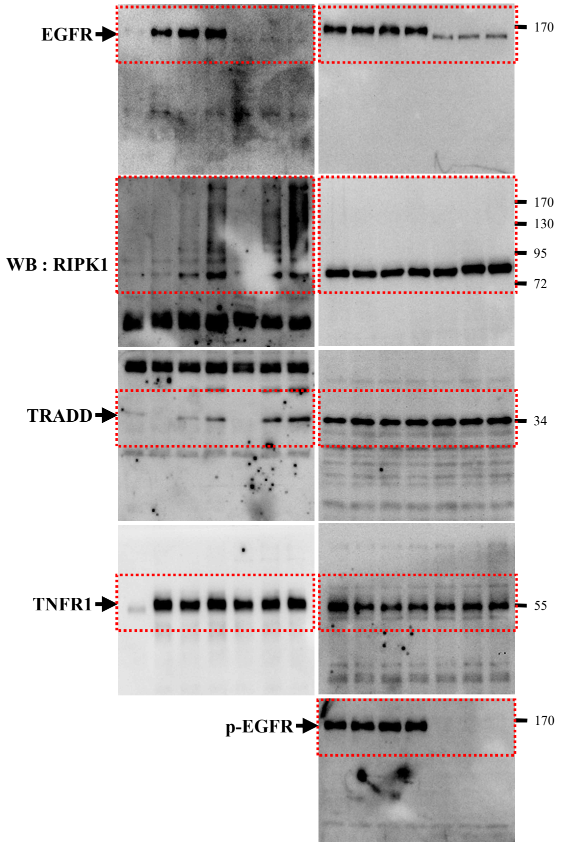

Figure 5

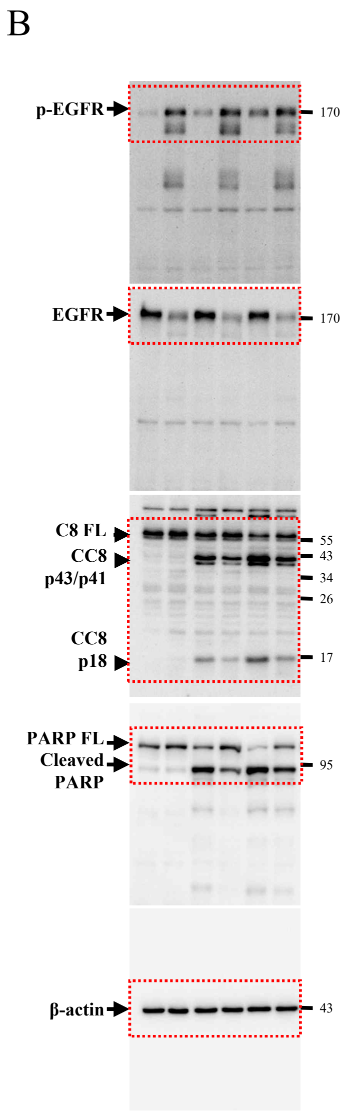

B
p-EGFR
170
EGFR
170
C8 FL
CC8
p43/p41
55
43
34
26
CC8
p18
17
PARP FL
Cleaved
PARP
95
β-actin
43

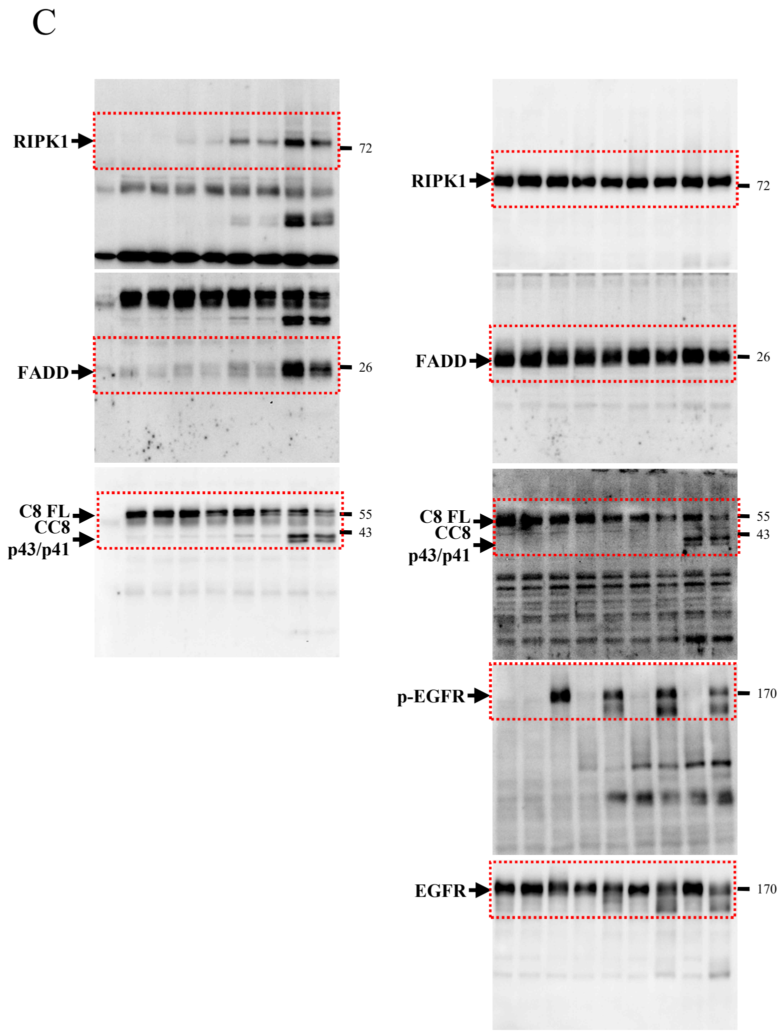

C
RIPK1
72
FADD
26
C8 FL
CC8
p43/p41
55
43
RIPK1
72
FADD
26
C8 FL
CC8
p43/p41
55
43
p-EGFR
170
EGFR
170

Figure 5

D

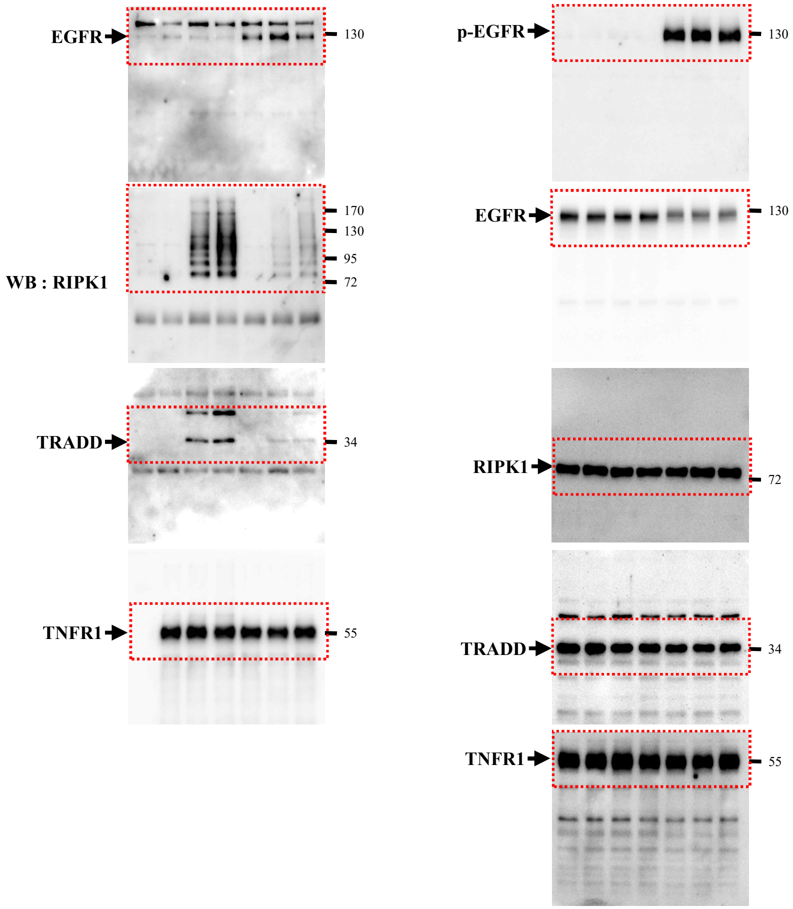
EGFR
130
WB : RIPK1
170
130
95
72
TRADD
34
TNFR1
55
p-EGFR
130
EGFR
130
RIPK1
72
TRADD
34
TNFR1
55

Figure 6

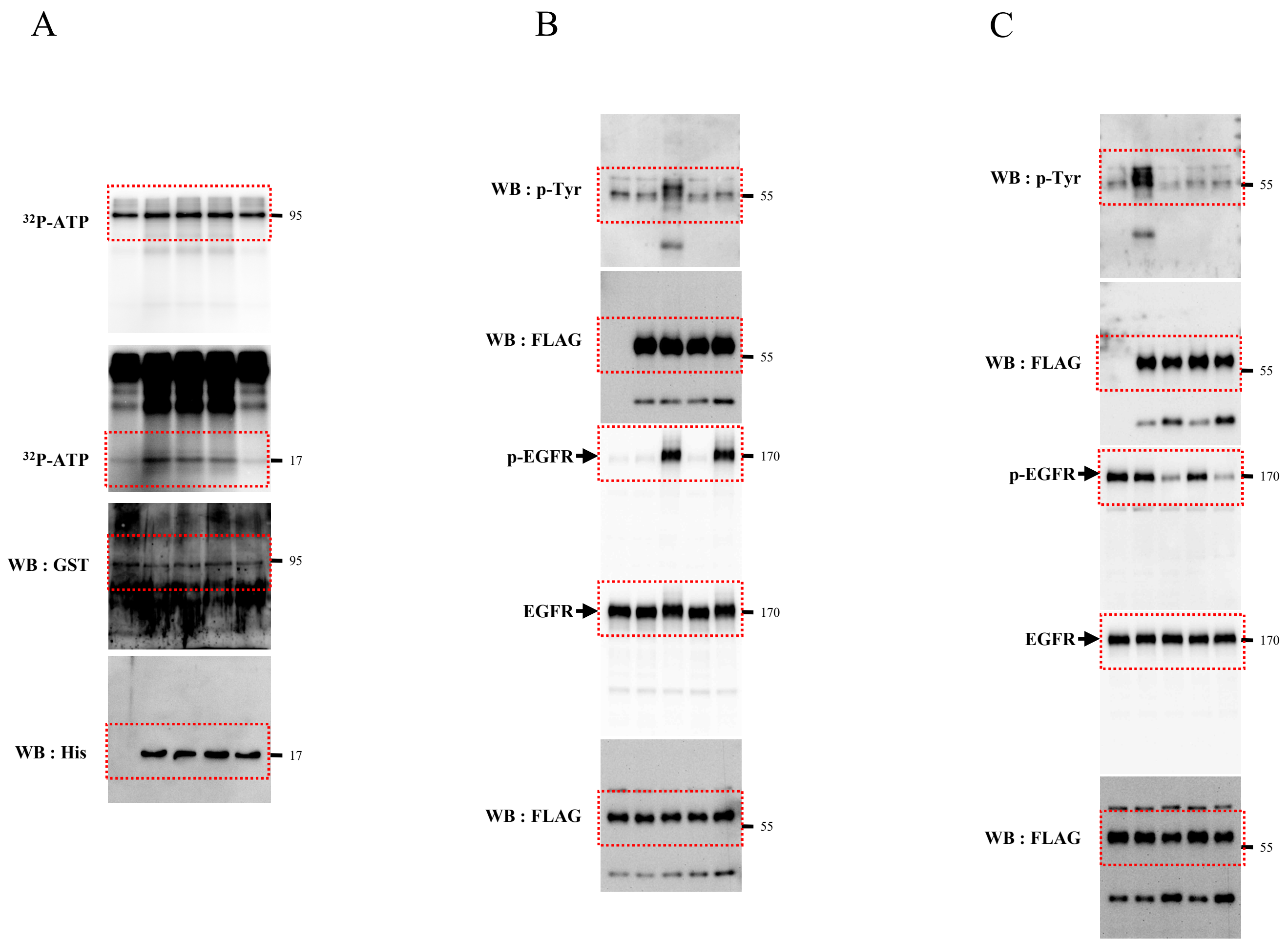
A
B
C
32P-ATP
95
32P-ATP
17
WB : GST
95
WB : His
17
WB : p-Tyr
55
WB : FLAG
55
p-EGFR
170
EGFR
170
WB : FLAG
55
WB : p-Tyr
55
WB : FLAG
55
p-EGFR
170
EGFR
170
WB : FLAG
55

Figure 6

E

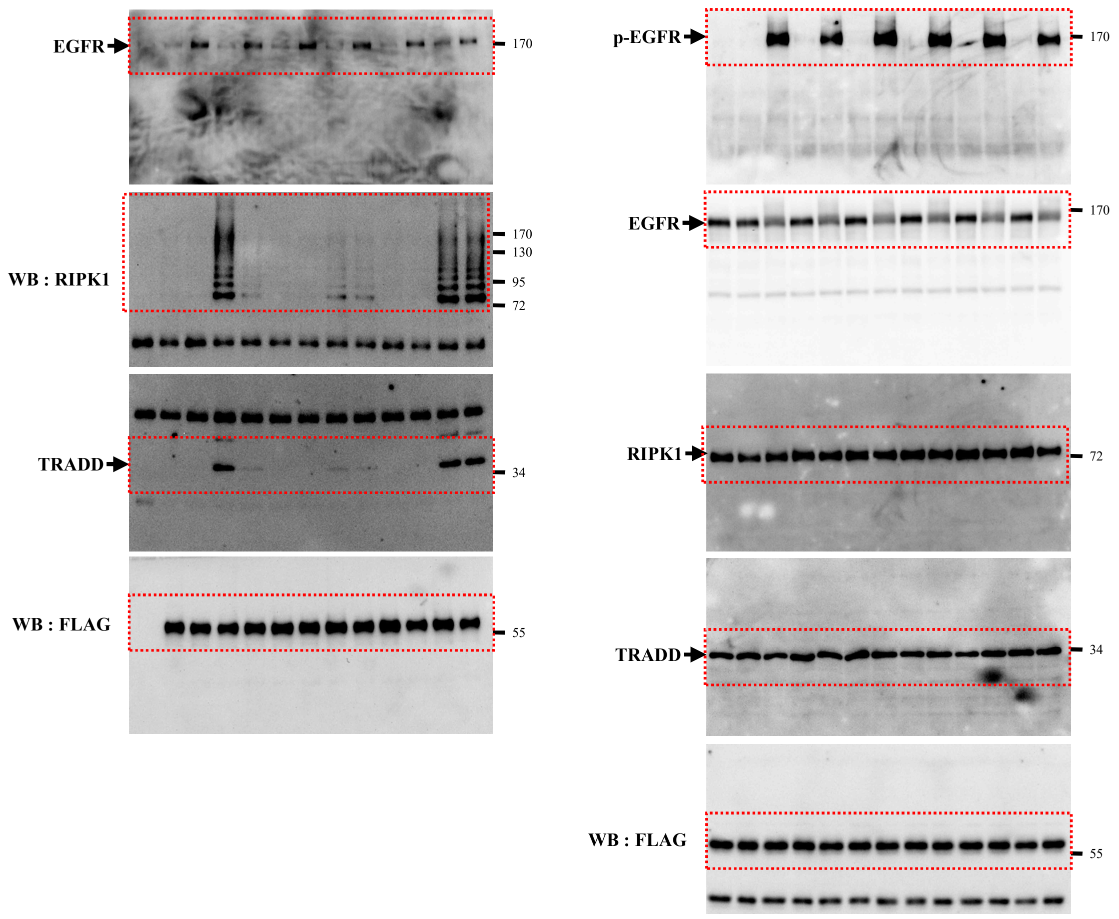

EGFR
170
WB : RIPK1
170
130
95
72
TRADD
34
WB : FLAG
55
p-EGFR
170
EGFR
170
RIPK1
72
TRADD
34
WB : FLAG
55

Figure 6

G

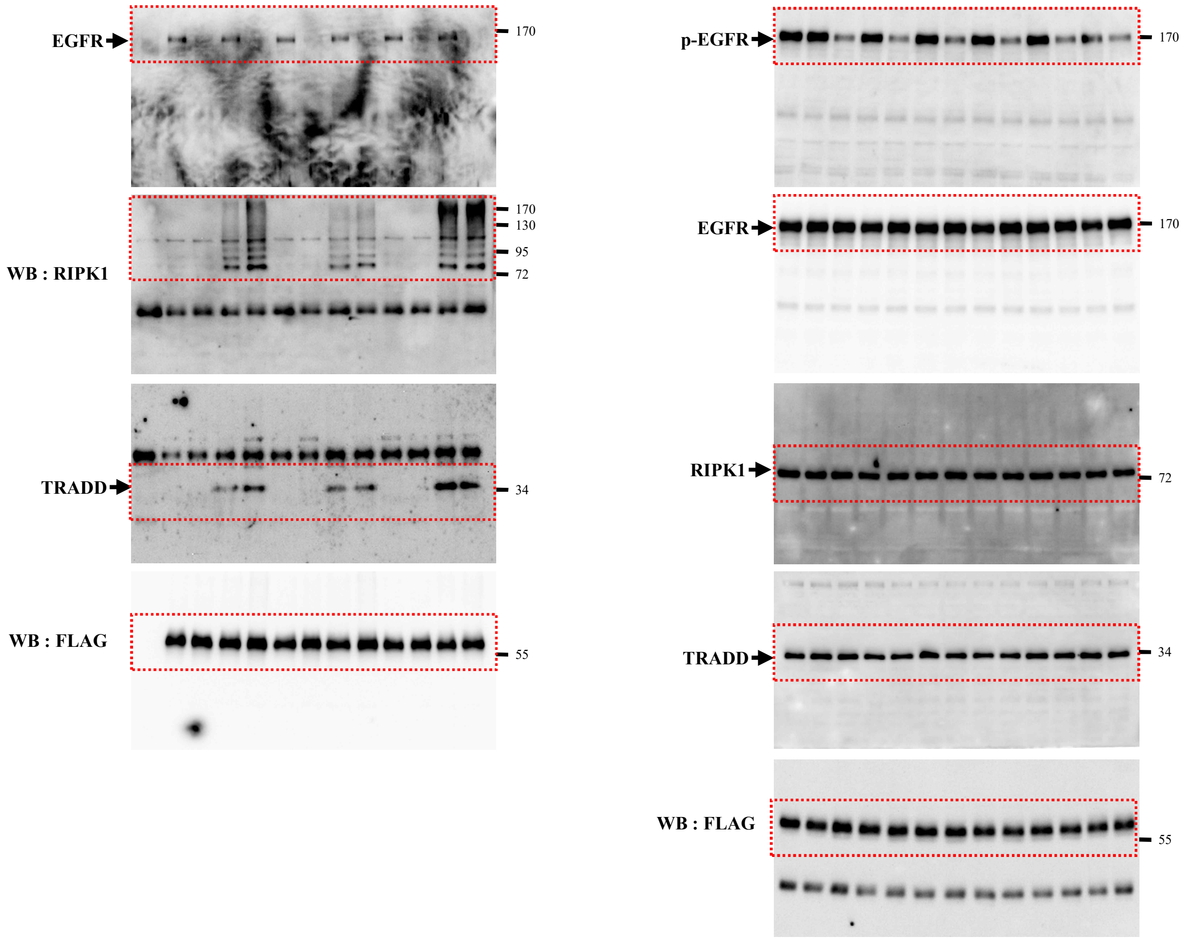
EGFR
170
WB : RIPK1
170
130
95
72
TRADD
34
WB : FLAG
55
p-EGFR
170
EGFR
170
RIPK1
72
TRADD
34
WB : FLAG
55

Figure 7

G

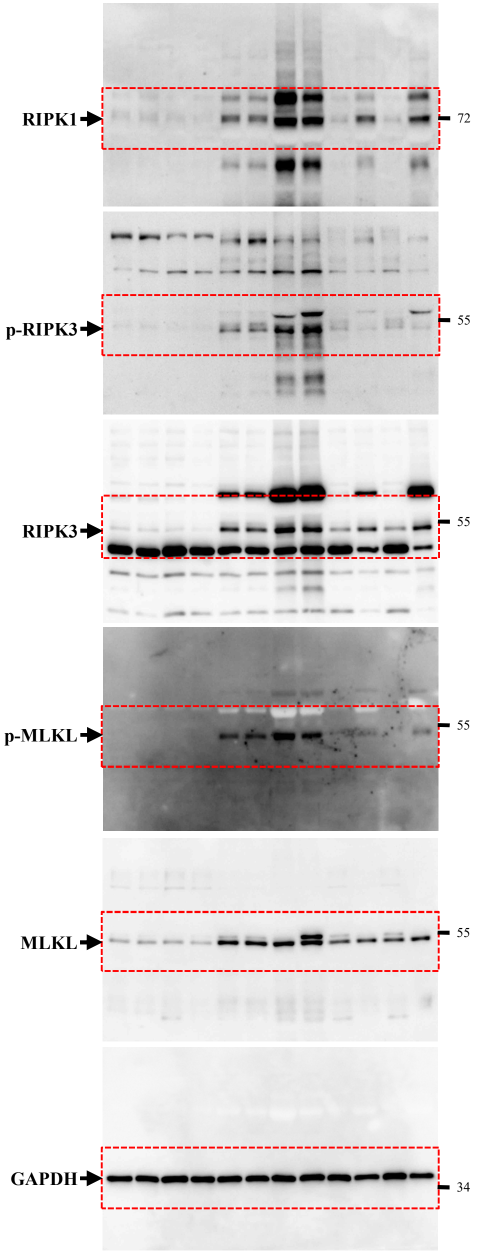

Supplementary Figure 1

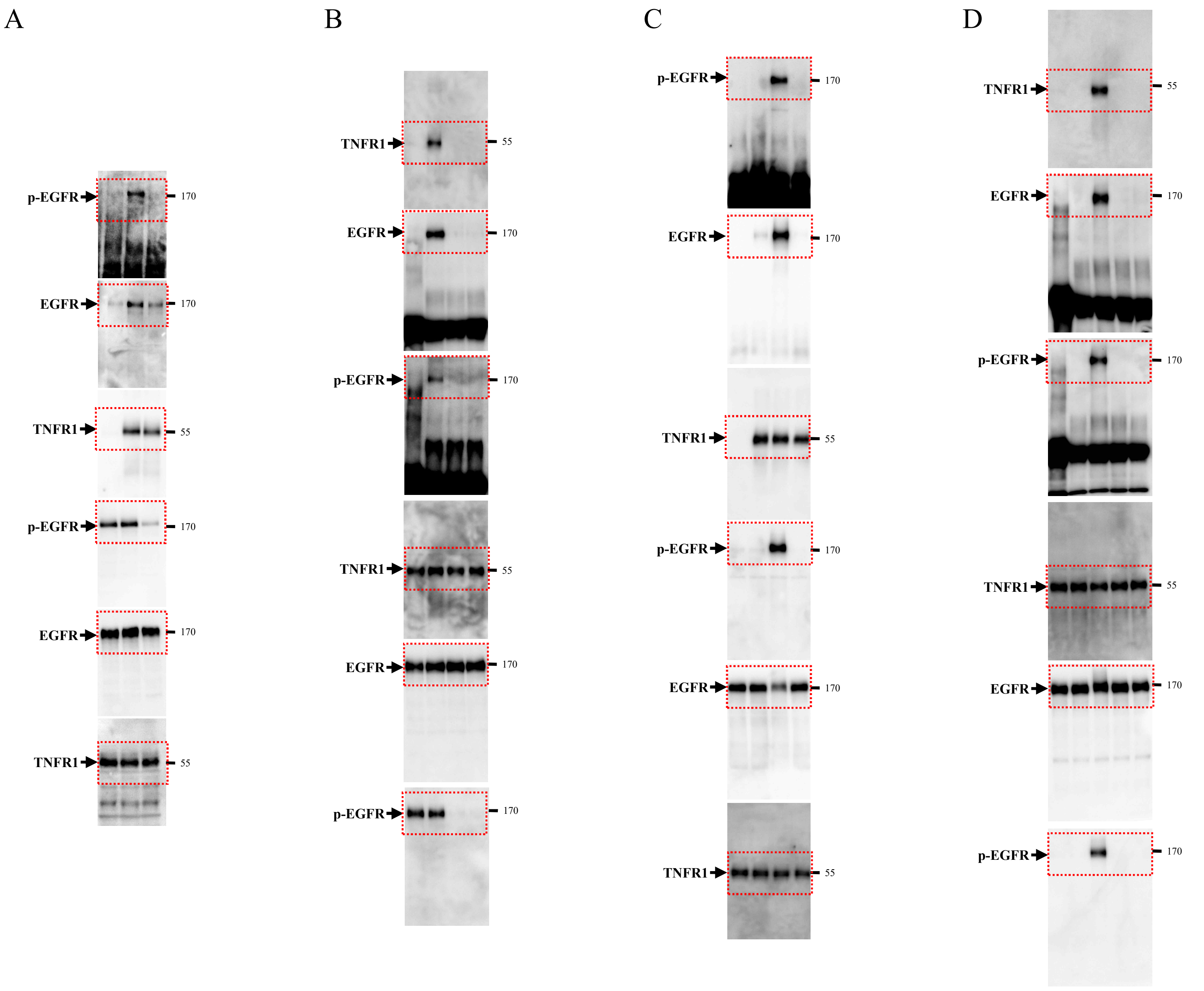

Supplementary Figure 2

A

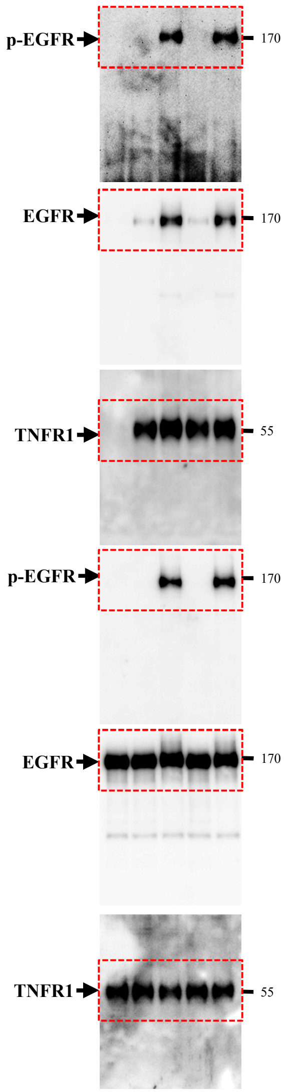

Supplementary Figure 3

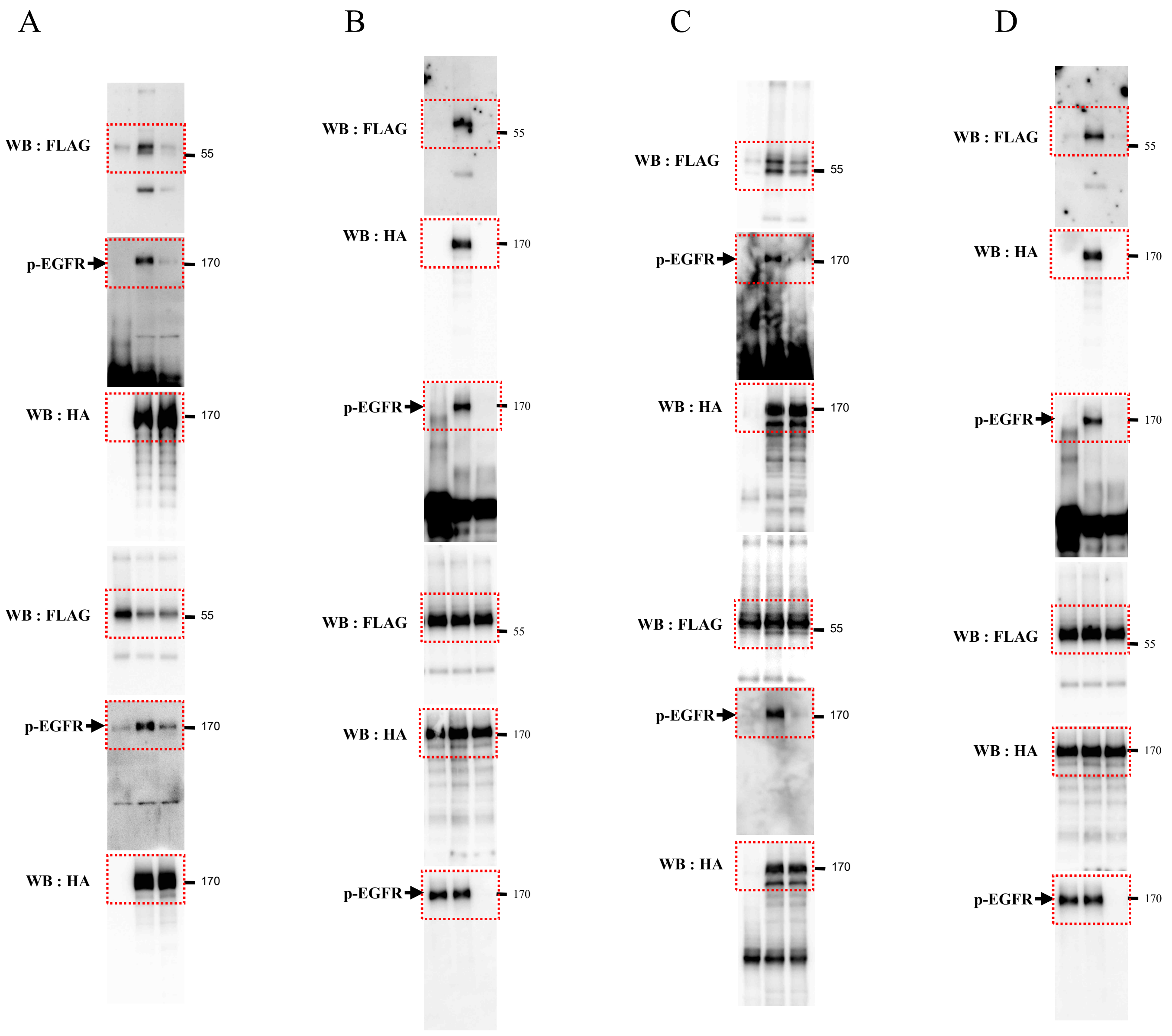

Supplementary Figure 4

B

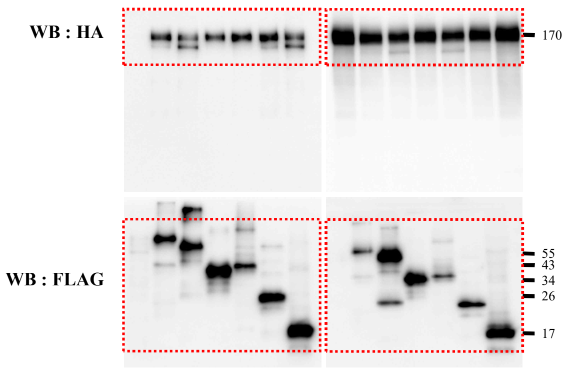

D

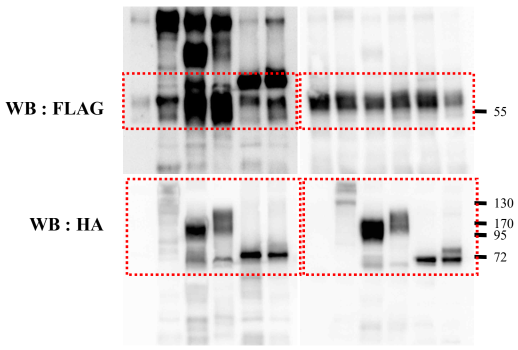

Supplementary Figure 5

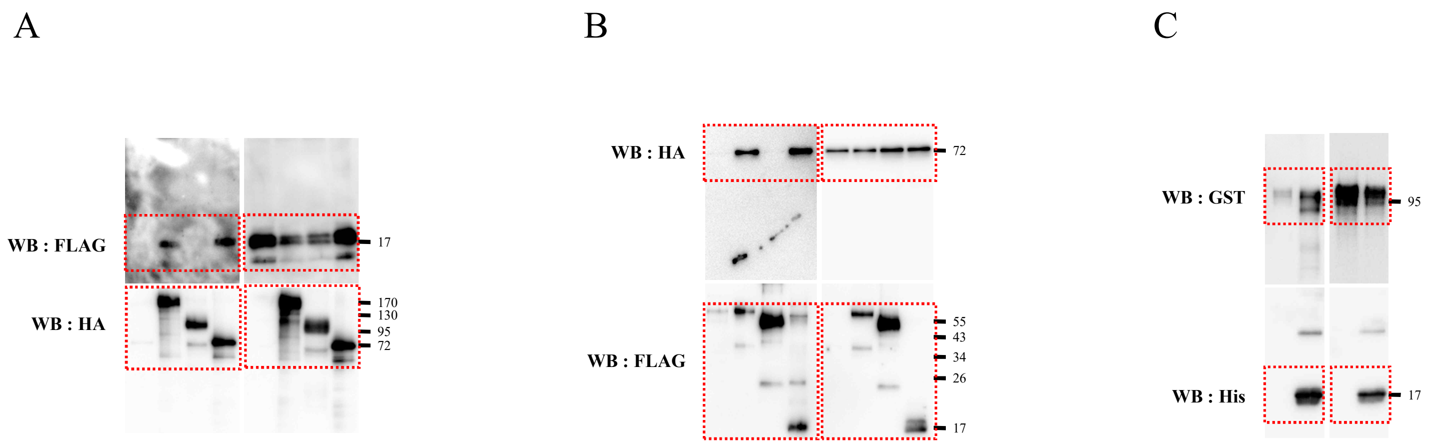

Supplementary Figure 6

A

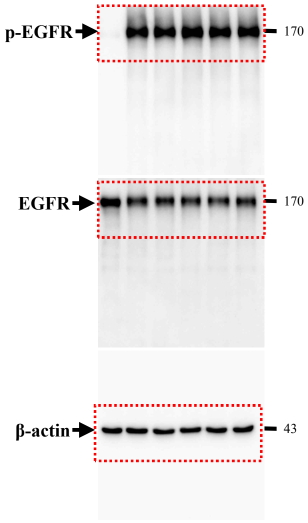

B

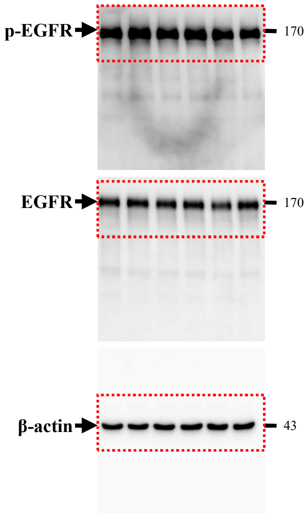

Supplementary Figure 7

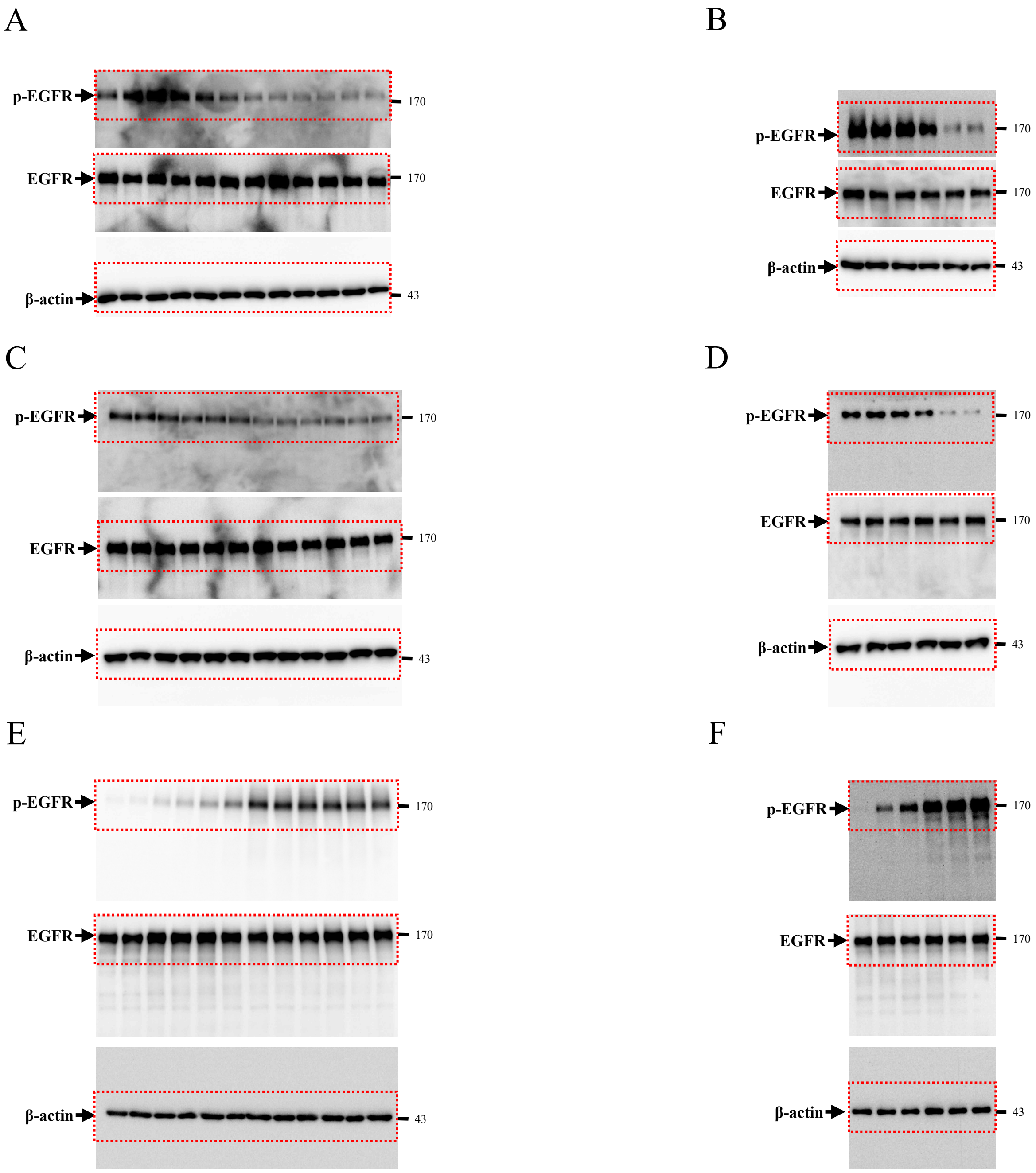

Supplementary Figure 9

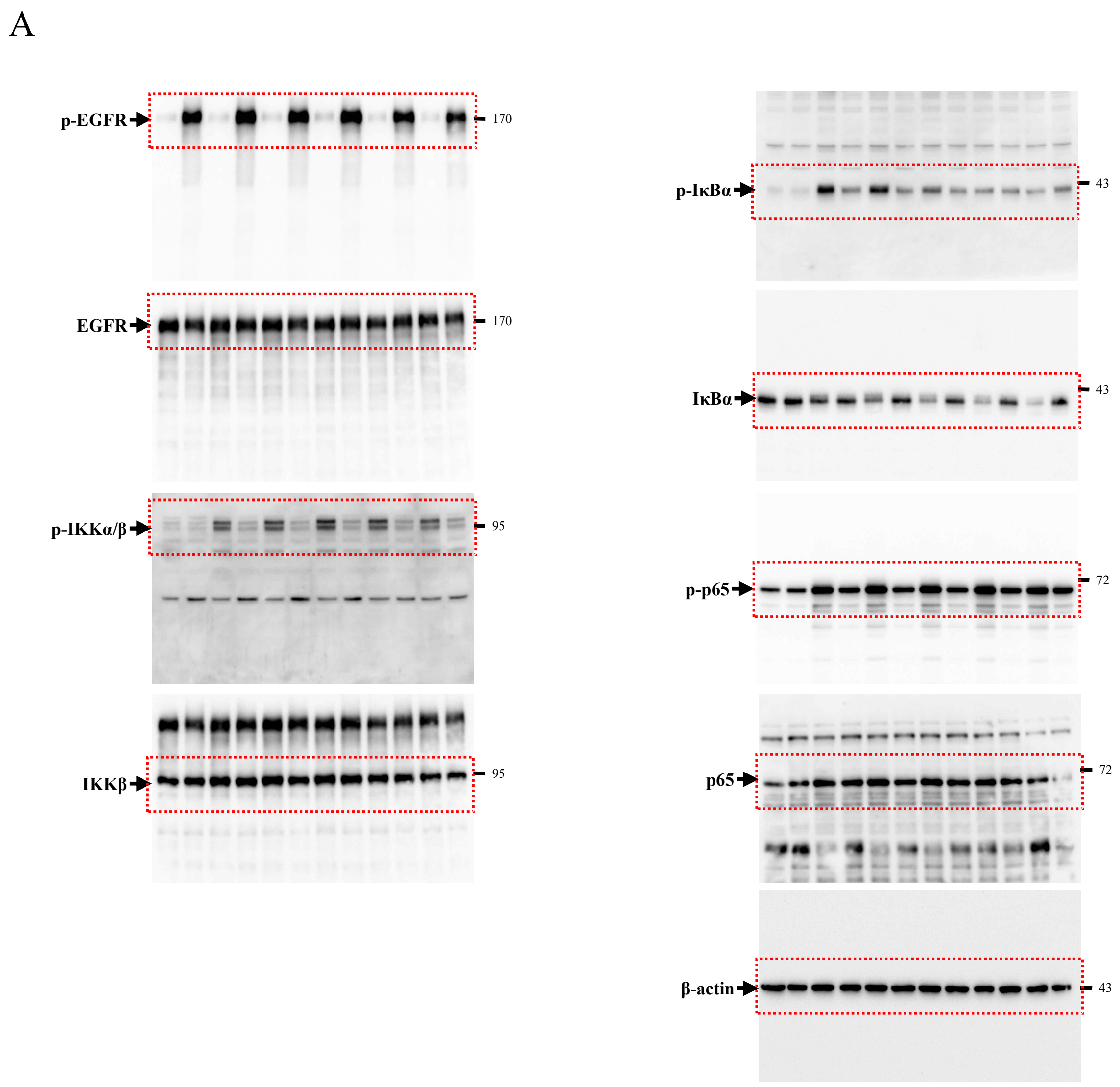

Supplementary Figure 10

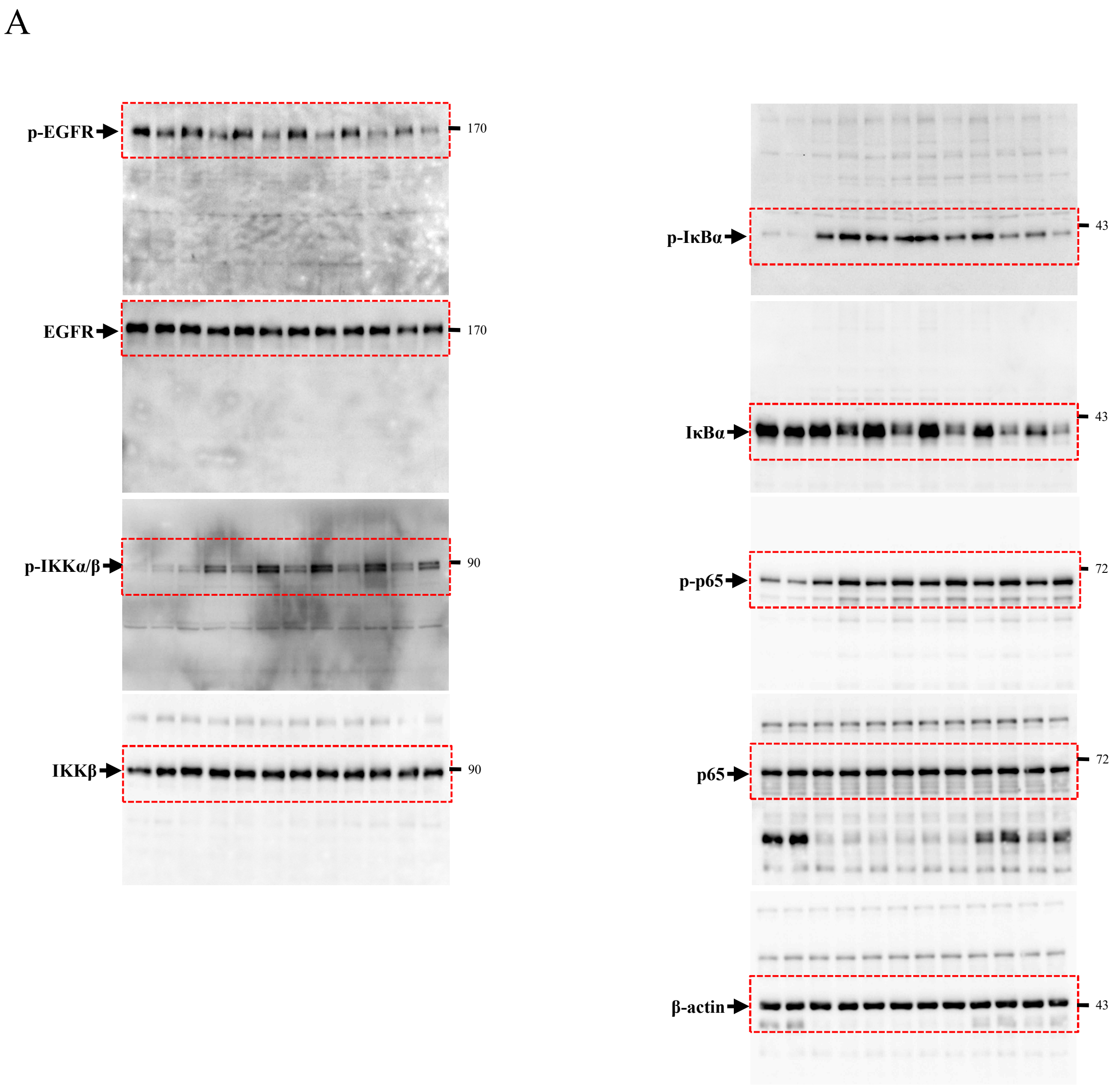

Supplementary Figure 10

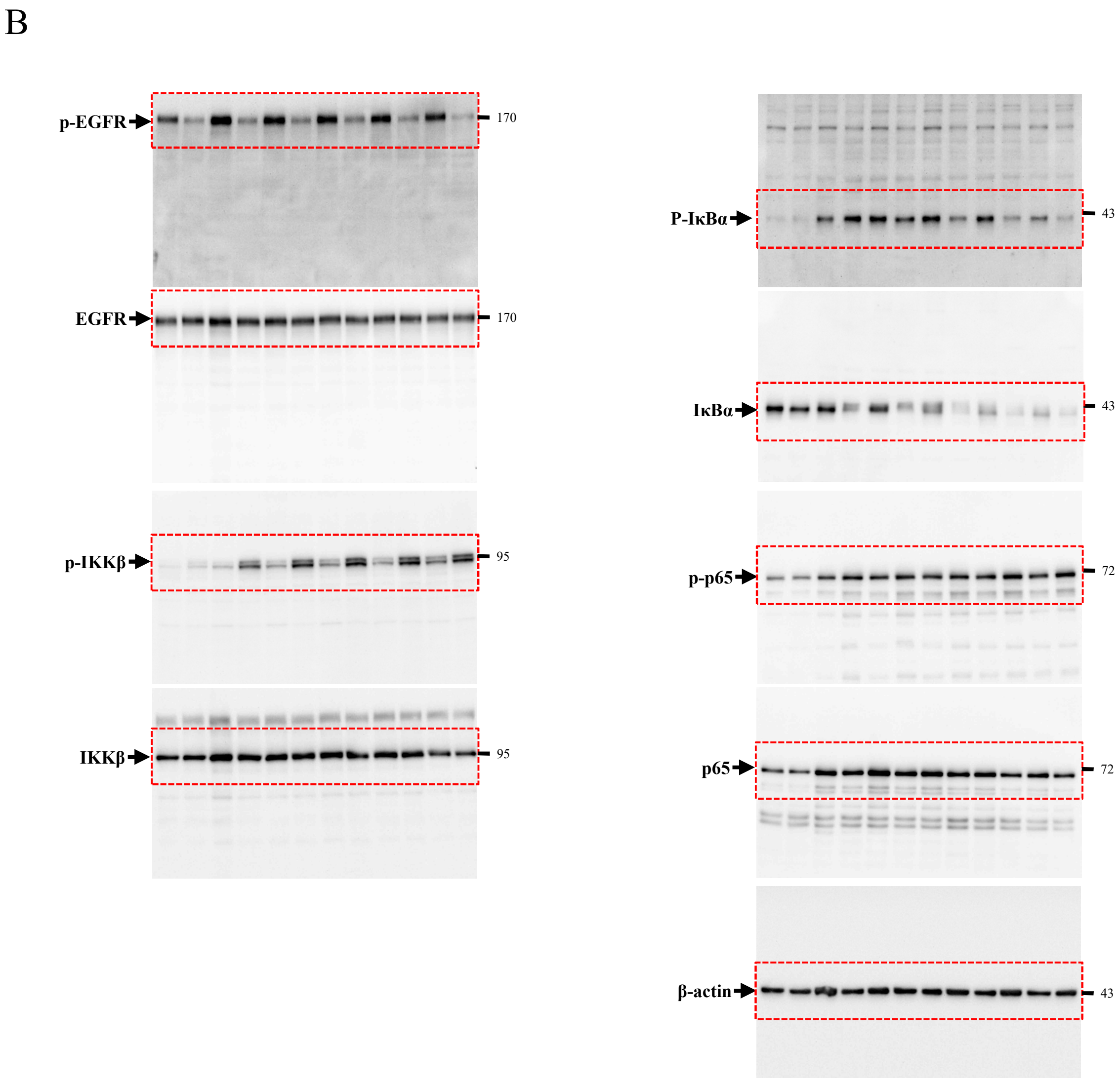

Supplementary Figure 10

C

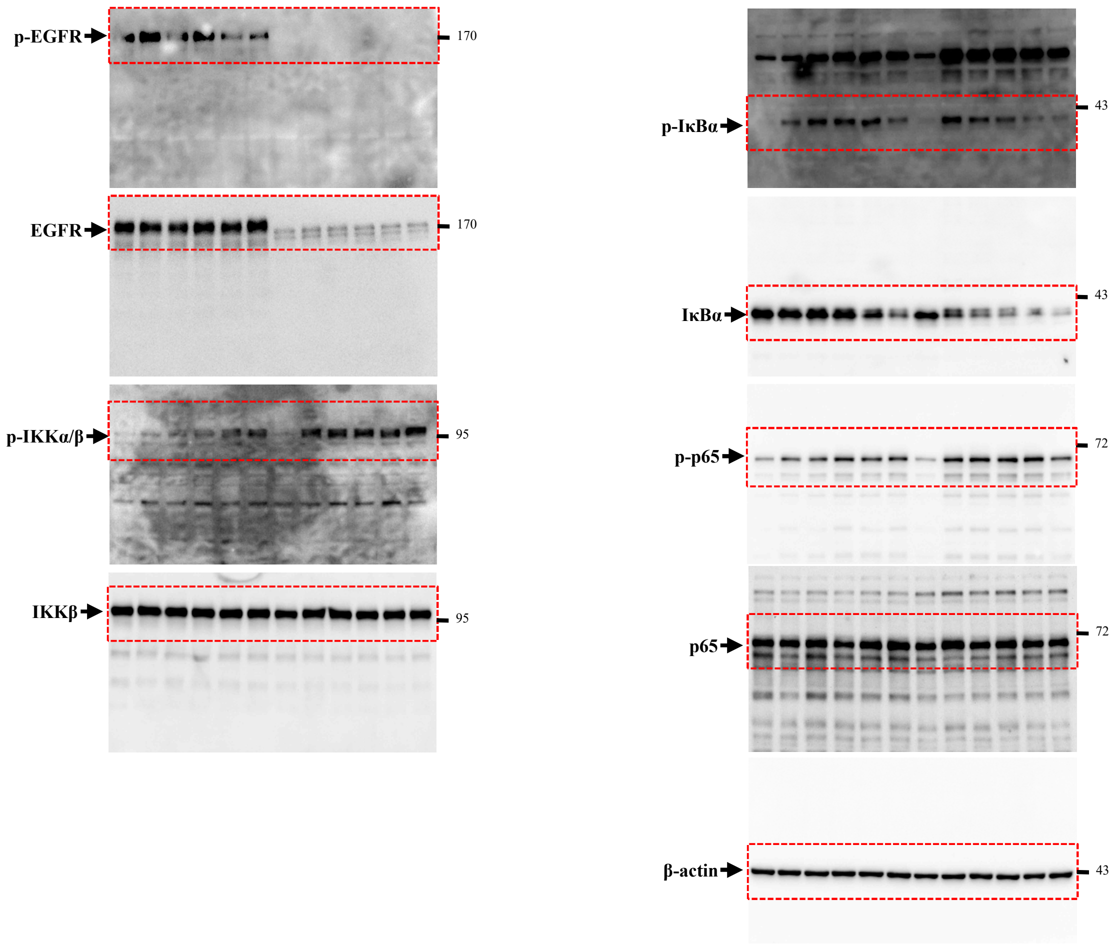

Supplementary Figure 10

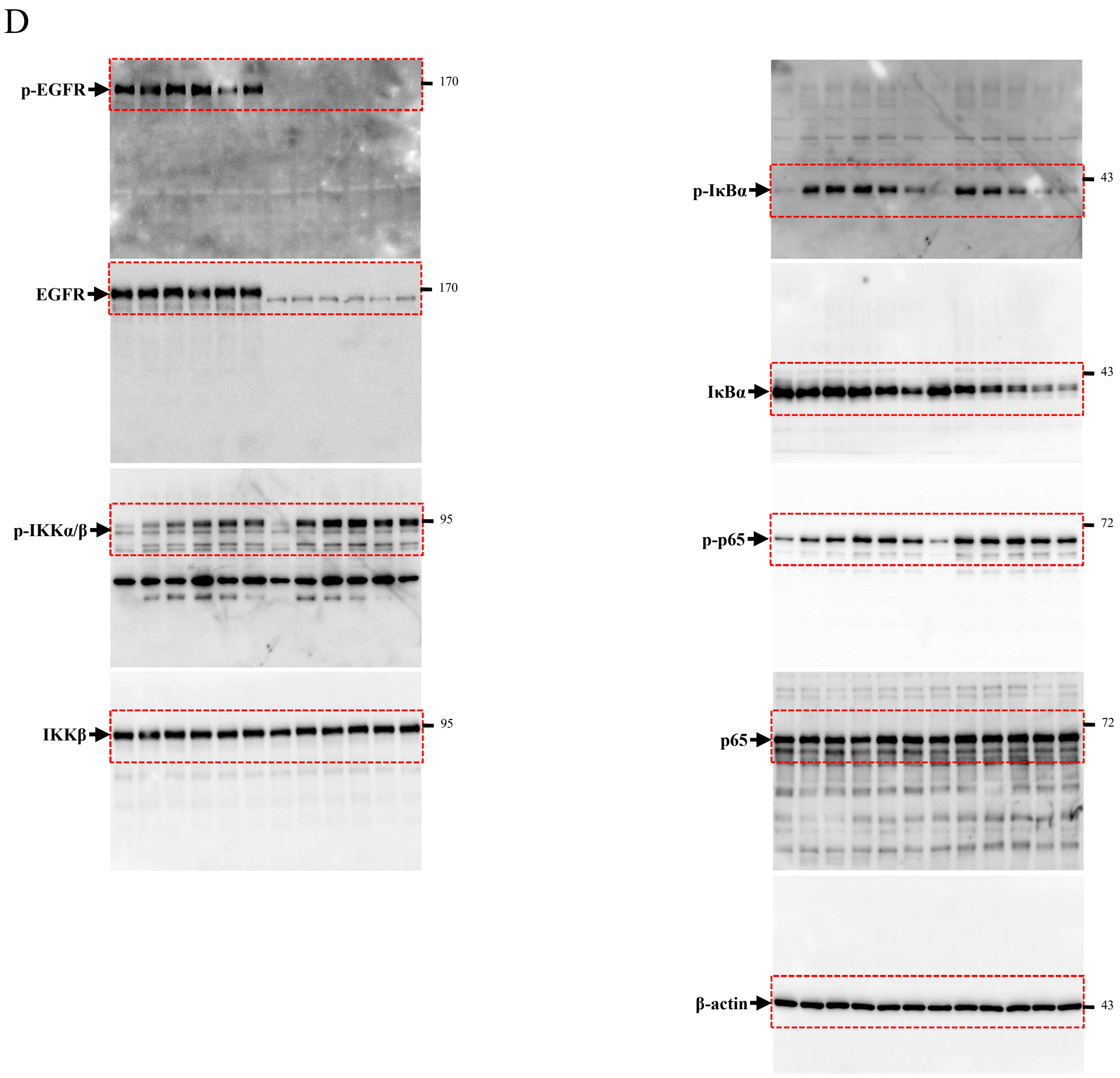

Supplementary Figure 11

B

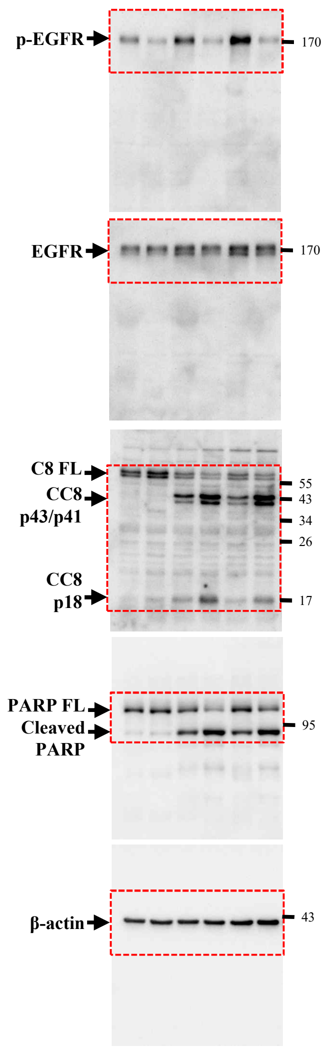

C

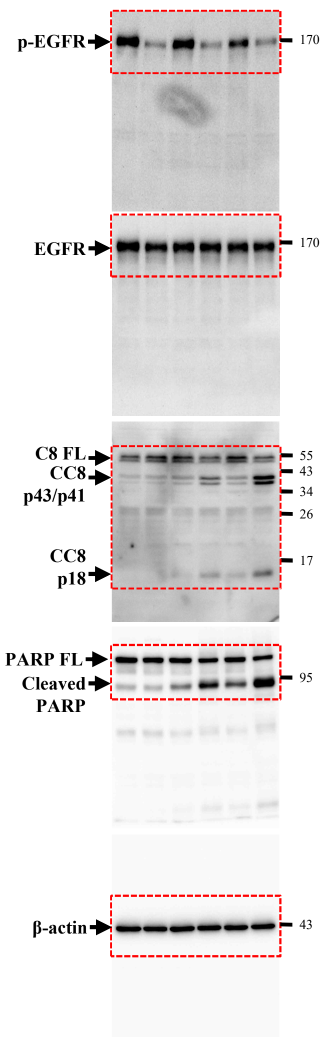

Supplementary Figure 11

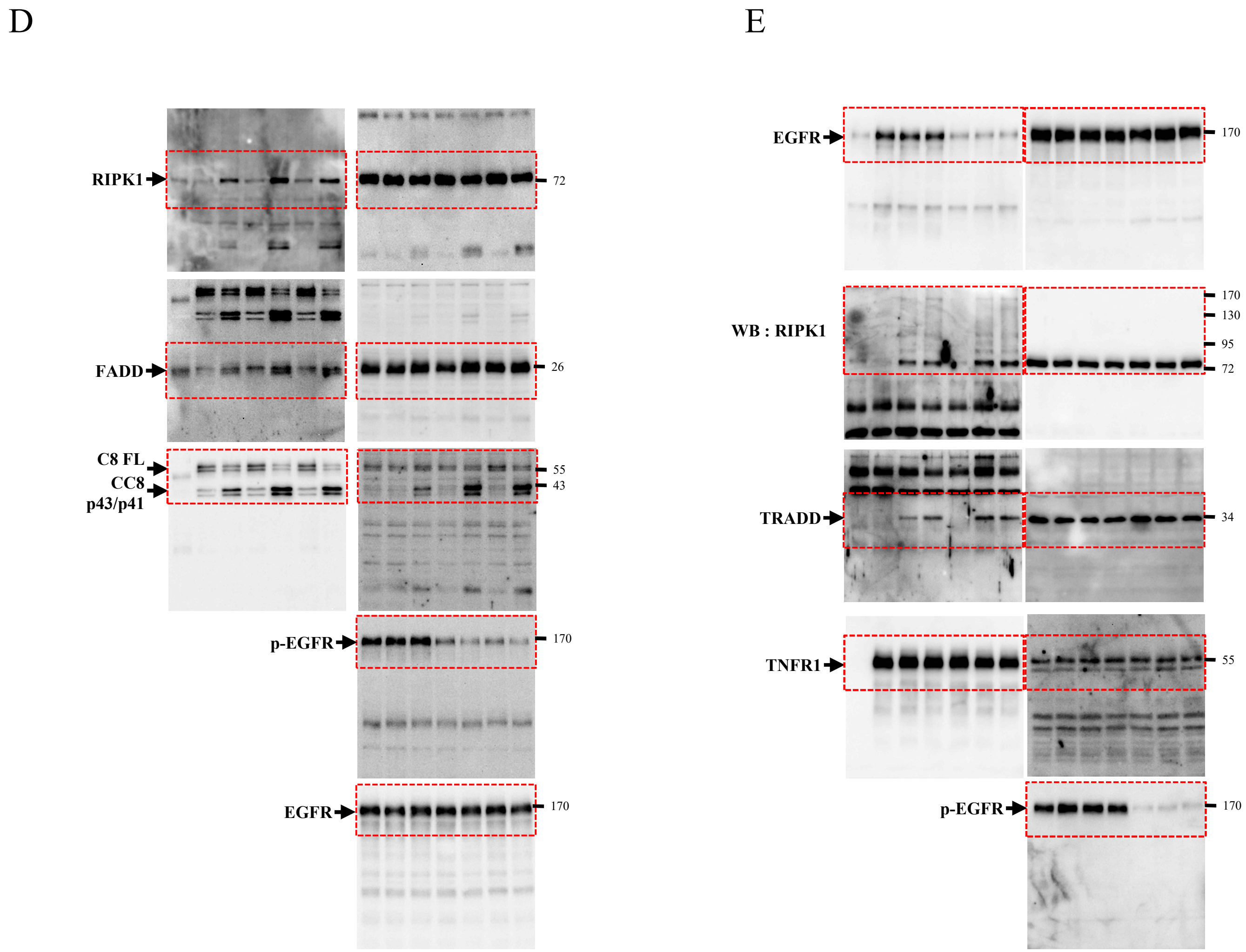

Supplementary Figure 12

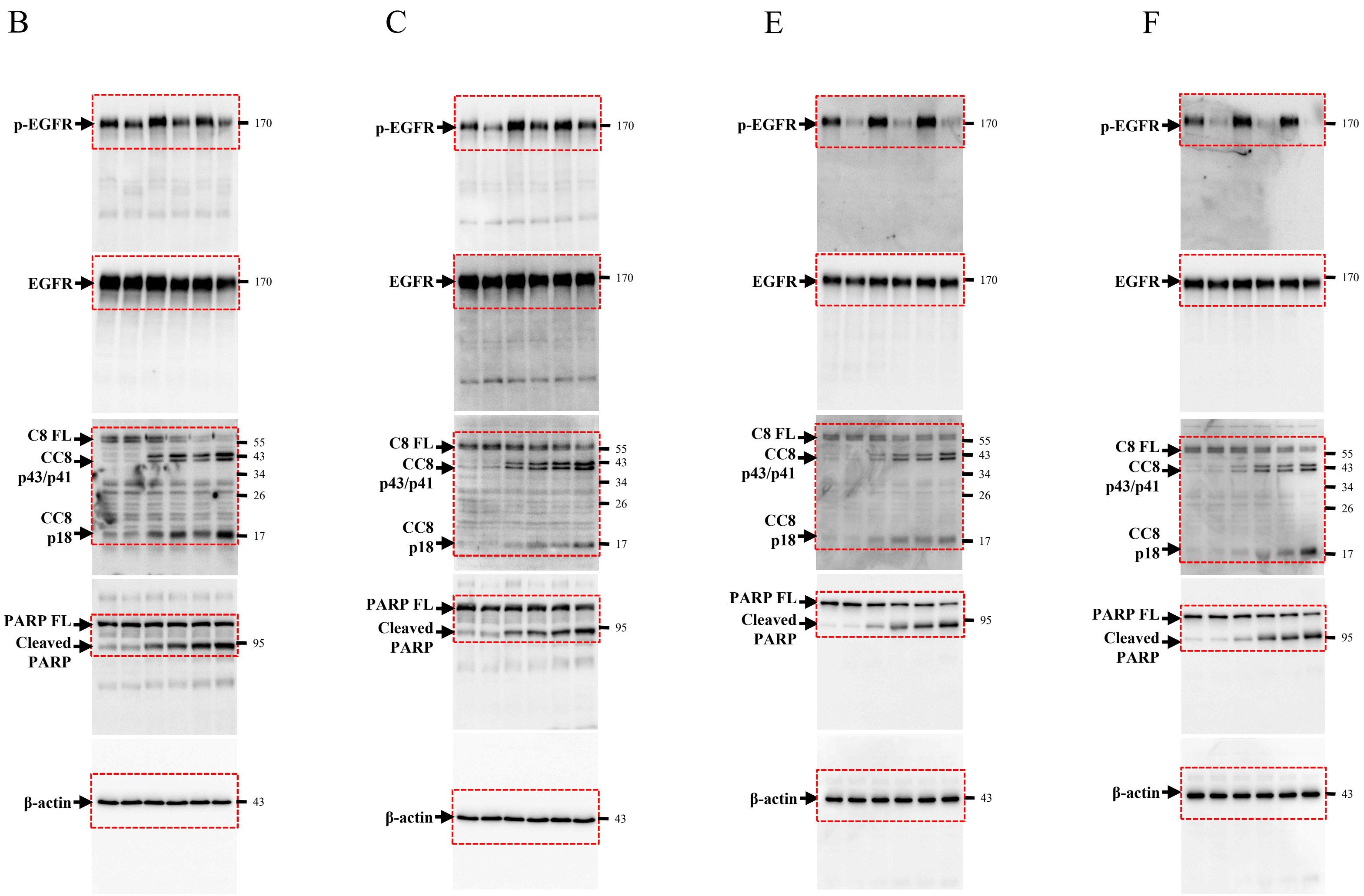

Supplementary Figure 13

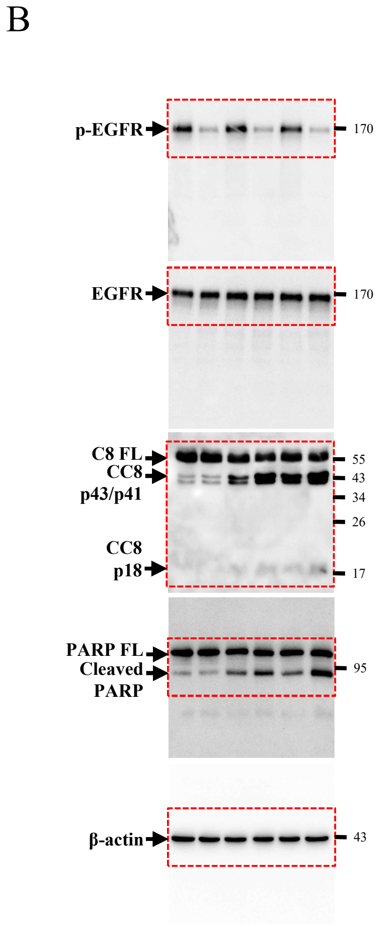

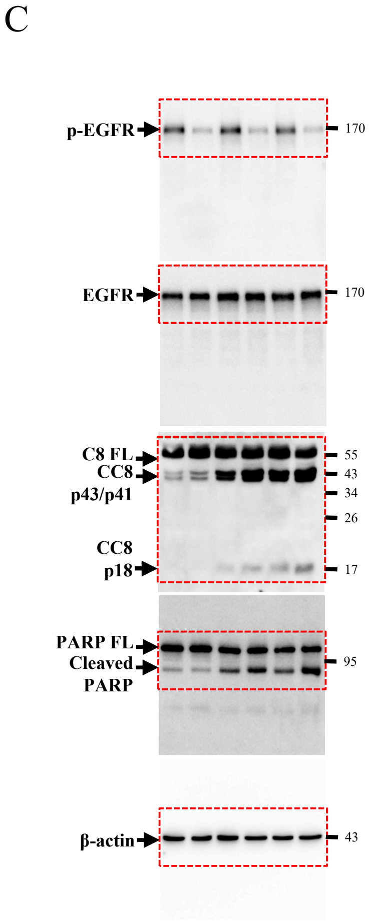

Supplementary Figure 13

D

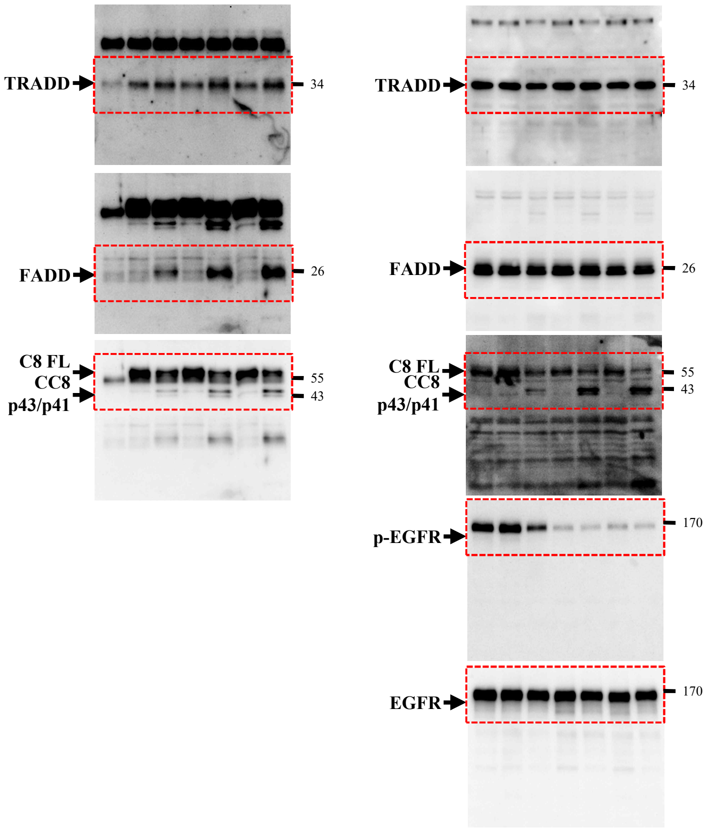

E

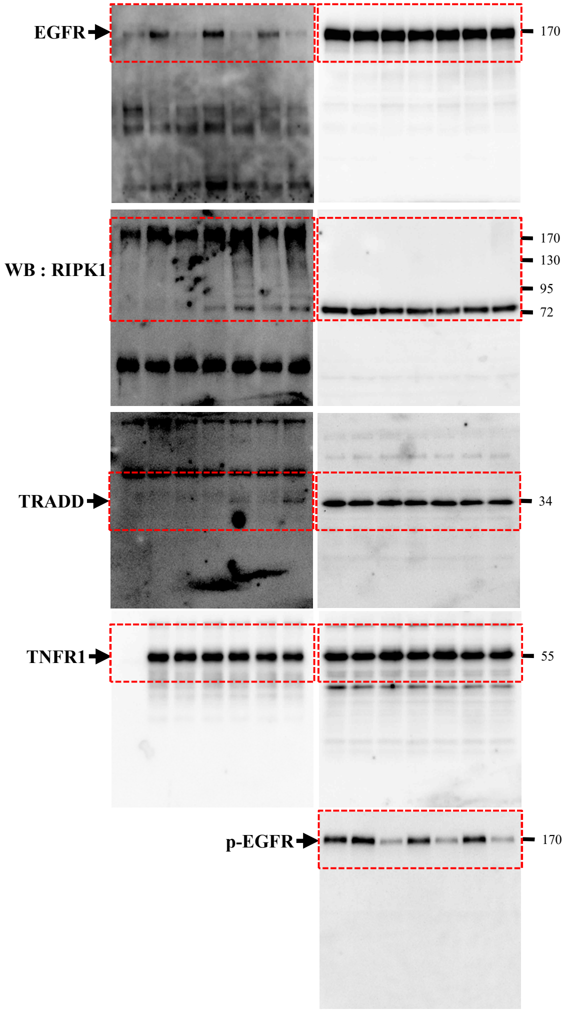

Supplementary Figure 14

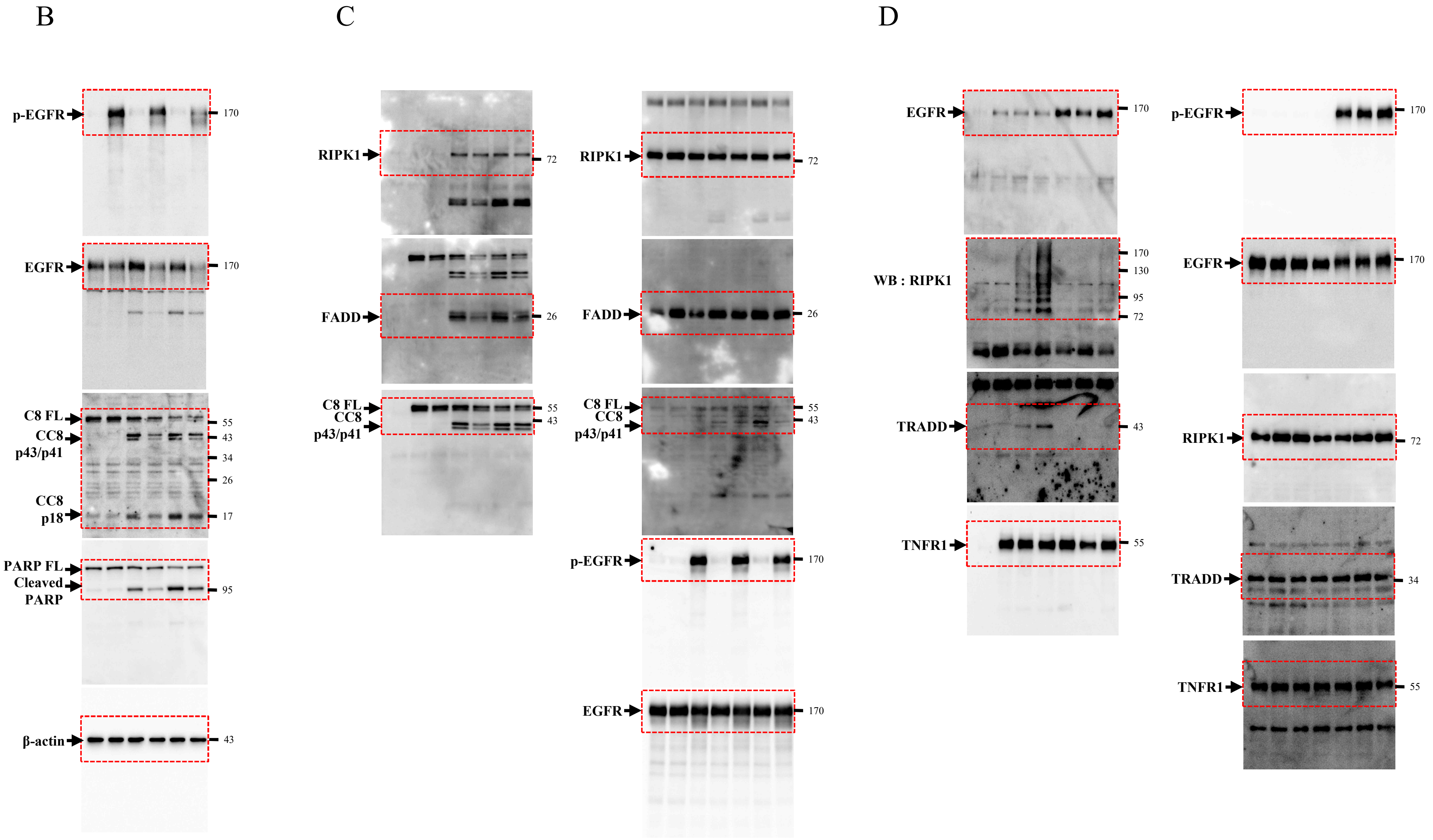

Supplementary Figure 15

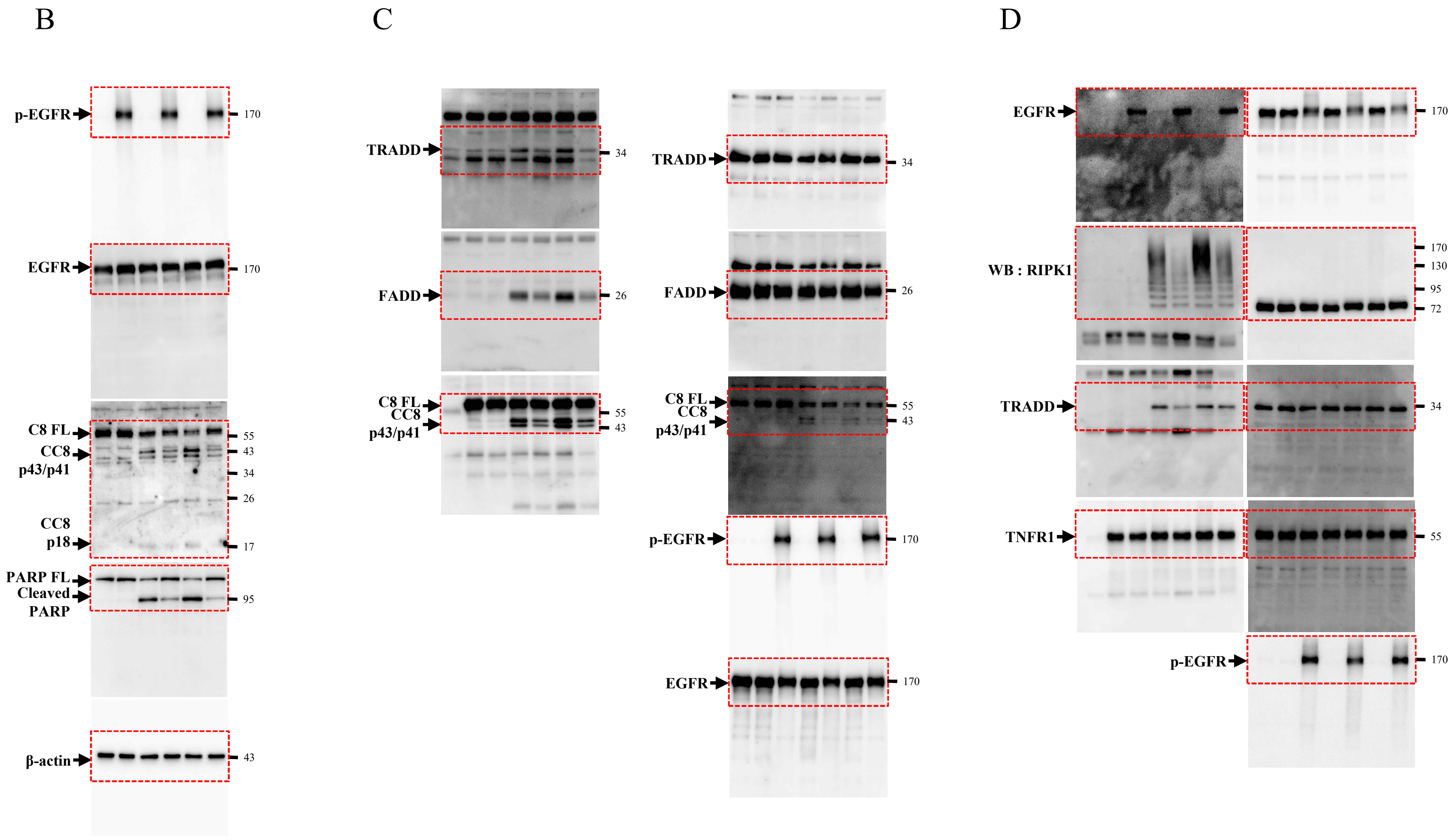

Supplementary Figure 17

B

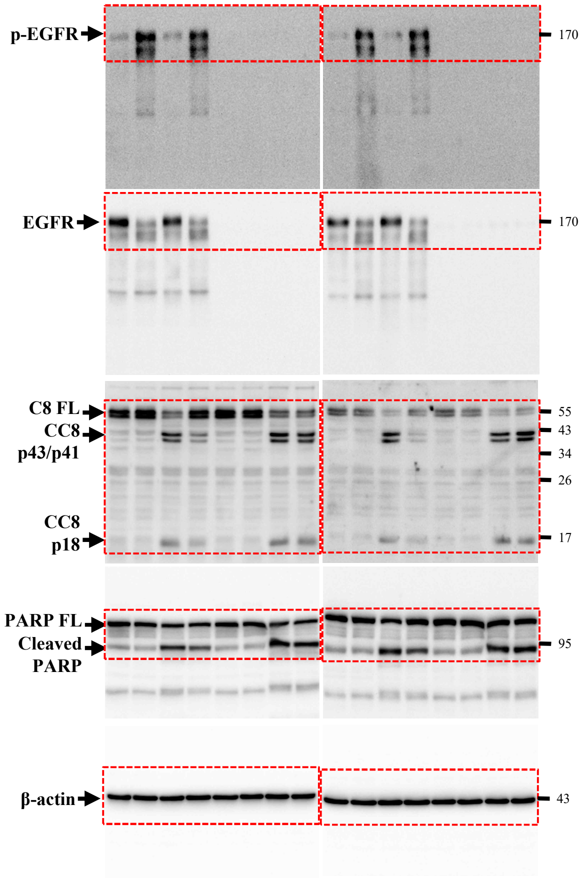

Supplementary Figure 18

A

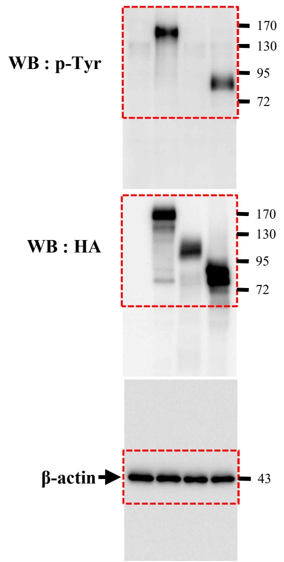

Supplementary Figure 19

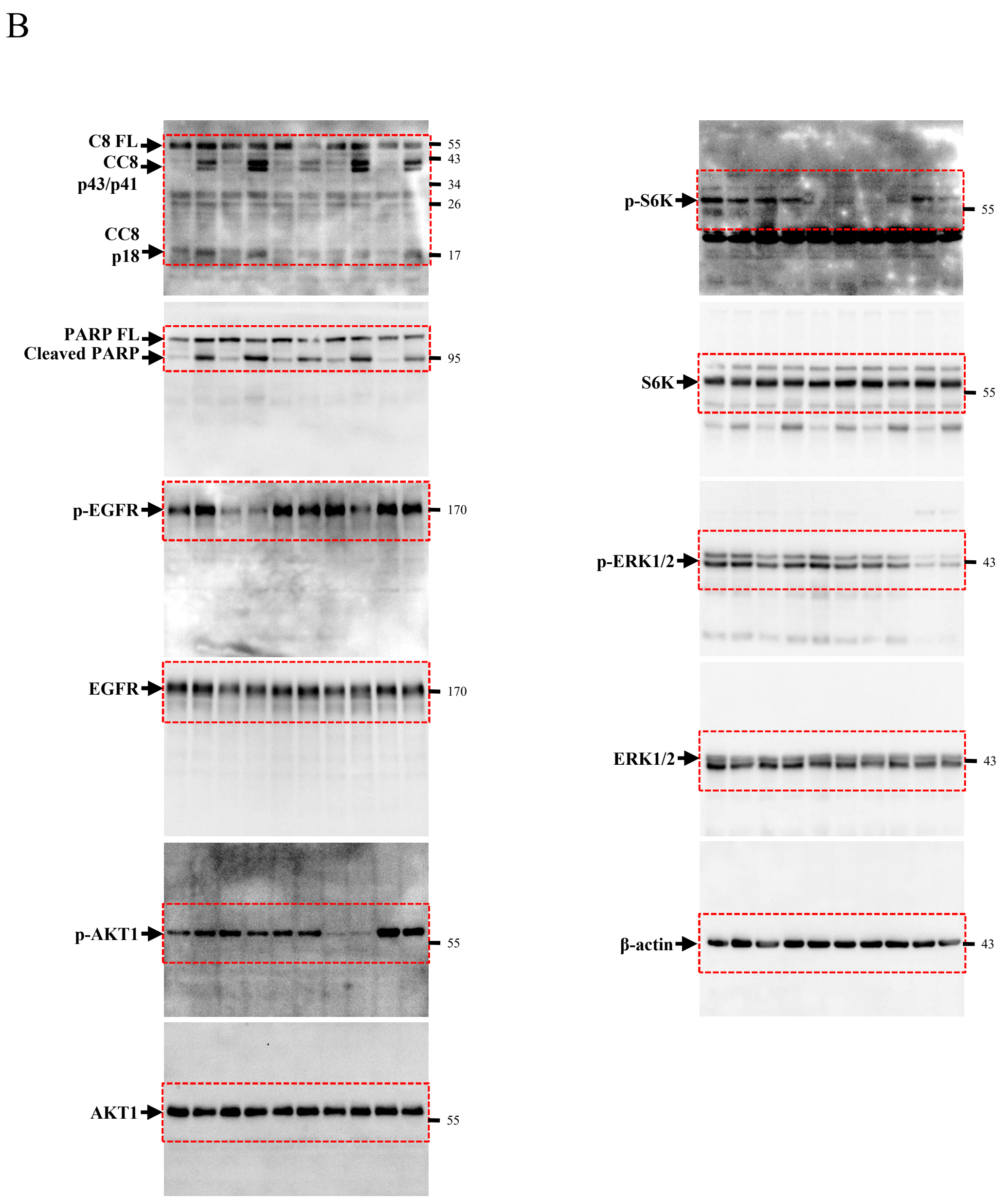

Supplementary Figure 19

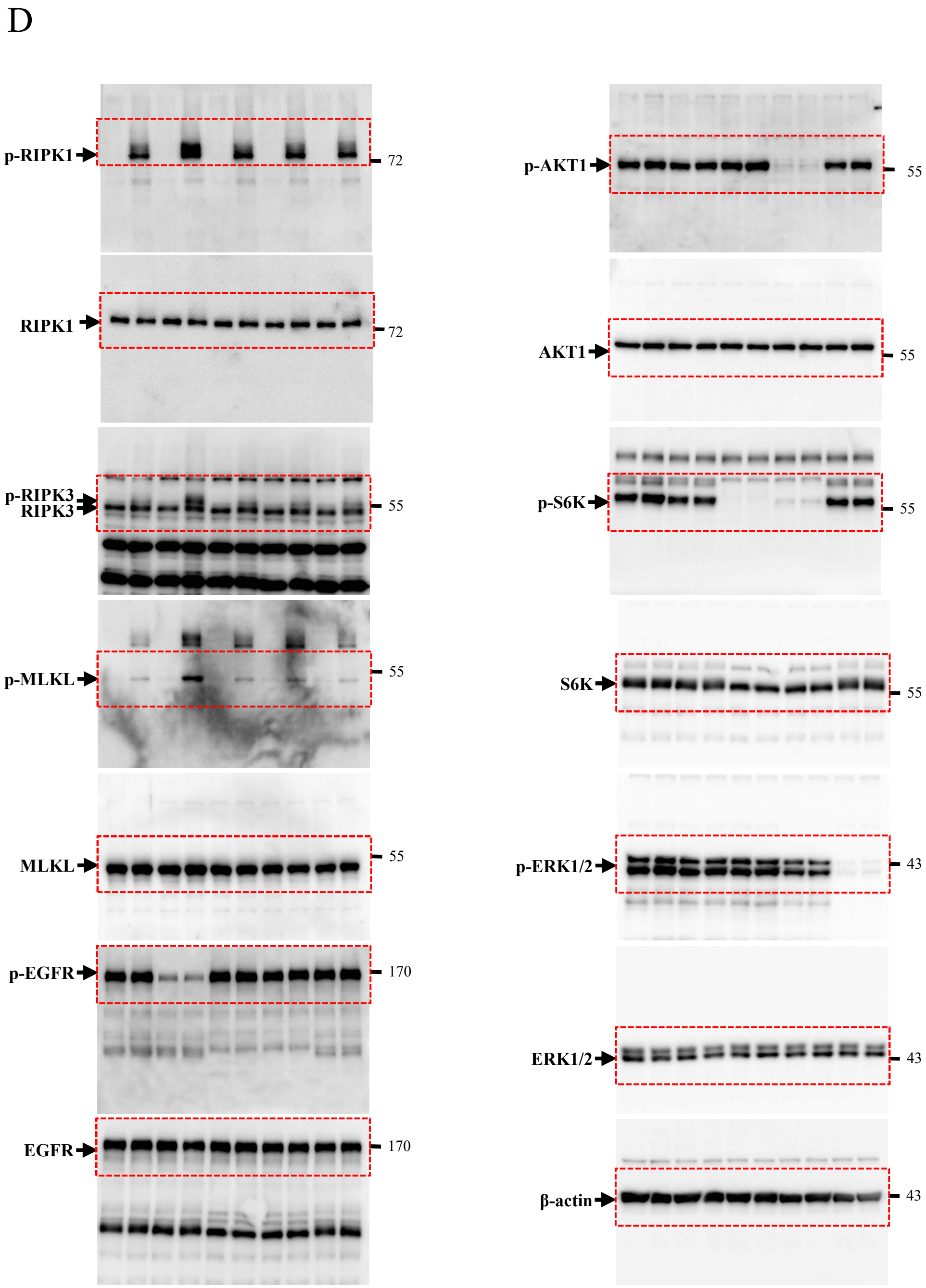

Supplementary Figure 19

F

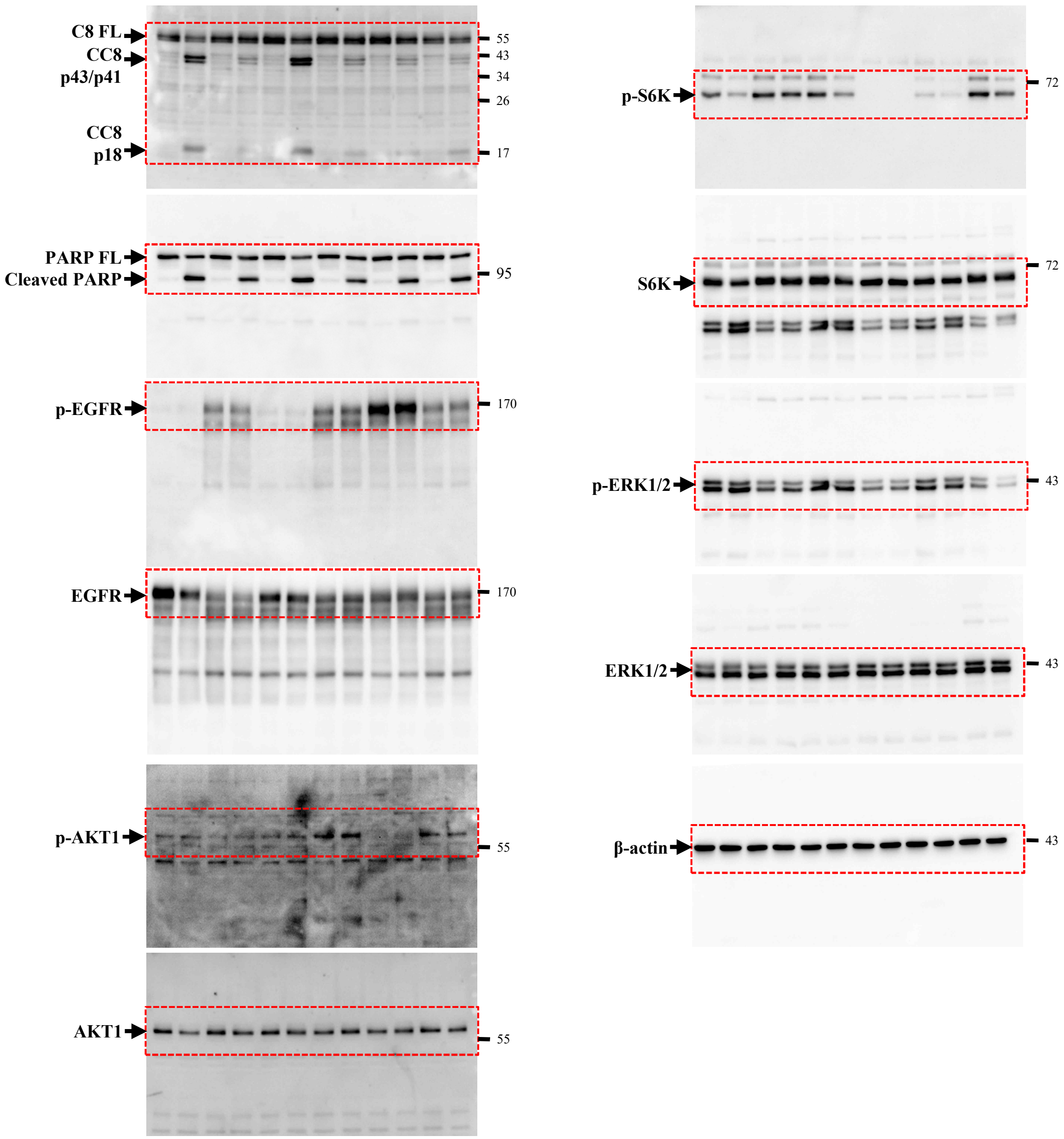

Supplementary Figure 20

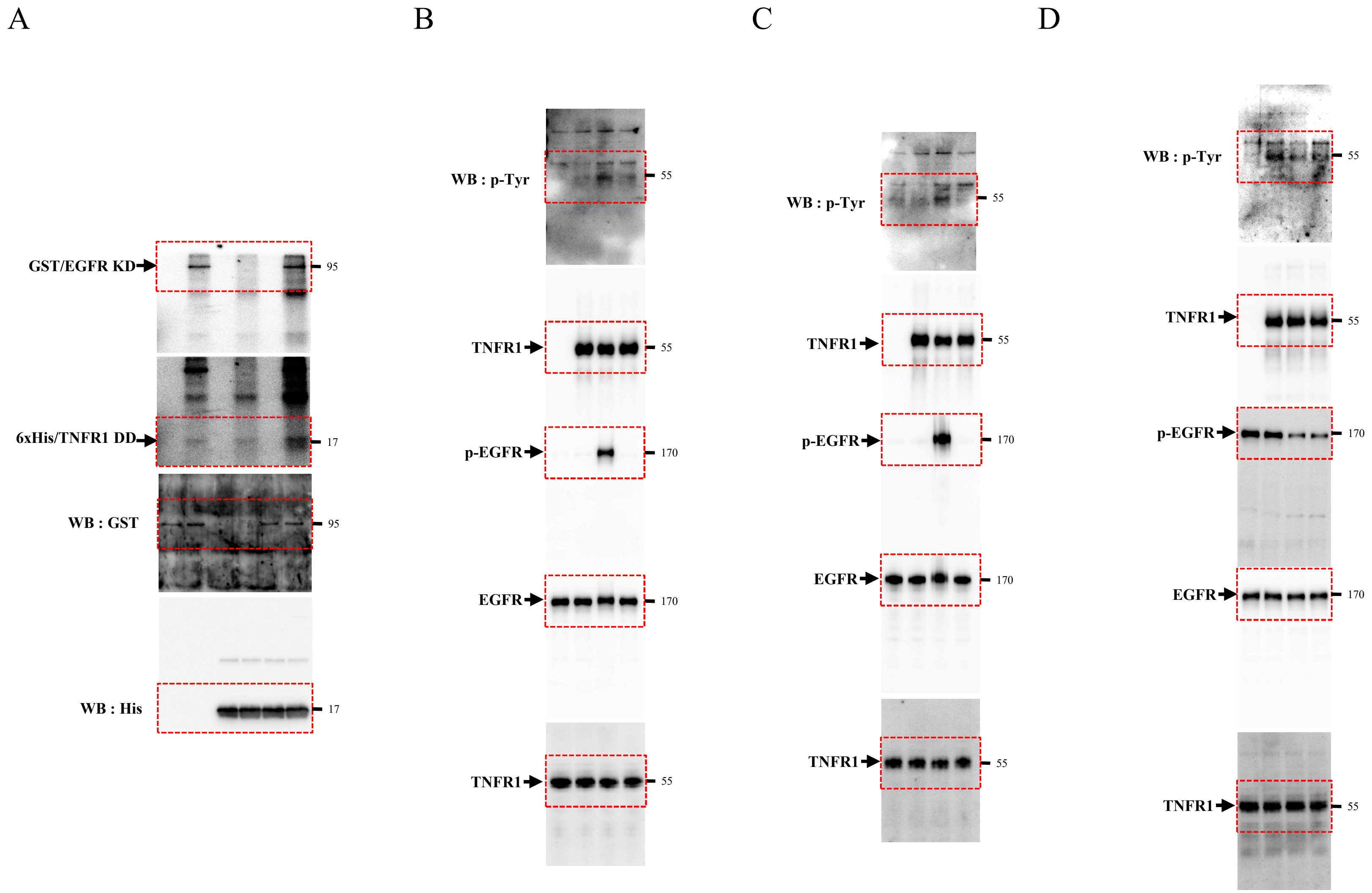

Supplementary Figure 21

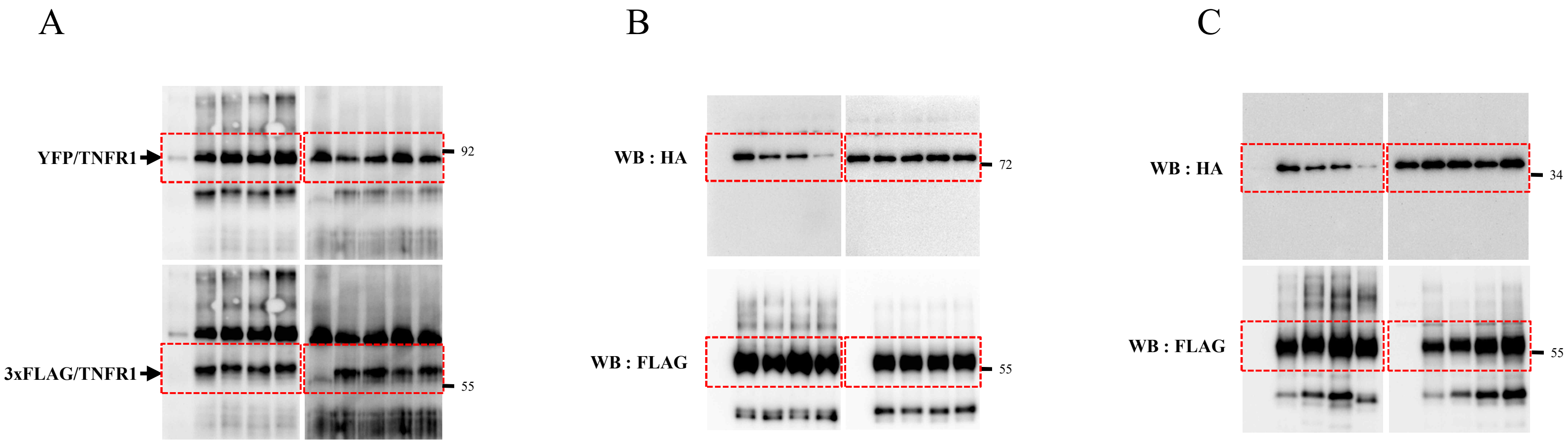

Supplementary Figure 22

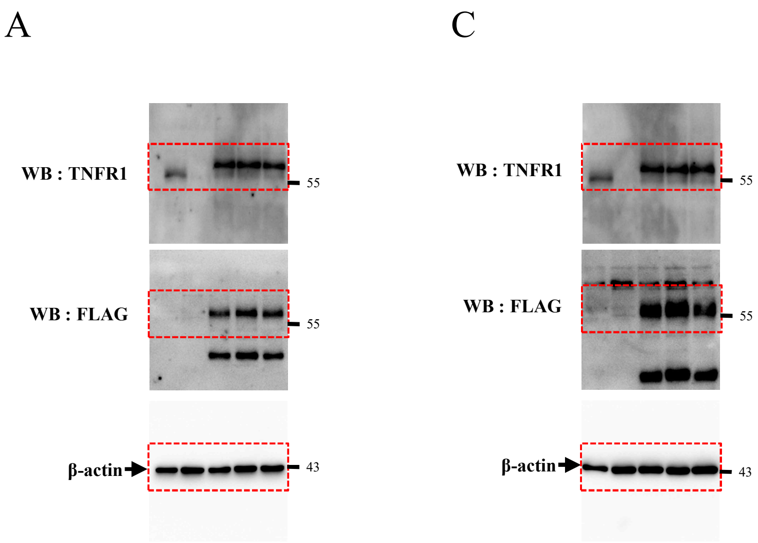

Supplementary Figure 23

A

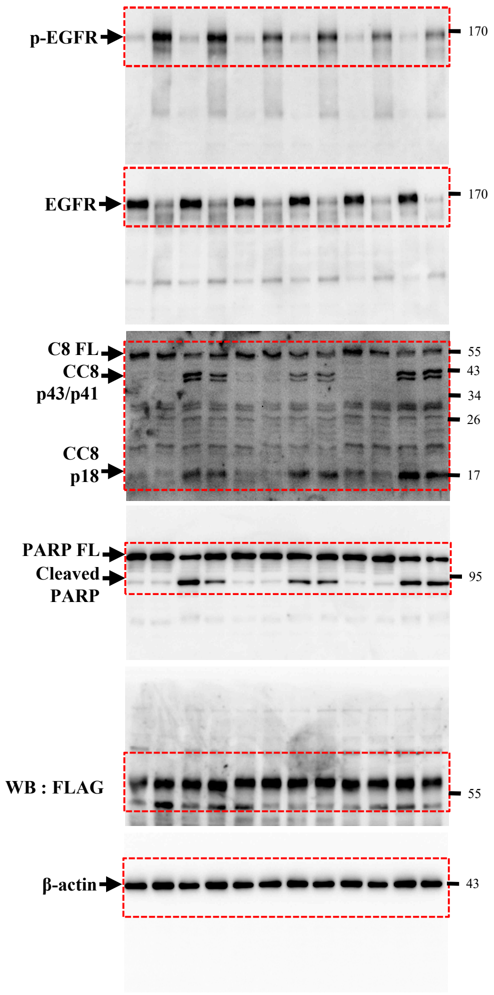

Supplementary Figure 23

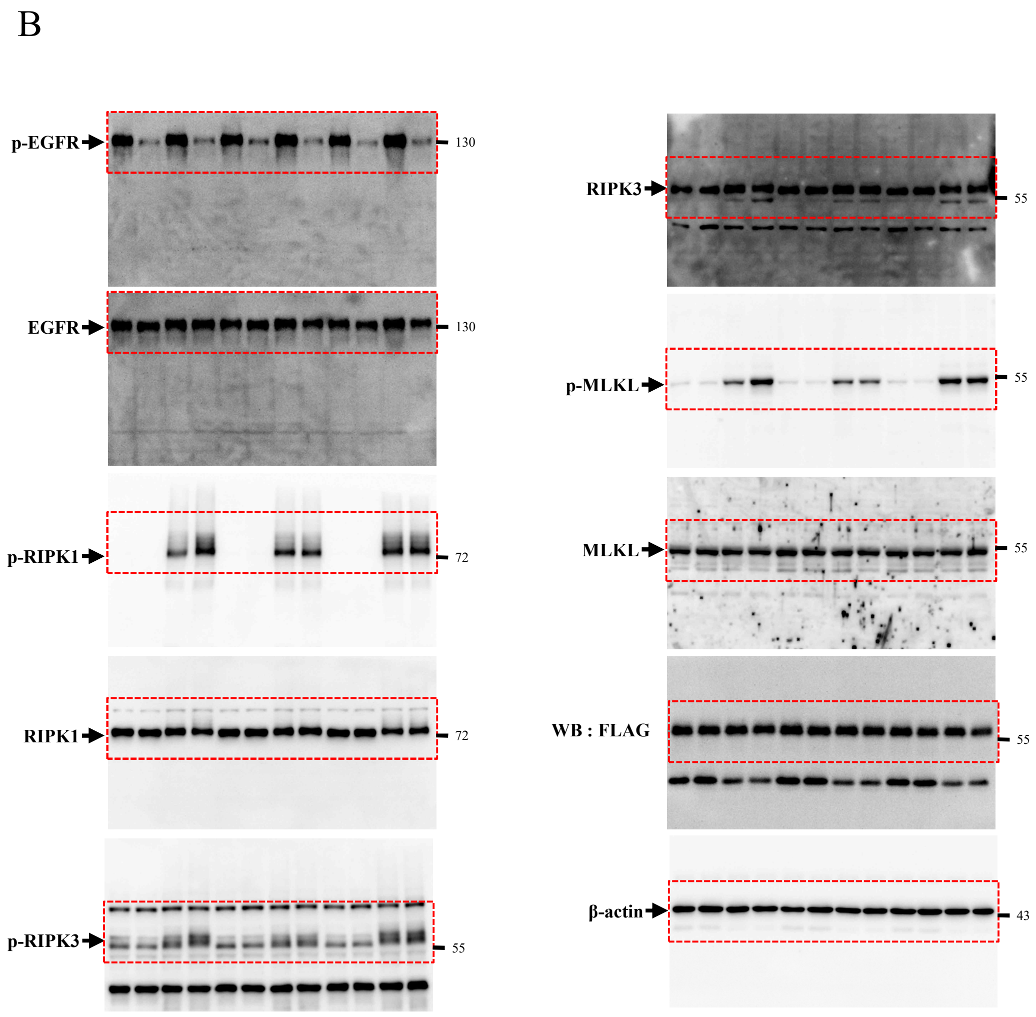

Supplementary Figure 24

B

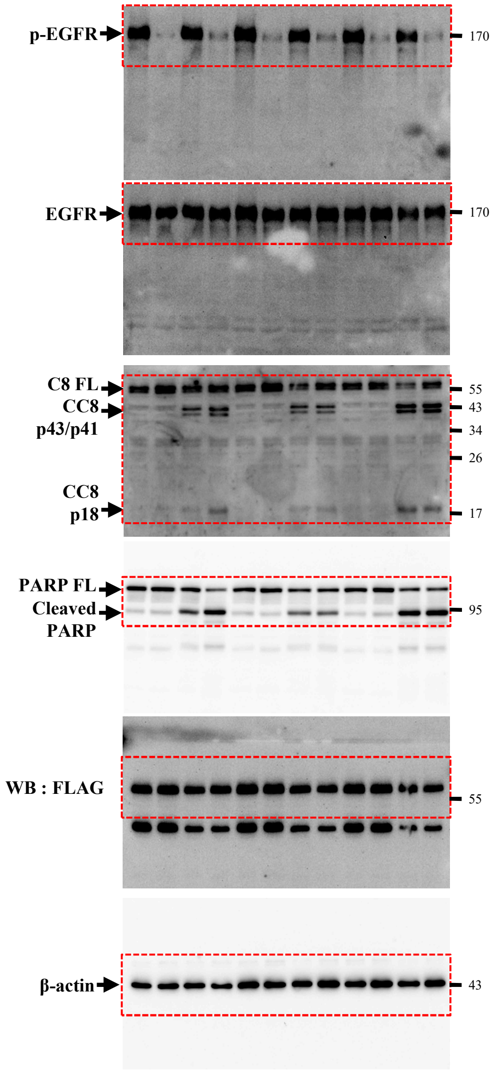

Supplement: Supplementary file 4 — original blot [file 41418_2024_1316_MOESM4_ESM.pdf]
